# GSH-responsive nanovaccine triggers immunogenic cell death and potent memory T cell immunity for durable, recurrence-free tumor eradication

**DOI:** 10.1016/j.bioactmat.2026.04.025

**Published:** 2026-04-30

**Authors:** Khang-Yen Pham, Thu Huyen Le Thi, Anil Giri, Jongjun Park, Jong-Sun Kang, Taeg Kyu Kwon, Jee-Heon Jeong, Simmyung Yook

**Affiliations:** a School of Pharmacy, Sungkyunkwan University, Suwon, 16419, Republic of Korea; b Department of Biopharmaceutical Convergence, Sungkyunkwan University, Suwon, 16419, Republic of Korea; c Department of Molecular Cell Biology, School of Medicine, Sungkyunkwan University, Suwon, 16419, Republic of Korea; d Department of Immunology, School of Medicine, Keimyung University, Daegu, 42601, Republic of Korea; e Department of Precision Medicine, School of Medicine, Sungkyunkwan University, Suwon, 16419, Republic of Korea

**Keywords:** Nanovaccines, Chemodynamic therapy, Immunotherapy, Immunogenic cell death, Long-term memory, Hollow manganese dioxide

## Abstract

Although nanovaccines hold promise for cancer immunotherapy, the engineering platforms that respond to tumor-specific cues and activate antitumor immunity remains challenging. Herein, a glutathione (GSH)-responsive immunogenic nanovaccine (SHINE) is developed, integrating immunogenic cell death (ICD)-inducing chemodynamic therapy (CDT) with immunotherapy to elicit synergistic *in situ* antitumor immunity. Constructed from a hollow MnO_2_ nanostructure, SHINE selectively degrades in GSH-enriched tumors, depleting intracellular GSH while releasing Mn^2+^ to catalyze Fenton-like reactive oxygen species generation. Concurrently, SHINE facilitates the controlled release of the TLR7/8 agonist R848 and, through surface-conjugated anti-PD-L1 antibodies, enables immune checkpoint blockade and enhances active tumor targeting. This design integrates CDT as the “initiator” and R848/anti-PD-L1 as dual immune “boosters,” thereby eliciting a synergistic cascade that drives potent ICD and promotes dendritic cell maturation and T cell priming. Transcriptomics confirms robust immune activation, while *in vivo* SHINE suppresses both primary tumor growth and lung metastasis. Remarkably, SHINE establishes durable immune memory, characterized by elevated effector memory T cells and upregulation of memory T cell-related genes, thereby conferring rechallenge protection and preventing tumor recurrence. Collectively, SHINE represents a robust *in situ* cancer nanovaccine for systemic, long-term tumor control.

## Highlights

• SHINE integrates CDT-induced ICD as an “initiator” and R848/aPD-L1 as dual immune “boosters," yielding a synergistic effect.
• SHINE achieves *in situ* tumor-selective vaccination without external stimuli.
• SHINE activates robust immunity by expanding effector memory T cells and upregulating memory T cell-associated genes.
• SHINE elicits systemic antitumor efficacy against primary tumors and lung metastases.
• SHINE establishes long-term immune memory, protecting against tumor rechallenge.

## Graphical abstract

**Figure:**
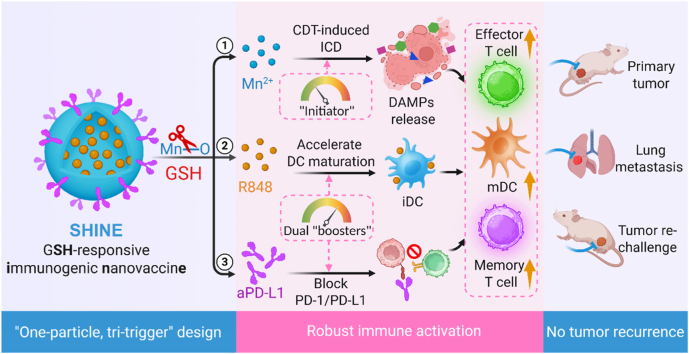

## 1 Introduction

Cancer immunotherapy, particularly immune checkpoint blockade (ICB) targeting the PD-1/PD-L1 and CTLA-4 pathways, has revolutionized oncology by offering durable responses and prolonged survival in selected patient populations [[1], [2], [3], [4], [5], [6], [7]]. However, these benefits are predominantly confined to immunologically “hot” tumors enriched with neoantigens, pre-existing T cell infiltration, and favorable immune signatures [8]. In contrast, most solid tumors remain immunologically “cold,” characterized by a suppressive tumor microenvironment (TME) that hinders antigen presentation, co-stimulatory signaling, and effector cell infiltration, thereby severely limiting the efficacy of ICB [[8], [9], [10], [11], [12], [13], [14], [15]]. Overcoming these challenges requires the design of innovative therapeutic approaches capable of reprogramming the TME and initiating robust de novo antitumor immunity.

Nanovaccines have emerged as promising tools to address these limitations by co-delivering antigens and immunomodulators with spatiotemporal precision, enhancing cross-presentation, and promoting lymphoid trafficking [[16], [17], [18], [19], [20], [21], [22], [23], [24]]. However, current designs often rely on predefined tumor-associated antigens (TAAs), which are vulnerable to tumor heterogeneity or lack TME responsiveness, increasing the risk of off-target toxicity [[25], [26], [27], [28]]. Moreover, several nanovaccines fail to synchronize antigen release with innate immunological cues, undermining their ability to prime effective adaptive responses. Thus, an ideal nanovaccine should not only elicit durable systemic immunity but also respond selectively within the TME, synchronize antigen unveiling with immune activation, and reprogram immunologically inert tumors into immune-responsive states.

*In situ* cancer vaccination offers an elegant solution by transforming the tumor into a depot of autologous TAAs *via* localized cytotoxicity [[28], [29], [30], [31], [32], [33]]. This strategy circumvents the need for exogenous antigen identification and ensures a personalized immune response. TME-responsive nanoplatforms further enhance this approach by enabling precise spatiotemporal activation in response to tumor-specific triggers such as elevated glutathione (GSH), acidic pH, or redox imbalance [25,[34], [35], [36], [37], [38]]. Among the most potent immunological mechanisms underlying *in situ* vaccination is immunogenic cell death (ICD), marked by the release of damage-associated molecular patterns (DAMPs), including calreticulin (CRT, “eat-me” signal), adenosine triphosphate (ATP, “find-me” signal), and high-mobility group box 1 (HMGB1, “danger” signal) [[39], [40], [41], [42], [43]]. Together, these signals orchestrate dendritic cell (DC) maturation, cross-priming, and T cell activation [[43], [44], [45]]. However, achieving robust ICD *in vivo* requires initiators that increase intracellular oxidative stress and boosters that amplify immune signaling and relieve checkpoint inhibition, ideally within a single responsive system.

Chemodynamic therapy (CDT) has recently emerged as a potent ICD inducer *via* Fenton or Fenton-like reactions that convert endogenous H_2_O_2_ into cytotoxic hydroxyl radicals (•OH, E°(•OH/H_2_O) = 2.80 V), inducing oxidative damage at the tumor site [40,[46], [47], [48], [49], [50], [51]]. Without the need for external stimulation, CDT has gained attention as a “green” tumor treatment offering high tumor selectivity and therapeutic precision [36,46,52,53]. However, in practice, insufficient catalytic activity, rapid reactive oxygen species (ROS) scavenging by GSH, and lack of co-delivered immunostimulants limit durable immunity [36,54]. Therefore, a rational nanovaccine design must integrate GSH consumption (to relieve ROS quenching), efficient CDT catalysis, and immune co-stimulation, triggered under the same TME conditions.

Manganese dioxide (MnO_2_) nanostructures fulfill these requirements with a minimal design complexity. Under GSH-rich conditions, MnO_2_ degrades into Mn^2+^, which not only scavenges GSH but also catalyzes •OH production *via* Fenton-like reactions, thus intensifying CDT and favoring ICD [25,36,52,[54], [55], [56], [57], [58], [59]]. The hollow MnO_2_ (h-MnO_2_) architecture further provides high drug-loading capacity [[60], [61], [62]]. When paired with immunostimulants such as resiquimod (R848), a TLR7/8 agonist that activates DCs and promotes Th1 polarization [16,[63], [64], [65], [66], [67]], and functionalized with anti-PD-L1(aPD-L1) antibodies to relieve checkpoint blockade [[68], [69], [70]], such a platform can coordinate multiple immune mechanisms. Despite these advantages, only a few nanovaccines have successfully integrated these synergistic modalities into MnO_2_-based, GSH-responsive nanoplatforms. Moreover, such systems have rarely demonstrated long-lasting immune memory that supports durable antitumor immunity.

**Scheme 1:**
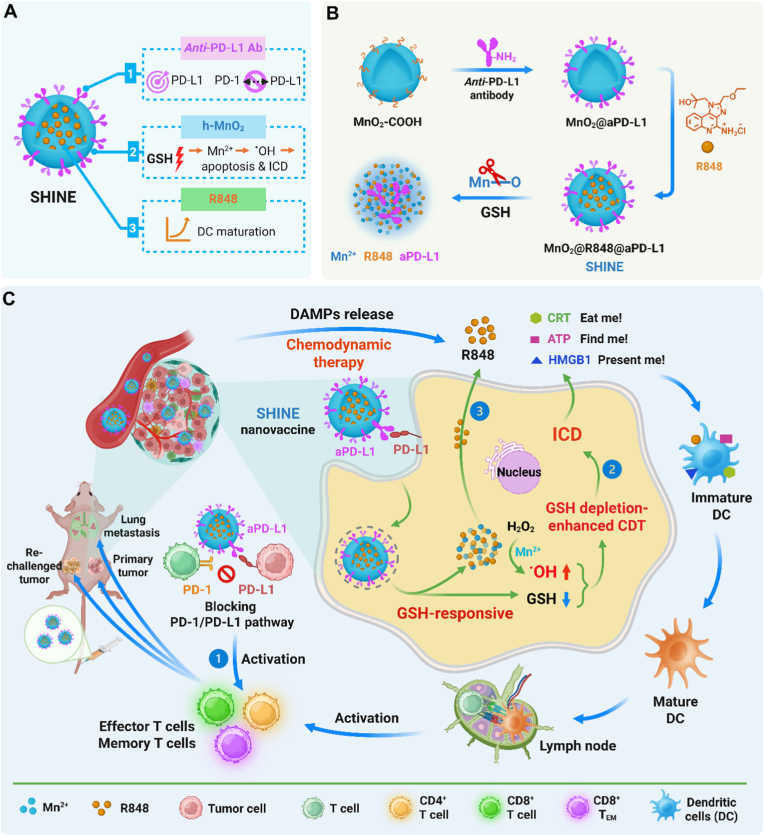
**Design strategy and anti-tumor immune responses of the SHINE nanovaccine.** (**A**) Design strategy for the SHINE nanovaccine. (**B**) The preparation process of the SHINE nanovaccine: biodegradable hollow MnO_2_ NPs conjugated with aPD-L1 antibody and loaded with resiquimod (R848). (**C**) Mechanism of action: (1) The aPD-L1 antibody component of SHINE blocks the PD-1/PD-L1 immune checkpoint pathway, thereby promoting T cell activation. (2) Once internalized into tumor cells, MnO_2_ reacts with intracellular GSH to release Mn^2+^, which catalyzes the conversion of endogenous H_2_O_2_ into cytotoxic •OH radicals, inducing chemodynamic therapy (CDT) and immunogenic cell death (ICD). ICD promotes the release of damage-associated molecular patterns (DAMPs), leading to dendritic cell (DC) activation and enhanced antigen presentation. (3) Simultaneously, MnO_2_ degradation releases R848, which activates TLR7/8 signaling and promotes DC maturation. Overall, these effects amplify the anti-tumor immune response. Created with BioRender.com.

We herein strategically engineered SHINE, a GSH-responsive immunogenic nanovaccine that integrates ICD-inducing CDT as an “initiator” with dual immune activation by R848 and aPD-L1 as “boosters” within a single, unified nanomedicine (Scheme 1). SHINE simultaneously depletes intracellular GSH and releases Mn^2+^ to amplify CDT, while liberating R848 to promote DC maturation. In parallel, SHINE displays aPD-L1 on its surface, enabling active tumor targeting and concentrating checkpoint blockade at the tumor–immune interface, thereby relieving PD-L1-mediated suppression precisely where DAMPs and TAAs emerge. This “one-particle, tri-trigger” design enables SHINE to achieve tumor-selective *in situ* cancer vaccination by orchestrating a synergistic cascade within the TME, without relying on exogenous triggers. Accordingly, both CDT activity and immune stimulation remain largely inactive in circulation but are switched on within the TME, thereby minimizing off-target effects. Flow cytometry and transcriptomic analyses revealed that SHINE elicits robust immune activation and induces systemic antitumor immunity against both primary tumors and lung metastases. Notably, SHINE establishes durable immune memory, as evidenced by expanded effector memory T cell populations and upregulated memory T cell-associated gene programs, which together confer protection against tumor rechallenge. This feature distinguishes SHINE from most GSH-responsive MnO_2_-based nanoplatforms that primarily focus on primary tumor suppression through ROS-mediated CDT or transient immune stimulation, with limited capacity to generate long-term antitumor immunity. SHINE exemplifies a TME-triggered, ICD-driven *in situ* cancer nanovaccine that coordinates an “initiator” and immune “boosters” within a single GSH-responsive nanoparticle, offering a blueprint for next-generation nanomedicines to achieve durable responses, control metastasis, and prevent tumor recurrence.

## 2 Results

### 2.1 Construction and characterization of SHINE nanovaccine

A three-step process was followed to synthesize aPD-L1–conjugated and R848-loaded hollow MnO_2_ nanoparticles (MnO_2_@R848@aPD-L1, abbreviated as SHINE). First, biodegradable hollow MnO_2_ (h-MnO_2_) nanoshells were prepared using SiO_2_ nanoparticles as a hard template [60]. A PAH/PAA polyelectrolyte coating was subsequently applied to the h-MnO_2_ surface *via* a layer-by-layer technique. Next, H_2_N–PEG–COOH was conjugated to the surface using an EDC/NHS coupling reaction to generate PEGylated h-MnO_2_ (denoted as M) (Fig. S1). Second, aPD-L1 antibodies were covalently attached to the surface of M through amide bond formation, yielding MnO_2_@aPD-L1 nanoparticles (MP). Finally, TLR7/8 agonist R848 was loaded onto the MP nanoparticles by incubating under gentle stirring, resulting in the formation of the SHINE nanovaccine (MnO_2_@R848@aPD-L1) (Scheme 1B).

**Fig. 1:**
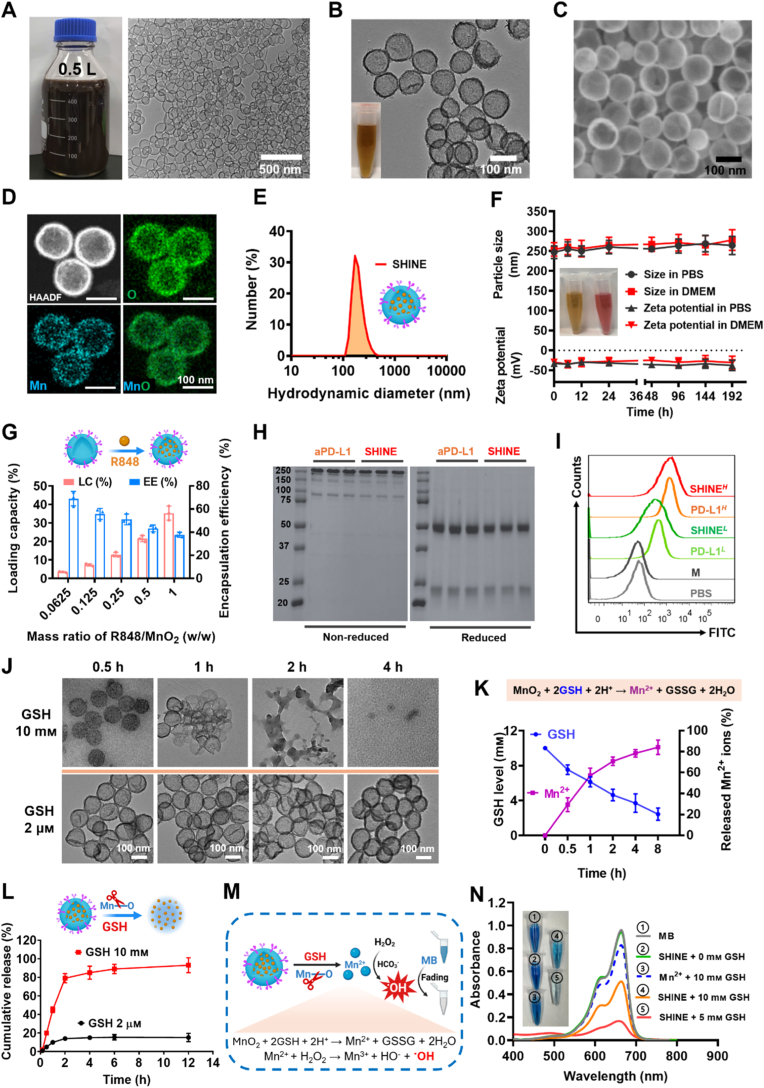
**Characterization of the SHINE nanovaccine.** (**A**) Photograph of large-scale synthesis of hollow MnO_2_ nanoparticles using a 0.5 L reaction. (**B, C**) Representative TEM and SEM images showing the hollow spherical morphology of SHINE. (**D**) HAADF-STEM image and elemental mapping of h-MnO_2_, confirming the distribution of O and Mn throughout the nanoparticle shell. (**E**) Hydrodynamic size distribution of SHINE. (**F**) Hydrodynamic diameter and zeta potential of SHINE in PBS and DMEM over time, demonstrating colloidal stability under these conditions. (**G**) Loading capacity (LC%) and encapsulation efficiency (EE%) of R848 in SHINE at different R848/MnO_2_ mass ratios. (**H**) SDS-PAGE analysis of free aPD-L1 antibody and aPD-L1-conjugated SHINE under non-reduced and reduced conditions, confirming successful antibody conjugation. (**I**) Flow cytometric analysis of immunoreactivity, showing preserved binding activity of aPD-L1 after conjugation to SHINE. *L*, low concentration (aPD-L1-equivalent 10 μg mL^−1^); *H*, high concentration (aPD-L1-equivalent 20 μg mL^−1^). (**J**) TEM images showing GSH-responsive biodegradation of SHINE over time under 10 mM or 2 μM GSH conditions. (**K**) Time-dependent GSH consumption and Mn^2+^ release during GSH-triggered SHINE degradation. (**L**) Cumulative R848 release from SHINE in the presence of 10 mM or 2 μM GSH, demonstrating GSH-responsive cargo release. (**M**) Schematic illustration of methylene blue (MB) degradation mediated by •OH generated from Mn^2+^ through a Fenton-like reaction. (**N**) Absorbance spectra showing extracellular •OH generation after SHINE degradation under GSH-containing conditions, as indicated by MB bleaching. M, MnO_2_@PEG; SHINE, MnO_2_@R848@aPD-L1. Data are presented as mean ± SD (*n = 3*). Panel M created with BioRender.com.

We then established the structural and functional integrity of the SHINE nanovaccine. As shown in Fig. 1A, h-MnO_2_ nanoshells were synthesized on a large scale of 0.5 L with high quality, in one reaction batch. Transmission electron microscopy (TEM) (Fig. 1B) and scanning electron microscopy (SEM) (Fig. 1C) images of SHINE revealed uniformly dispersed spherical nanoparticles with a well-defined hollow interior, an average diameter of ∼110 nm, and a shell thickness of ∼12 nm. High-angle annular dark-field scanning transmission electron microscopy (HAADF-STEM) image with elemental mapping showed concentric shells enriched for Mn and O, with spatially co-localized signals consistent with MnO_2_ distributed throughout the shell wall and a void interior, substantiating the hollow nanostructure that affords internal free volume for cargo (Fig. 1D). X-ray photoelectron spectroscopy (XPS) was used to verify the surface composition and Mn chemical state of SHINE nanoparticles. The survey spectrum shows clear Mn and O signals together with strong C 1s and N 1s contributions (Fig. S2A). High-resolution Mn 2p spectra display two characteristic peaks at 654.5 eV (Mn 2p_1/2_) and 642.8 eV (Mn 2p_3/2_), giving a spin-orbit separation of 11.7 eV (Fig. S2B). In addition, the Mn 3s region exhibits the diagnostic multiplet splitting, supporting a high Mn oxidation state consistent with MnO_2_-type species (Fig. S2C). The broadened O 1s envelope is attributable to lattice Mn–O and higher-binding-energy oxygen species from surface hydroxyl and PAA/PEG/aPD-L1 functionalities (Fig. S2D). The pronounced C 1s and N 1s signals, with bonding environments attributable to PEG ether/carboxylate and amide/amine functionalities, further corroborate successful surface functionalization of MnO_2_ by PAH/PAA/PEG/aPD-L1 (Fig. S2E and F). Dynamic light scattering revealed a narrow unimodal hydrodynamic size distribution with an average diameter of 253 nm (polydispersity index: 0.12), while the ζ-potential measurement (−35 mV) indicated colloidal stability in aqueous dispersion (Fig. 1E and F). Notably, the size and dispersity remained stable across the tested environments over time, indicating resistance to salt- and protein-induced aggregation, which is an essential prerequisite for reliable *in vivo* performance (Fig. 1F). In addition, SHINE also showed acceptable long-term colloidal stability over 21 days, with minimal changes in H_2_O and only a gradual increase in size under in 10% FBS, without obvious precipitation (Fig. S3). SHINE exhibited a strong emission peak at 339 nm, consistent with that of free R848, confirming the successful encapsulation of R848 within the MnO_2_ NPs (Figs. S4 and 5). R848 loading studies demonstrated a high loading capacity (∼24%) and an encapsulation efficiency of ∼42% at an R848/MnO_2_ mass ratio of 0.5, indicating the suitability of the formulation for further investigation (Fig. 1G and Fig. S6). We next quantified the amount of surface-conjugated aPD-L1 using a standard protein assay. For the selected formulation, the antibody loading was 112.6 ± 8.7 μg aPD-L1 per mg SHINE NPs at an initial aPD-L1 concentration of 75 μg mL^−1^ (Fig. S7). In addition, based on this loading amount and the TEM-derived dimensions of the hollow SHINE NPs, the average number of surface-conjugated aPD-L1 molecules was estimated to be approximately 495 per nanoparticle (Note S1).

The antibody presentation and functionality were verified using orthogonal assays. SDS-PAGE analysis demonstrated successful conjugation of aPD-L1 antibodies onto the MnO_2_ surface, with the appearance of antibody bands in the SHINE lane under both non-reducing and reducing conditions (Fig. 1H). Flow cytometry further demonstrated that the surface-conjugated aPD-L1 retained its immunoreactivity post-conjugation. The fluorescence intensity of the SHINE group was comparable to that of free aPD-L1 antibody at both low and high concentrations, indicating preserved PD-L1-binding activity after surface modification (Fig. 1I). Although this assay does not provide equilibrium binding affinity constants, it confirms that the conjugation process did not abolish PD-L1-recognition capability.

Together, these data verify that SHINE is a monodisperse hollow MnO_2_ nanovaccine with high R848 payload, durable colloidal stability, and sustained PD-L1-targeting capability, fulfilling key design criteria for effective tumor-targeted immunotherapy.

### **2.2** **GSH-responsive biodegradation and Mn^2+^-**m**ediated** •**OH generation by SHINE**

Next, we verified GSH-triggered disassembly and chemodynamic competence of SHINE. In 10 mм GSH, time-lapse TEM showed sequential shell thinning, rim rupture, and collapse of the h-MnO_2_ nanoparticles, whereas intact morphology was observed in 2 μм GSH solution (Fig. 1J and Fig. S8). Quantitation revealed a tight inverse relationship between GSH consumption and Mn^2+^ accumulation over time, indicating stoichiometric reduction of MnO_2_ to Mn^2+^ during biodegradation (Fig. 1K). R848 releases couples to this disassembly (Fig. S9). In the presence of 10 mм GSH, SHINE exhibits rapid and sustained R848 liberation; in its absence, release is minimal, demonstrating that the MnO_2_ shell functions as a redox gate that secures the payload until it encounters a reducing environment (Fig. 1L).

To evaluate the link between MnO_2_ scaffold reduction and ROS generation, methylene blue (MB), a chromogenic probe for •OH, was used in NaHCO_3_/CO_2_ buffer [52,54]. Upon exposure to GSH, MnO_2_ is reduced to Mn^2+^, which subsequently catalyzes Fenton-like reactions with H_2_O_2_ present in the tumor-mimicking environment, generating •OH that oxidatively bleaches MB (Fig. 1M). As shown in Fig. S10A, MB absorbance remained largely unchanged when treated with H_2_O_2_ or Mn^2+^ alone. In contrast, the combination of MnCl_2_ and H_2_O_2_ induced a significant decrease in the MB absorbance, confirming effective •OH production *via* Mn^2+^/H_2_O_2_ in a bicarbonate-rich buffer. Notably, addition of 10 mм GSH markedly attenuated MB degradation, consistent with the known •OH-scavenging activity of GSH (Fig. S10A). This observation highlights a critical limitation of CDT in GSH-rich tumor environments, where intracellular antioxidants can quench cytotoxic radicals and diminish therapeutic efficacy.

**Fig. 2:**
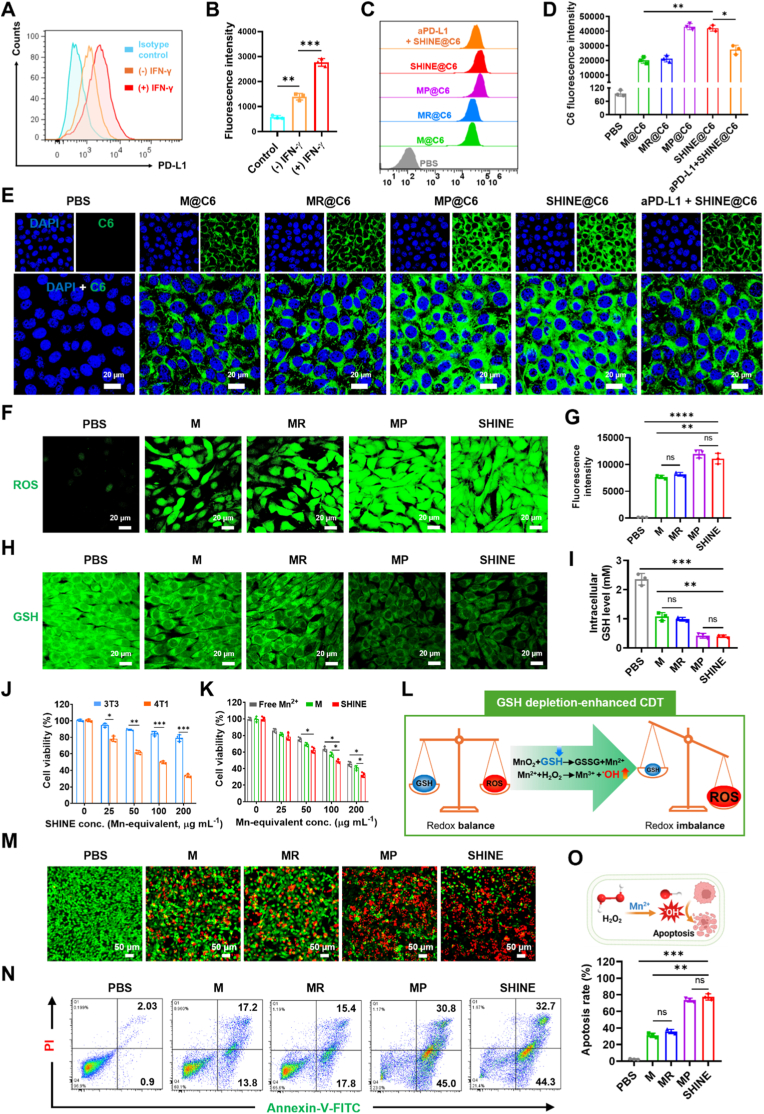
**Intracellular behavior of SHINE nanovaccine**. (**A, B**) Flow cytometric analysis and quantification of PD-L1 expression in 4T1 cells with or without IFN-γ stimulation, confirming successful upregulation of PD-L1 prior to evaluation of targeted cellular uptake. (**C, D**) Representative flow cytometric histograms and quantitative analysis of cellular uptake of C6-labeled formulations in 4T1 cells, showing enhanced uptake of SHINE in PD-L1-expressing cells and reduced uptake after competitive blocking with free aPD-L1. (**E**) Confocal laser scanning microscopy (CLSM) images further confirming enhanced cellular internalization of SHINE in 4T1 cells. (**F, G**) CLSM images and corresponding quantification of intracellular ROS levels after 5 h of treatment, showing increased ROS generation in the SHINE-treated group. (**H, I**) CLSM images and quantitative analysis of intracellular GSH levels after 5 h of treatment, indicating effective intracellular GSH depletion by SHINE. (**J**) Cell viability of normal 3T3 cells and cancerous 4T1 cells after 24 h incubation with SHINE at different Mn-equivalent concentrations, showing preferential cytotoxicity toward 4T1 cells while relatively sparing normal cells and supporting selection of the working concentration for subsequent *in vitro* experiments. (**K**) Cell viability of 4T1 cells after 24 h treatment with free Mn^2+^, MnO_2_@PEG (M), or SHINE at matched Mn-equivalent concentrations, showing that GSH depletion and the full SHINE formulation confer stronger cytotoxicity than Mn^2+^ alone. (**L**) Schematic illustration of the mechanism of GSH depletion-enhanced CDT mediated by SHINE. (**M**) Live/dead staining images of 4T1 cells after 24 h treatment with different formulations, showing increased cancer cell death in the SHINE-treated group. Live and dead cells are shown in green and red, respectively. (**N, O**) Representative flow cytometric plots and quantitative analysis of apoptosis in 4T1 cells after 24 h treatment with different formulations, confirming that SHINE induces pronounced apoptotic cell death. Data are presented as mean ± SD (*n = 3*; *n = 4* for (J) and (K)). ∗*p*< 0.05, ∗∗*p*< 0.01, ∗∗∗*p*< 0.001, ∗∗∗∗*p* < 0.0001; n.s., not significant (*p* > 0.05).

We next examined whether the GSH-responsive design of SHINE could overcome this barrier by simultaneously releasing Mn^2+^ as a Fenton-like catalyst and depleting GSH, the major •OH scavenger. Upon incubation with GSH and H_2_O_2_, SHINE induced pronounced MB degradation. As shown in Fig. 2N, increasing GSH concentrations from 0 to 5 mм led to an increase in MB degradation, indicating GSH-triggered release of catalytically active Mn^2+^. At 10 mм GSH, the extent of degradation was slightly reduced due to competitive radical scavenging by excess GSH. Importantly, under tumor-relevant GSH conditions (10 mм), SHINE achieved ∼45% MB degradation, compared with ∼14% for free Mn^2+^, representing a ∼3.2-fold enhancement (Fig. S10B). These results demonstrate that SHINE not only supplies Mn^2+^ for Fenton-like catalysis but also depletes GSH, establishing a positive feedback loop that limits radical quenching and amplifies CDT efficacy.

Collectively, these findings indicate that intracellularly relevant GSH degrades the MnO_2_ shell, depletes GSH, releases Mn^2+^, and unlocks R848; the emergent Mn^2+^ then drives Fenton-like ROS generation. This tight linkage between R848 release and localized oxidative stress renders SHINE a triggerable nanovaccine engineered to remodel the TME and synergize with PD-L1 blockade in subsequent biological studies.

### 2.3 Cellular uptake of the SHINE nanovaccine

We examined the mechanism by which SHINE affects 4T1 cells. Fluorescence-activated cell sorting (FACS) confirmed abundant PD-L1 on 4T1 cells, which increased further after IFN-γ priming (Fig. 2A and B). We then quantified the nanoparticle uptake using Coumarin-6 (C6)-labeled formulations. FACS analysis revealed fluorescence intensities in the following order: PBS ≪ M@C6 ≈ MR@C6 < MP@C6 ≈ SHINE@C6, indicating that antibody functionalization is the primary contributor to enhanced cellular uptake (Fig. 2C and D). The cellular uptake of SHINE@C6 improved by 2.2-fold (*p* < 0.01) compared with that of MR@C6 NPs. Pre-incubation with free aPD-L1 markedly reduced SHINE@C6 internalization (1.8-fold, *p* < 0.05), which is consistent with competitive receptor engagement and a PD-L1-dependent entry route. Confocal microscopy corroborated these trends; SHINE@C6 produced intense fluorescence that diminished upon PD-L1 blocking, whereas M@C6 and MR@C6 yielded weaker signals (Fig. 2E). Collectively, these data demonstrate that PD-L1 conjugation substantially boosts the cellular delivery of SHINE in PD-L1-high tumor cells *via* the PD-L1 receptor-mediated transcytosis pathway.

### 2.4 Intracellular redox remodeling by SHINE nanovaccine

We examined whether the SHINE nanovaccine modulated redox homeostasis in 4T1 cells. Using DCFH-DA as an ROS reporter, CLSM showed negligible fluorescence with PBS, a modest signal with M or MR, and a marked increase in PD-L1-decorated formulations (MP, SHINE) (Fig. 2F). Quantitative analysis confirmed that SHINE produced 9.1-fold higher ROS than control (*p* < 0.0001) and 1.5-fold higher than M or MR (*p* < 0.01), consistent with PD-L1-guided uptake that accelerates MnO_2_ reduction to Mn^2+^ and subsequent Fenton-like reactions (Fig. 2G). We assessed intracellular GSH levels, an essential component of the cellular antioxidant system, using a thiol-sensitive probe (ThiolTracker). Untreated cells exhibited bright, diffuse cytosolic staining, whereas the signal intensity declined for M and MR and reached a minimum with SHINE (Fig. 2H). Quantitative analysis revealed that SHINE reduced cellular GSH by 8.3-fold compared to the control (*p* < 0.001) and by 3.3-fold relative to M or MR (*p* < 0.01) (Fig. 2I). This depletion aligns with the stoichiometric reduction of MnO_2_ by GSH (MnO_2_+ 2GSH + 2H^+^ → Mn^2+^+ GSSG + 2H_2_O), which both consumes GSH and generates catalytic Mn^2+^ to amplify ROS. Taken together, these data establish SHINE as an effective redox-remodeling nanovaccine that integrates GSH depletion with Mn^2+^-mediated ROS amplification, thereby enhancing its therapeutic potential in nanomedicine applications.

### 2.5 GSH depletion-enhanced CDT of the SHINE nanovaccine

We evaluated the *in vitro* chemodynamic effect of SHINE. Dose–response assays showed minimal toxicity to normal 3T3 fibroblasts but potent activity in 4T1 cancer cells. Specifically, in 3T3 cells, viability remained high across the tested range (>80% even at 200 μg mL^−1^), indicating good biocompatibility (Fig. 2J). In contrast, 4T1 cell viability declined steeply with increasing doses, falling to 49% at 100 μg mL^−1^ and 35% at 200 μg mL^−1^, demonstrating tumor selectivity. To clarify the mechanistic advantage of SHINE, we compared Mn^2+^, MnO_2_@PEG (denoted as M), and SHINE in 4T1 cells (Fig. 2K). Here, Mn^2+^ represents CDT alone, M represents CDT coupled with GSH depletion, and SHINE further incorporates aPD-L1-mediated targeting to 4T1 cells. At 100 μg mL^−1^, cell viability was 65% for Mn^2+^, 57% for M, and 48% for SHINE; at 200 μg mL^−1^, the corresponding values were 50%, 41%, and 32%, respectively. SHINE exhibited the strongest cytotoxicity among the tested groups, reflecting the combined contribution of the MnO_2_ core, which both depletes intracellular GSH and generates Fenton-active Mn^2+^ [52,71,72], and aPD-L1-mediated tumor cell targeting. Mechanistically, intracellular GSH reduces MnO_2_ to Mn^2+^, simultaneously (i) consuming the cellular antioxidant component, thereby preventing the scavenging effect of GSH, and (ii) supplying catalytic Mn^2+^ to convert H_2_O_2_ into •OH, tipping the redox balance toward lethal oxidative damage (Fig. 2L). Collectively, these findings indicate that SHINE enables GSH depletion-enhanced, tumor-targeted CDT, achieving more potent cytotoxicity than Mn^2+^ alone or MnO_2_, while sparing normal cells.

Next, we quantified the cytotoxic effects of SHINE in 4T1 cells using live/dead cell staining and apoptosis assays. Calcein-AM/PI staining showed that PBS-treated cells remained predominantly viable and retained their typical adherent morphology, whereas M and MR increased the proportion of PI-positive cells. Notably, the PD-L1-decorated formulations (MP and SHINE) induced markedly greater cell death, accompanied by reduced live-cell density and pronounced disruption of adherent cell morphology (Fig. 2M). Annexin V–FITC/PI flow cytometry confirmed that apoptosis was the dominant mode of cell death (Fig. 2N and O). The total apoptotic fraction (early apoptosis + late apoptosis) increased from 3.2% (PBS) to 30.3% (M) and 32.6% (MR), and further to 76.9% (MP) and 79.4% (SHINE), indicating that PD-L1 targeting augmented MnO_2_-driven CDT, whereas R848 loading did not cause cell death. We assessed tumor cell motility using a wound-healing assay (Fig. S11). After 24 h, the relative wound closure (normalized to PBS = 100%) decreased to 60% (M, MR), 29% (MP), and 15% (SHINE), demonstrating that SHINE most effectively suppressed migration. Collectively, these results support a mechanism in which PD-L1-guided uptake enhances intracellular MnO_2_ reduction and Mn^2+^-mediated •OH generation, driving apoptotic death and limiting motility, which are two hallmarks of chemodynamic and anti-metastatic activity.

### 2.6 CDT induces hallmarks of ICD in 4T1 cells

**Fig. 3:**
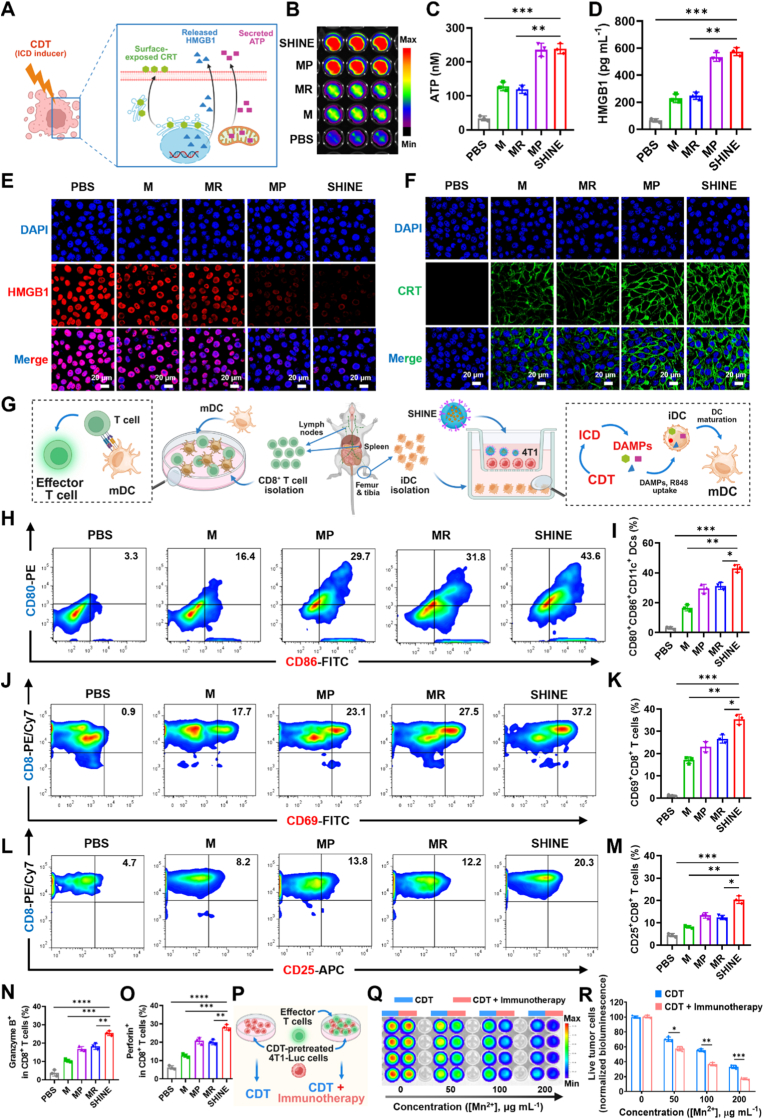
***In vitro* evaluation of CDT-induced immunogenic cell death (ICD), dendritic cell (DC) maturation, and T cell activation**. (**A**) Schematic illustration of CDT-triggered ICD and associated damage-associated molecular pattern (DAMP) release. (**B, C**) Bioluminescence images and quantitative analysis of extracellular ATP release from 4T1 cells after different treatments. (**D, E**) Quantitative analysis and CLSM images of HMGB1 release. (**F**) CLSM images showing CRT surface exposure in 4T1 cells after different treatments. (**G**) Schematic illustration of the co-culture system used to evaluate DC maturation and subsequent T cell activation. Immature DCs (iDCs) were isolated from bone marrow, and CD8^+^ T cells were isolated from the lymph nodes and spleens of naïve BALB/c mice. (**H, I**) Representative flow cytometric plots and quantitative analysis of mature DCs, defined as CD11c^+^CD80^+^D86^+^ cells, after 24 h incubation with differently treated 4T1 cells, showing enhanced DC maturation in the SHINE group. (**J, K**) Representative flow cytometric plots and quantification of CD69^+^ cells among CD8^+^ T cells. (**L, M**) Representative flow cytometric plots and quantification of CD25^+^ cells among CD8^+^ T cells. (**N, O**) Quantification of granzyme B and perforin expression in CD8^+^ T cells, indicating enhanced T cell activation and effector function following SHINE treatment. (**P**) Schematic illustration of the *in vitro* tumor-killing assay. CDT-pretreated 4T1-Luc cells (CDT only) and CDT-pretreated 4T1-Luc cells co-cultured with effector CD8^+^ T cells at an effector-to-target (E:T) ratio of 5:1 were used to evaluate the additional contribution of immunotherapy. After 24 h, D-luciferin (150 μg mL^−1^) was added to quantify live tumor cells by luciferase activity. (**Q, R**) Bioluminescence images and quantitative analysis of surviving 4T1-Luc cells after treatment with CDT alone or combined CDT + immunotherapy at different concentrations, showing that addition of immunotherapy further enhanced tumor cell killing. M, MnO_2_@PEG; MR, MnO_2_@R848; MP, MnO_2_@aPD-L1; SHINE, MnO_2_@R848@aPD-L1. Data are presented as mean ± SD (*n = 3*; *n = 4* for (R)). ∗*p*< 0.05, ∗∗*p*< 0.01, ∗∗∗*p* < 0.001. Panels A, G, and P created with BioRender.com.

Inspired by the superior CDT performance of SHINE, we evaluated whether elevated ROS levels and apoptosis induced by SHINE could elicit ICD effects. ICD hallmarks are the secretion of distress signaling DAMPs, including CRT, HMGB1, and ATP (Fig. 3A) [39,40,42,43,45,73,74]. Using an ATP reporter, bioluminescence imaging revealed stronger extracellular ATP signals with MnO_2_-based formulations than with PBS, with the most intense signal observed in PD-L1-decorated groups (MP and SHINE) (Fig. 3B). Quantitative analysis corroborated this trend: the ATP concentration increased from ∼40 nM (PBS) to 120 nM for M/MR and 250 nM for MP/SHINE (*p* < 0.001 *vs.* PBS; *p* < 0.01 *vs.* M or MR) (Fig. 3C). Next, we evaluated HMGB1, a biomarker that is translocated from the nucleus into the extracellular milieu, where it binds to DCs and activates immune signaling pathways. ELISA showed HMGB1 rising from ∼80 pg mL^−1^ (PBS) to 270 pg mL^−1^ (M/MR) and 580 pg mL^−1^ (MP/SHINE) (*p* < 0.001 *vs.* PBS; *p* < 0.01 *vs.* M or MR) (Fig. 3D). Concordantly, CLSM revealed diminished nuclear HMGB1 staining, which was most pronounced for SHINE, indicating rapid translocation of HMGB1 from the nucleus to the cytoplasm (Fig. 3E). Finally, CRT immunofluorescence demonstrated minimal signal in PBS-treated cells, moderate membrane localization of M and MR, and strong, continuous surface CRT in MP/SHINE, marking efficient presentation of “eat-me” signals to phagocytes (Fig. 3F). Together, these DAMPs substantiate a coherent CDT-initiated ICD program that couples tumor cell injury with ATP secretion, HMGB1 release, and surface CRT exposure. Consequently, the redox-chemodynamic activity of SHINE not only eliminates PD-L1-high tumor cells but also converts them into a pro-immunogenic substrate, providing a mechanistic foundation for DC maturation and downstream T cell priming. Therefore, effective ICD implantation is expected to enable *in situ* cancer vaccination.

### 2.7 CDT-driven maturation of DCs in a 4T1–DC co-culture and STING-associated activation

Before performing immune activation-related assays, we evaluated whether SHINE affects immune-cell viability given the role of GSH in redox homeostasis. BMDCs and CD8^+^ T cells maintained high viability after 24 h exposure to SHINE across 0–200 μg mL^−1^ (Mn-equivalent), remaining ∼82–85% even at 200 μg mL^−1^ (Fig. S12A and B). To evaluate whether SHINE-elicited ICD promoted professional antigen presentation, we co-cultured bone marrow–derived immature DCs (iDCs) with 4T1 cells subjected to the indicated treatments (Fig. 3G). DC maturation was quantified by flow cytometry as CD80^+^CD86^+^ within the CD11c^+^ gate after 24 h (Figure S13 and Fig. 3H and I). MnO_2_ NPs (M) increased maturation to 16.5% (4.7-fold *vs.* PBS), consistent with CDT-induced ICD providing DAMPs for iDC activation. Incorporation of the TLR7/8 agonist (MR) or PD-L1 targeting (MP) further elevated maturation to 31.1% and 29.4%, respectively (8.4–8.8-fold *vs.* PBS), indicating that either adjuvating with R848 or enhancing tumor engagement augments the ICD cue. Notably, the full formulation, combining MnO_2_, R848, and aPD-L1 (SHINE), yielded the highest maturation rate of 43.1% (12.3-fold *vs.* PBS; 1.5- and 1.4-fold *vs.* MR and MP, respectively). Collectively, these findings indicate that SHINE drives the robust upregulation of the co-stimulatory ligands CD80 and CD86 on CD11c^+^ DCs *in vitro*, which represents an essential step toward the efficient priming of effector T cell responses.

To further elucidate innate immune mechanisms beyond CDT-mediated ICD, we examined whether SHINE activates STING-associated signaling, given that GSH-responsive MnO_2_ degradation can release Mn^2+^, which has been reported to potentiate cytosolic DNA sensing and type I interferon programs (Fig. S14A) [75,76]. In mouse BMDCs, SHINE markedly increased STING phosphorylation (p-STING) and downstream interferon regulatory factor 3 (IRF3) phosphorylation (p-IRF3) relative to the PBS negative control, while total STING and IRF3 levels remained comparable (Fig. S14B). Consistently, SHINE significantly elevated type I interferon (IFN-β) secretion, reaching a level comparable to MnCl_2_ (positive control) (Fig. S14C), supporting that SHINE-derived Mn^2+^ contributes to this innate immune activation. Together, these results indicate that SHINE not only promotes DC maturation through CDT-driven tumor damage and ICD cues but also engages a STING-IRF3-type I IFN axis in DCs, which may further facilitate DC licensing for downstream T-cell priming.

### 2.8 T cell activation downstream of SHINE-elicited ICD

We tested whether SHINE-driven DC maturation translates into robust CD8^+^ T cell activation. To evaluate T cell activation, we measured the expression levels of activation markers, including early (CD69) and late (CD25) [77,78], and cytokine generation, including perforin and granzyme B (GZB) production, in SHINE-treated CD8^+^ T lymphocytes.

CD69 and CD25 activation markers were quantified using flow cytometry within the CD8^+^ gates (Figure S15 and Fig. 3J–M). MnO_2_ NPs-treated (M) activate 15.4% CD69^+^/CD8^+^ population and further increase with R848 loading (MR, 26.3%). PD-L1 decoration enhanced activation (MP, 24.1%), and the full formulation (SHINE) was the most effective (38.1%, 38.1-fold *vs*. PBS; 2.5-fold *vs.* M; 1.4-fold *vs.* MR, MP) (Fig. 3J and K). A similar pattern was observed for CD25 (Fig. 3L and M). Compared with M (7.8%), the percentage of CD25^+^/CD8^+^ T cells increased in the MR and MP treatment groups (12-14%, 1.5-fold, *p* < 0.05) and peaked in the SHINE-treated cells (19.3%, 2.5-fold, *p* < 0.01). Thus, MnO_2_-mediated ICD is sufficient to prime activation, while R848 adjuvancy and PD-L1 targeting provide additive gains, with SHINE giving the strongest T cell activation.

The release of cytokines upon stimulation of T cells is a typical indicator of an immune response [79]. We then examined the levels of the released cytokines. Quantification showed increases in GZB (Fig. 3N) and perforin (Fig. 3O) levels, mirroring the activation markers. Relative to PBS, cells treated with M, MR, MP, and SHINE increased GZB to 2.2-fold (*p* < 0.05), 4.6-fold (*p* < 0.01), 5.1-fold (*p* < 0.001), and 6.5-fold (*p* < 0.001), respectively. Perforin displayed a similar trend, in which the release level increased from 12.3% (M) to 20.4-21.5% (MP, MR), and then to 26.7% (SHINE).

In summary, these data demonstrate that SHINE converts chemodynamic tumor-cell damage into potent adaptive immunity, where PD-L1-guided delivery and R848 adjuvancy synergize with MnO_2_-triggered ICD, with STING-associated type I IFN signaling providing an additional innate immune amplifier, to maximize CD8^+^ T cell activation and induce the release of high cytotoxic cytokines.

### 2.9 Combined chemodynamic–immunotherapy enhances tumor-cell killing by the SHINE nanovaccine *in vitro*

To determine whether the immunostimulatory design of SHINE augments chemodynamic killing, we compared CDT alone (4T1-Luc monoculture) with CDT + immunotherapy (co-culture of 4T1-Luc and effector CD8^+^ T cells) using a luciferase-based viability readout after 24 h (Fig. 3P). We used the luciferase assay to specifically quantify tumor cell viability [80]. In contrast, CCK-8 was used to report the aggregate metabolic activity of both tumor and immune cells in the well. As expected, SHINE reduced bioluminescence in a dose-dependent manner in both settings, with consistently stronger suppression in the co-culture, indicating the immune-mediated amplification of tumor cell killing (Fig. 3Q). As shown in Fig. 3R, under CDT-only conditions, the luciferase activity decreased to 70.6% and 56.3% at 50 and 100 μg mL^−1^ Mn, respectively. Under CDT + immunotherapy, the corresponding values were 56.8% and 37.4%, respectively. At a higher Mn concentration (200 μg mL^−1^), the viability was 32.7% with CDT alone *vs.* 17.3% with its combination. Thus, adding effector CD8^+^ T cells (in a combinatorial setting) increased the killing efficiency compared to CDT alone. Mechanistically, this enhancement indicates that SHINE functions as an immunostimulatory chemodynamic nanovaccine *in vitro*. Beyond redox-mediated cytotoxicity, it recruits and empowers adaptive immunity to produce a substantially deeper suppression of tumor cell viability. The resulting DC–T cell crosstalk increased CD69/CD25 expression and elevated GZB/perforin, yielding bystander cytotoxicity that surpassed direct CDT alone.

### 2.10 Tumor targeting, biodistribution, and pharmacokinetics of SHINE nanovaccine in 4T1-bearing mice

**Fig. 4:**
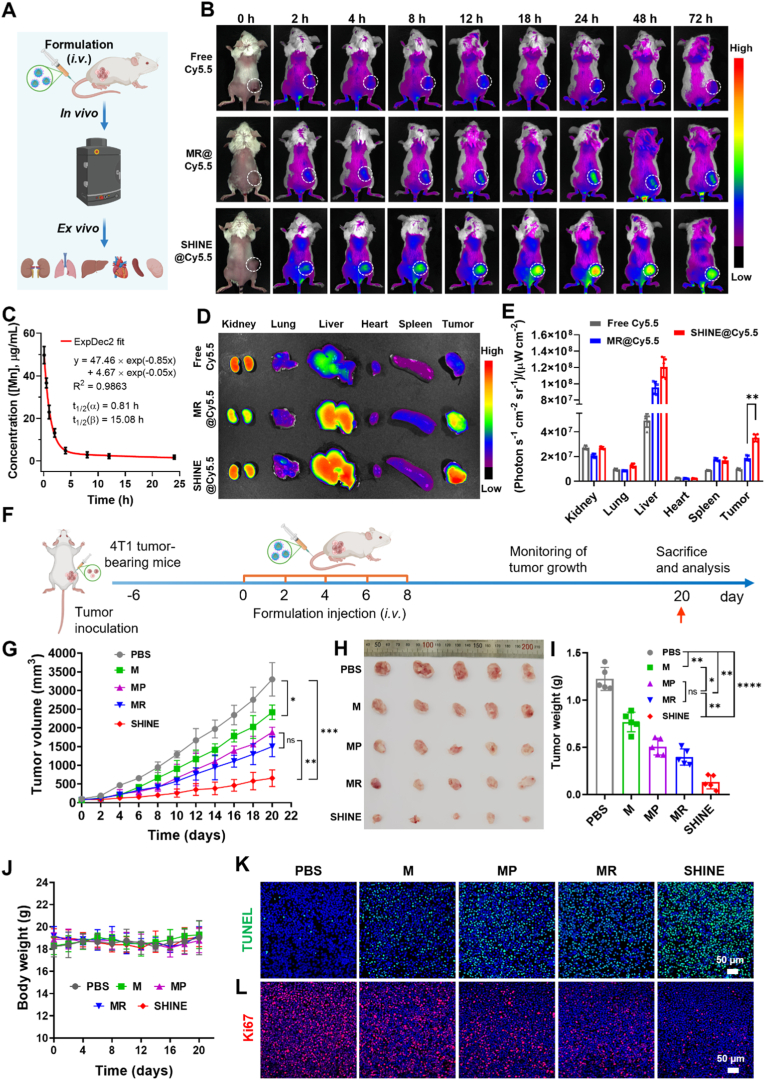
***In vivo* biodistribution, pharmacokinetics, and anti-tumor efficacy of the SHINE nanovaccine in 4T1 tumor-bearing mice.** (**A**) Schematic illustration of the *in vivo* and *ex vivo* fluorescence imaging workflow following intravenous administration of Cy5.5-labeled formulations. (**B**) *In vivo* fluorescence images acquired at different time points up to 72 h post-injection, showing prolonged tumor accumulation of SHINE relative to the control formulations. (**C**) Pharmacokinetic profiles of SHINE intravenously injected into the 4T1 tumor-bearing mice. (**D, E**) *Ex vivo* fluorescence images and quantitative analysis of major organs and tumors collected at 72 h post-injection, confirming enhanced tumor accumulation of SHINE. (**F**) Schematic illustration of the treatment schedule used for therapeutic evaluation in 4T1 tumor-bearing mice. (**G**) Tumor growth curves during treatment. (**H**) Photographs of excised tumors and (**I**) final tumor weights, showing the strongest tumor suppression in the SHINE-treated group. (**J**) Body weight changes during treatment, indicating no obvious systemic toxicity under the tested conditions. (**K**) TUNEL staining and (**L**) Ki67 immunofluorescence staining of tumor sections, demonstrating increased apoptosis and reduced proliferative activity in the SHINE group. M, MnO_2_@PEG; MR, MnO_2_@R848; MP, MnO_2_@aPD-L1; SHINE, MnO_2_@R848@aPD-L1. Data are presented as mean ± SD (*n = 5*). ∗*p*< 0.05, ∗∗*p*< 0.01, ∗∗∗*p*< 0.001, ∗∗∗∗*p* < 0.0001; n.s., not significant (*p* > 0.05). Panel A created with BioRender.com.

To determine how the formulation design governs the *in vivo* fate, we performed whole-body and *ex vivo* IVIS imaging of Cy5.5-labeled nanoparticles following intravenous injection (Fig. 4A). *In vivo* imaging showed systemic distribution for all MnO_2_-based formulations, followed by progressive washout from non-target tissues (Fig. 4B). The MR@Cy5.5 treatment group exhibited modest tumor accumulation, which peaked transiently and subsequently declined, consistent with passive enhanced permeability and retention (EPR) capture followed by clearance. In contrast, aPD-L1-decorated SHINE@Cy5.5 displayed intense and persistent tumor fluorescence that remained bright for 72 h, indicating active PD-L1 targeting and prolonged tumor retention. The superiority of SHINE@Cy5.5 over MR@Cy5.5 suggests that PD-L1 engagement drives the major gain in tumor localization, indicating receptor-mediated targeting rather than relying solely on the EPR effect. Accordingly, conjugation of aPD-L1 to the SHINE surface facilitated preferential accumulation in PD-L1^+^ tumors, thereby enhancing tumor selectivity and cellular uptake. Importantly, this enhancement persisted at the final time point, emphasizing retention.

To further define the *in vivo* behavior of SHINE after intravenous administration, blood pharmacokinetics were evaluated by quantifying Mn as a surrogate marker of SHINE systemic exposure using ICP-OES (Fig. 4C and Table S1). The blood concentration-time profile was best fitted by a two-compartment model, revealing a rapid distribution phase and a slower elimination phase, with t_1/2_(α) = 0.81 h and t_1/2_(β) = 15.08 h. These data indicate that SHINE underwent rapid initial redistribution after injection while maintaining prolonged Mn-associated systemic exposure over time. Together with the sustained tumor-localized Cy5.5 signal, these pharmacokinetic results support a sufficiently broad circulation and tumor-accessible time window for effective tumor targeting and retention.

*Ex vivo* organ imaging at 72 h (Fig. 4D) and semi-quantitative analysis (Fig. 4E) corroborated these trends. Tumor radiant efficiency for SHINE@Cy5.5, MR@Cy5.5, and free Cy5.5 was 3.5 × 10^7^, 1.87 × 10^7^, and 0.96 × 10^7^ (photons s^−1^ cm^−2^ sr^−1^)/(μW cm^−2^)), respectively. Thus, SHINE@Cy5.5 achieved 1.9-fold higher tumor accumulation than MR@Cy5.5 (*p* < 0.01) and 3.6-fold higher accumulation than free Cy5.5 (*p* < 0.001). As expected for nanoscale systems, uptake by the mononuclear phagocyte system was evident, with the liver exhibiting prominent signals across all nanoparticle groups, primarily because of Kupffer cells. The kidneys showed a moderate signal, whereas those of the lungs and heart remained low, suggesting minimal acute pulmonary trapping and cardiotoxic exposure. Collectively, these results demonstrate that antibody-mediated active targeting enhances the *in vivo* tumor localization and retention of the SHINE nanovaccine. The sustained intratumoral signal of SHINE@Cy5.5, together with the prolonged blood pharmacokinetic profile of SHINE, supports a favorable *in vivo* delivery window for subsequent therapeutic activity.

### 2.11 Anti-tumor efficacy of SHINE in 4T1 tumor-bearing mice

Encouraged by its potent *in vitro* cytotoxicity against tumor cells and excellent tumor targeting, we evaluated the *in vivo* antitumor activity of the SHINE nanovaccine in a 4T1 tumor-bearing mouse model (Fig. 4F). Mice received an optimal SHINE dose of 100 mg kg^−1^ containing 5 mg kg^−1^ Mn, 2.2 mg kg^−1^ R848, and 1.0 mg kg^−1^ aPD-L1, which was determined from preliminary studies (Fig. S16).

Relative to rapid tumor growth in the PBS group (3300.8 mm^3^ by day 20), the M group (CDT only) produced a modest delay (2419.6 mm^3^, 1.4-fold lower, *p* < 0.05), indicating limited efficacy for CDT monotherapy (Fig. 4G and H). In contrast, MP (CDT + checkpoint engagement) and MR (CDT + adjuvant delivery) groups showed enhanced tumor inhibition, yielded final volumes of 1885.9 mm^3^ and 1503.1 mm^3^, respectively, which were 1.8-fold (*p* < 0.01) and 2.2-fold (*p*< 0.01) lower compared with the PBS group. Remarkably, the complete SHINE construct (CDT + R848 + aPD-L1) slowed tumor growth. The tumor size in the SHINE group significantly decreased to 654.4 mm^3^, which was 3.7-fold (*p* < 0.001), 2.9-fold (*p* < 0.01), and 2.3-fold (*p* < 0.01) lower than that in the M, MP, and MR groups, respectively. Moreover, to emphasize the cooperative interaction among CDT, aPD-L1, and R848 in SHINE, the expected additive effect and the synergy ratio (R) were calculated using the fractional tumor volume method (Table S2). Accordingly, R = 1.31 > 1, indicating a synergistic *in vivo* effect for SHINE rather than a purely additive combination. Thus, co-delivering R848 and aPD-L1 on the GSH-responsive MnO_2_ NPs yields a supra-additive reduction in tumor growth. Consistent with the tumor growth curves, the terminal tumor mass was ∼150 mg in the SHINE group, which was significantly lower than that in all comparators, representing 9.3-fold (*p* < 0.0001), 5.8-fold (*p* < 0.001), 3.8-fold (*p* < 0.01), and 2.9-fold (*p* < 0.01) lower levels than those in the PBS, M, MR, and MP groups, respectively (Fig. 4I).

To assess *in situ* apoptosis, we conducted terminal deoxynucleotidyl transferase-mediated dUTP nick-end labeling (TUNEL) on tumor sections (Fig. 4K). PBS-treated tumors showed a scarcity of TUNEL-positive nuclei, whereas M tumors showed a modest increase. The incorporation of either R848 (MR) or aPD-L1 (MP) further increased apoptotic labeling. Notably, the SHINE group exhibited the most extensive TUNEL signal across the tumor parenchyma, indicating robust therapy-induced DNA fragmentation. Proliferation was evaluated by Ki-67 immunofluorescence (Fig. 4L). Ki-67 expression was the highest in the PBS group and decreased sequentially in the M, MR, and MP groups, with the lowest expression in the SHINE group, confirming the effective suppression of cell proliferation by the combined treatment. These results are consistent with the therapeutic outcomes, specifically the reduction in tumor volume and weight, as discussed above.

Together, these findings indicate that the SHINE nanovaccine exhibited potent antitumor activity in 4T1 tumor-bearing mice. The pronounced therapeutic advantage of SHINE underscores the importance of its rational design, integrating GSH-responsive CDT, R848-mediated immune stimulation, and PD-L1 blockade, for effective CDT–immunotherapy synergy. Notably, the synergistic antitumor efficacy observed with the combination of CDT and immunotherapy far exceeds that of CDT alone, which is insufficient to induce robust tumor cell killing.

Furthermore, we systematically assessed the biosafety of the SHINE nanovaccines. In the red blood cell hemolysis assay, SHINE caused minimal hemolysis, which was well below the commonly accepted 5% threshold [81] for hemocompatibility across the tested concentration range (Fig. S17). Body weight remained stable in all treatment groups throughout the study, with no significant loss relative to controls (Fig. 4J). Serum biochemistry showed that liver function markers (AST, ALT, ALP, and T-Bil) and kidney function markers (BUN, CREA, and UA) in the SHINE group were comparable to those in PBS-treated controls, and all values remained within the physiological range reported for female BALB/c mice, indicating no apparent systemic toxicity after 20 days of treatment (Fig. S18). In parallel, H&E staining of liver, heart, lungs, spleen, and kidneys revealed preserved tissue architecture, with no evidence of acute injury or inflammatory/degenerative changes (Fig. S19). Taken together, these results indicate that SHINE exhibits favorable biocompatibility and systemic safety at the therapeutic dosing used *in vivo*, supporting the translational potential of this GSH-responsive, MnO_2_-based nanovaccine for CDT–immunotherapy.

### 2.12 SHINE nanovaccine triggers robust immune responses in 4T1 tumor–bearing mice

**Fig. 5:**
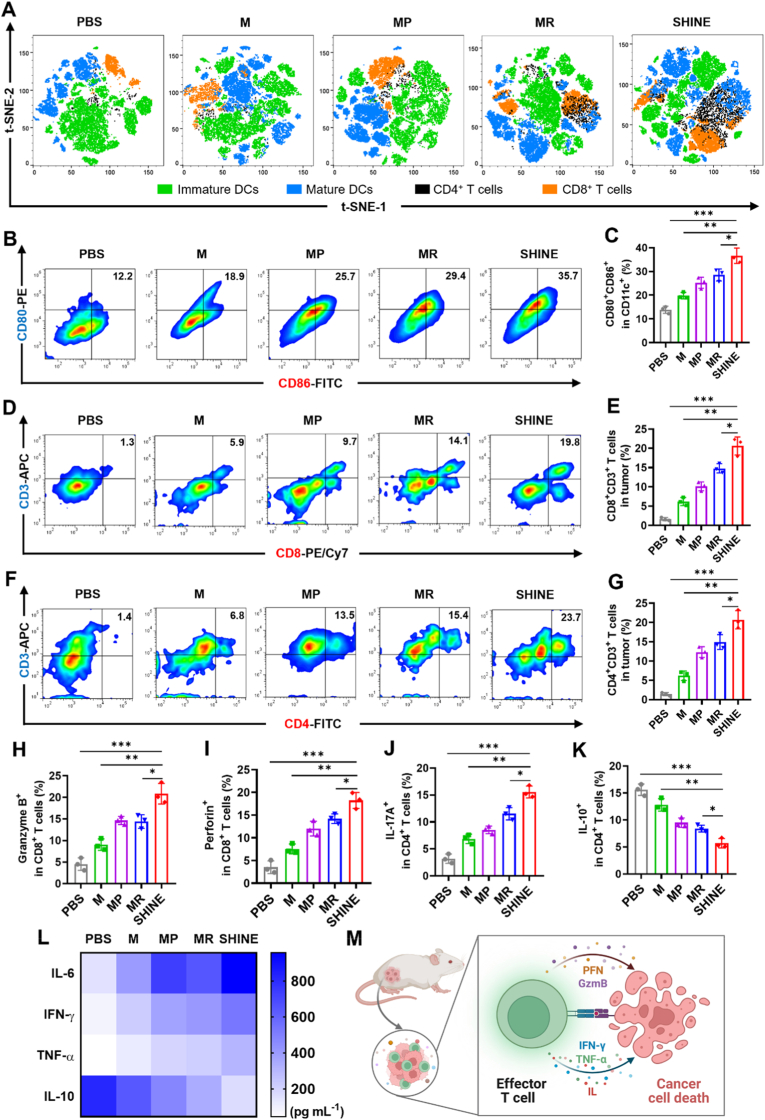
***In vivo* immunomodulation induced by the SHINE nanovaccine in 4T1 tumor-bearing mice**. (**A**) Representative t-SNE maps of tumor-infiltrating immune cell populations after the indicated treatments, generated using FlowJo v10 software, showing treatment-dependent remodeling of the intratumoral immune landscape. (**B, C**) Representative flow cytometric plots and quantitative analysis of mature DCs (CD11c^+^CD80^+^CD86^+^) in lymph nodes, demonstrating enhanced DC maturation in the SHINE group. (**D, E**) Representative flow cytometric plots and quantification of tumor-infiltrating CD8^+^ T cells after different treatments. (**F, G**) Representative flow cytometric plots and quantification of tumor-infiltrating CD4^+^ T cells after different treatments. (**H, I**) Flow cytometric quantification of granzyme B and perforin expression in CD8^+^ T cells, indicating enhanced cytotoxic effector function. (**J, K**) Flow cytometric quantification of IL-17A and IL-10 expression in CD4^+^ T cells, showing increased pro-inflammatory cytokine production and reduced immunosuppressive signaling after SHINE treatment. (**L**) ELISA analysis of serum cytokines (IL-6, IFN-γ, TNF-α, and IL-10) on day 20 post-treatment, indicating systemic immune activation. (**M**) Schematic illustration summarizing cytokine release from activated T cells and the resulting antitumor immune response. M, MnO_2_@PEG; MR, MnO_2_@R848; MP, MnO_2_@aPD-L1; SHINE, MnO_2_@R848@aPD-L1. Data are presented as the mean ± SD (*n = 3*). ∗*p*< 0.05, ∗∗*p*< 0.01, ∗∗∗*p* < 0.001. Panel M created with BioRender.com.

Motivated by its potent antitumor efficacy, we investigated the immune response elicited by the SHINE nanovaccine. To assess how SHINE modulates the intratumoral immune landscape, we performed t-distributed stochastic neighbor embedding (t-SNE) analysis following gating of DC and T cell subsets (gating strategy shown in Fig. S20). Across all treatment groups, four major immune populations were identified: immature DCs (iDCs), mature DCs (mDCs), helper T lymphocytes (CD4^+^), and cytotoxic T lymphocytes (CTLs, CD8^+^) (Fig. 5A). In PBS-treated controls, the immune landscape was dominated by iDCs with only sparse CTL infiltration, consistent with an immunologically “cold” TME. Treatments with M, MR, or MP resulted in moderate expansion of mDCs and CTLs. In contrast, the SHINE nanovaccine induced a dramatic remodeling of the immune topology: extensive, contiguous mDC clusters emerged, accompanied by robust infiltration of both CTLs and helper T cells, whereas the iDC compartment was visibly diminished. Importantly, these immune subsets displayed spatial interdigitation, a pattern indicative of effective antigen presentation and T cell priming. Collectively, the t-SNE topography suggests that SHINE reprograms the tumor from an iDC-rich, T cell-depleted state into a DC-mature, T cell-engaged niche conducive to productive antitumor immunity.

DCs are pivotal antigen-presenting cells (APCs) that are essential for initiating T cell activation and orchestrating effective antitumor immune responses [82,83]. Therefore, DC maturation was quantified in tumor-draining lymph nodes (TDLNs) by flow cytometry using the CD11c^+^CD80^+^CD86^+^ gate (Figure S21 and Fig. 5B and C). The M-treated group showed increased maturation to 18.1% (1.5-fold *vs.* PBS, *p* < 0.05), consistent with the CDT-induced ICD release of DAMPs for antigen uptake. Incorporation of the PD-L1–targeting (MP) or TLR7/8 agonist (MR) further elevated maturation to 26.2% (2.2-fold *vs*. PBS, *p* < 0.01) and 29.1% (2.4-fold *vs.* PBS, *p* < 0.01), respectively, indicating that either the adjuvancy or enhanced tumor engagement augments priming cues. Notably, the SHINE-treated group yielded the highest fraction of mature DCs (36.5%), which was 1.3-, 1.4-, 2.0-, and 3.0-fold greater than those in the MR, MP, M, and PBS groups, respectively. Taken together, SHINE transforms the TDLNs into an immunogenic hub with significantly expanded pools of mature DCs poised for cross-presentation.

We assessed whether enhanced priming translated into effector recruitment by enumerating the tumor-infiltrating lymphocytes. Both cytotoxic T lymphocytes (CTLs) (CD3^+^CD8^+^ T cells) and helper T cells (CD3^+^CD4^+^ T cells) are key contributors to adaptive immune regulation. Flow cytometry analysis demonstrated a marked elevation in both CD8^+^ and CD4^+^ T cell populations in tumors from SHINE-treated mice compared with all other groups (gating strategy shown in Fig. S22). As shown in Fig. 5D and E, the proportion of intratumoral CD8^+^ T cells reached 20.1% in the SHINE-treated group, which was 11.2-, 3.8-, 1.9-, and 1.4-fold higher than those in the PBS (1.8%), M (5.3%), MP (10.8%), and MR (13.9%) groups, respectively. A similar hierarchy was observed for CD3^+^CD4^+^ helper T cells (Fig. 5F and G), where SHINE markedly increased the proportion to 23.0%, corresponding to 15.3-, 3.6-, 1.8-, and 1.6-fold higher relative to PBS (1.5%), M (6.4%), MP (12.7%), and MR (14.6%), respectively. These findings suggest that SHINE-induced synergistic treatment enhances the immunogenicity of TME, facilitates T cell activation, and consequently amplifies antitumor efficacy.

Cytokines play a vital role in modulating anti-tumor responses [84]. Therefore, we measured cytokine levels in the TME and serum. Flow cytometric analysis revealed a marked increase in GZB-producing CD8^+^ T cells in SHINE-treated tumors (20.2%) compared to the control group (4.9%), whereas MP (15.3%) and MR (14.5%) showed only a moderate GZB level (Figures S23A, B and Fig. 5H). Consistently, SHINE-treated increased the number of perforin-producing CD8^+^ T cells by 1.3-, 1.4-, and 4.1-fold compared to that in the MP, MR, and PBS groups, respectively (Figure S23A, C and Fig. 5I). Next, we examined helper T cell cytokine levels (Fig. S24A). As shown in Figure S24B and Fig. 5J, the proportion of IL-17A^+^CD4^+^ T cells increased from 4.3% (PBS) to 7.2% with M (1.7-fold, *p* < 0.05), 8.9% with MP (2.0-fold, *p* < 0.01), and 12.3% with MR (2.9-fold, *p* < 0.01), peaking at 17.3% with SHINE (4.0-, 2.4-, 1.9-, and 1.4-fold *vs.* PBS, M, MP, and MR, respectively). In contrast, IL-10^+^CD4^+^ T cells were the lowest in SHINE-treated groups (6.1%), representing 1.3-, 1.4-, 1.9-, and 3.2-fold reductions relative to MR, MP, M, and PBS, respectively (Figure S24C and Fig. 5K). Serum secretions were analyzed using ELISA. As shown in Fig. 5L, SHINE induced the highest systemic levels of pro-inflammatory cytokines (IFN-γ, TNF-α, and IL-6) while significantly reducing anti-inflammatory cytokines (IL-10). Overall, the cytokine analysis results showed that the SHINE nanovaccine reprogrammed both the TME and the systemic milieu to relieve tumor-driven immunosuppression and reinforce anti-tumor immunity.

To determine whether these immune changes translated into functional antitumor cytotoxicity, we performed an *in vitro* tumor cell-killing assay using CD8^+^ T cells isolated from the tumors of 4T1 tumor-bearing mice treated with SHINE. These effector cells were co-incubated with 4T1 tumor cells or 3T3 normal fibroblasts at different effector-to-target (E:T) ratios. As shown in Fig. S25, SHINE-primed CD8^+^ T cells induced a clear E:T ratio-dependent reduction in 4T1 cell viability, whereas only minimal effects were observed against 3T3 cells. At E:T ratios of 1:1, 5:1, and 10:1, 4T1 viability decreased to approximately 75%, 45%, and 33%, respectively, while 3T3 viability remained relatively high at about 95%, 92%, and 84%. These data demonstrate that SHINE elicits functionally active cytotoxic T cells with preferential tumor-directed killing activity, further confirming the functional competence of the induced antitumor immune response.

### 2.13 Multimodal transcriptomic reprogramming of tumors by SHINE nanovaccine

#### 2.13.1 SHINE treatment globally reprograms tumor transcriptomes

**Fig. 6:**
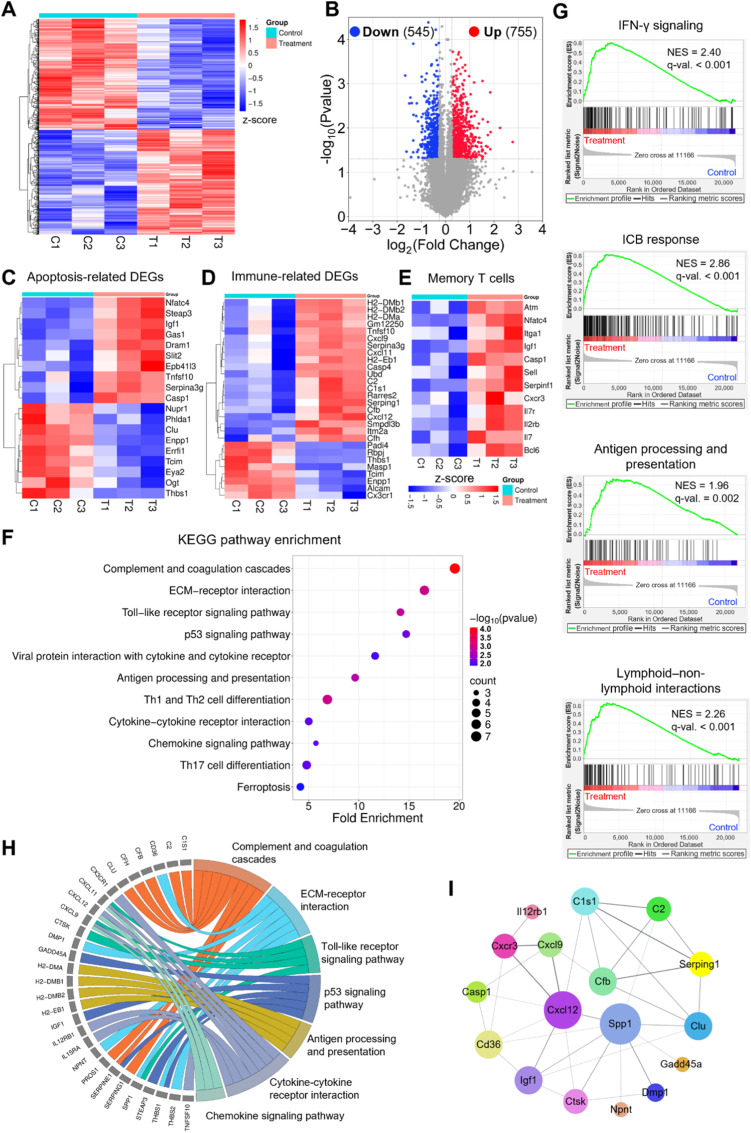
**Transcriptomic analysis of treatment-induced molecular changes in tumor tissues**. (**A**) Heatmap and (**B**) volcano plot showing differentially expressed genes (DEGs) between control (C1–C3) and treatment (T1–T3) tumor samples (|log_2_ fold change| > 1.2, *p* < 0.05), demonstrating broad transcriptional remodeling after treatment. (**C–E**) Heatmaps of representative DEGs associated with the apoptotic process, immune activation, and memory T cells, respectively, highlighting treatment-associated expression patterns related to tumor cell death and antitumor immunity. (**F**) Kyoto Encyclopedia of Genes and Genomes (KEGG) pathway enrichment analysis of DEGs shown as a dot plot of the top enriched pathways ranked by fold enrichment and −log_10_(p-value). (**G**) Gene Set Enrichment Analysis (GSEA) plots showing significant enrichment of IFN-γ signaling, ICB response, antigen processing and presentation, and lymphoid–non-lymphoid interactions in treated tumors, supporting activation of antitumor immune programs. NES: normalized enrichment score. (**H**) Pathway crosstalk analysis illustrating shared genes among enriched pathways. (**I**) Protein–protein interaction (PPI) network analysis highlighting hub genes within the enriched pathways.

To elucidate the antitumor mechanisms of the SHINE nanovaccine, we performed whole-transcriptome profiling of tumor tissues 20 days post-injection using RNA sequencing (RNA-seq). The heat map (Fig. 6A) shows a clear separation between the control (C1–C3) and SHINE-treated (T1–T3) groups, revealing a global transcriptional shift induced by the nanovaccine. SHINE-treated tumors exhibited a consistent upregulation of genes that were suppressed in the control group, indicating a coherent and robust transcriptional response. This was further supported by the volcano plot in Fig. 6B, which identified 1300 differentially expressed genes (DEGs) using a threshold of |log_2_FC| > 1.2 and *p* < 0.05. Of these, 755 genes were upregulated and 545 were downregulated in the treatment group. Taken together, these data show that SHINE triggers broad remodeling rather than isolated gene changes.

#### 2.13.2 SHINE treatment enhances apoptosis and immune activation programs

We examined DEGs associated with apoptosis and immune activation. Fig. 6C shows the upregulation of apoptotic genes involved in both the intrinsic and extrinsic cell death pathways, including *Casp1, Tnfsf10, Dram1, Gas1*, and *Serpina3g*. In contrast, pro-survival and growth-related genes such as *Clu, Enpp1, Errfi1, Ogt,* and *Thbs1* were downregulated. These shifts suggested enhanced programmed cell death upon SHINE treatment. Fig. 6D highlights the key immune-stimulatory DEGs. Notably, MHC class II antigen-presentation genes (*H2-DMb1, H2-DMb2*, and *H2-Eb1*) were strongly upregulated, suggesting enhanced peptide presentation by professional APCs. Upregulation of IFN-γ–inducible chemokines (*Cxcl9, Cxcl11*) supports increased Th1 and CTL recruitment. Complement/innate components (*C1s1, Serping1*) and ubiquitin-proteasome/processing elements (*Ubd*) tended to increase, suggesting heightened innate immunity and antigen turnover. These findings suggest that SHINE promotes tumor cell death and reshapes the TME into an “immune-hot” state, characterized by interferon responses, antigen presentation, and lymphocyte-recruiting chemokines that drive increased mature DCs and intratumoral CD8^+^/CD4^+^ infiltration.

#### 2.13.3 SHINE treatment enriches innate and adaptive immune pathways

To gain deeper insights into the functional pathways modulated by SHINE, pathway enrichment analysis using the Kyoto Encyclopedia of Genes and Genomes (KEGG) database was conducted on the DEGs and visualized in a bubble plot (Fig. 6F). Immune-related pathways, including complement and coagulation cascades, ECM–receptor interaction, Toll-like receptor signaling, antigen processing and presentation, cytokine–cytokine receptor interaction, and chemokine signaling, were significantly enriched. Complement and coagulation cascades showed the strongest enrichment (fold enrichment ∼19; –log_10_p ∼4), suggesting activation of soluble innate effectors that can opsonize dying cells and enhance antigen uptake by professional APCs. ECM–receptor interaction also exhibited high enrichment (∼16-fold), reflecting the remodeling of matrix/adhesive transcripts that may facilitate immune infiltration. Toll-like receptor signaling pathway activation, likely because of R848-mediated TLR7/8 engagement and DAMPs release, further supports immune reprogramming. Enrichment in cytokine/chemokine pathways reflects the enhanced intercellular immune communication required for T cell recruitment and maturation, aligning with the T cell infiltration observed *in vivo*. Apoptosis-related p53 signaling was also upregulated, which is consistent with the DNA damage and oxidative stress responses under CDT. Overall, the KEGG analysis reinforced that SHINE elicits multifaceted responses, simultaneously activating immune pathways that convert a poorly immunogenic tumor into an immune-reactive milieu poised for antigen capture, presentation, and apoptotic pathways while modulating the tumor stroma and suppressive niches.

To examine pathway-level coordination beyond DEGs, gene set enrichment analysis (GSEA) was conducted (Fig. 6G). All identified pathways displayed strong positive enrichment and a clear shift toward SHINE-treated samples, with core enrichment genes concentrated at the top of the ranked gene list. Specifically, the strong upregulation of IFN-γ signaling (NES = 2.40, q < 0.001) suggests enhanced MHC expression and chemokine gradients that promote the infiltration of cytotoxic T cells. Notably, strong enrichment of ICB signatures (NES = 2.86, q < 0.001) indicates that SHINE reprogrammed tumors into a PD-1/PD-L1–responsive state, consistent with its PD-L1-targeting component and supporting potential synergy with checkpoint immunotherapy. The antigen processing and presentation pathway (NES = 1.96, q = 0.002) was also enriched, reflecting coordinated upregulation of the proteasome–transporter associated with antigen processing–MHC-I axis and the MHC-II invariant chain pathway. This indicates enhanced cross-presentation capacity within the TME, consistent with CDT-triggered ICD providing tumor antigens and R848 licensing DCs for efficient antigen display and T cell priming. In addition, lymphoid–non-lymphoid cell interactions were positively enriched (NES = 2.26, q < 0.001), suggesting enhanced adhesive and chemotactic crosstalk between T/B/NK cells and stromal cells. Together, these findings demonstrate that SHINE activates transcriptional programs that support antigen release, cross-presentation, chemokine-driven immune infiltration, and checkpoint sensitization, which are hallmarks of effective cancer immunotherapy. This immune remodeling provides a mechanistic basis for strong DC maturation, T cell infiltration, and antitumor efficacy observed *in vivo*.

#### 2.13.4 SHINE treatment engages crosstalk hubs for coordinated tumor reprogramming

A chord diagram was generated to assess the pathway interconnectivity (Fig. 6H), linking key DEGs to multiple enriched pathways. The visualization highlights key genes, such as *Spp1* and *Cxcl12*, as multi-pathway nodes. This modular overlap suggests that SHINE coordinates complex transcriptional networks rather than triggering isolated pathways, which is consistent with the ICD-driven vaccine effects. To further investigate the functional interactions of these DEGs in immunological processes, STRING, a database that integrates all known and predicted protein–protein interactions (PPIs), was utilized to map functional gene interaction networks after SHINE treatment (Fig. 6I). In the STRING-derived PPI, the larger the number of circular nodes, the greater the centrality in the network. *Spp1* and *Cxcl12* showed the highest degree and emerged as central hubs, interfacing with other modular cluster complements (*C1s1, C2, Cfb, Serping1*, and *Clu*), chemokines (*Cxcl9* and *Cxcr3*), cell-death/stress (*Casp1* and *Gadd45a*), and stromal/adhesion genes (*Cd36, Npnt*, and *Dmp1*). Mechanistically, *Spp1* (osteopontin) is a matricellular ligand for integrins and CD44 [85,86]. As a network hub, its connections to *Cd36/Npnt* and chemokine/complement modules suggest that osteopontin remodels adhesive niches and reinforces immune–stromal contacts, thereby supporting stable APC–T cell interactions within inflamed tumors. *Cxcl12*, a chemokine critical for leukocyte positioning [[87], [88], [89]], bridges IFN-γ–inducible axes (*Cxcl9, Cxcr3*) with innate complement modules (*Cfb, C2*), enabling coordinated recruitment/positioning of DCs and CTLs. These hub-centric patterns suggest that SHINE activates coordinated immune circuits rather than isolated responses, ensuring effective DC–T cell interactions and synergy with PD-L1 blockade, thereby promoting T cell infiltration.

Collectively, the transcriptomic data validated that SHINE induced multi-pathway reprogramming of the TME. Through the coordinated activation of apoptotic stress, innate immunity, adaptive immunity, and matrix remodeling, SHINE promotes broad transcriptional reprogramming that reshapes the immunosuppressive TME and initiates antitumor immune responses. This mechanistic insight provided a molecular rationale for the robust therapeutic effects observed *in vivo* with SHINE.

### 2.14 Anti-metastatic effects of the SHINE nanovaccine

**Fig. 7:**
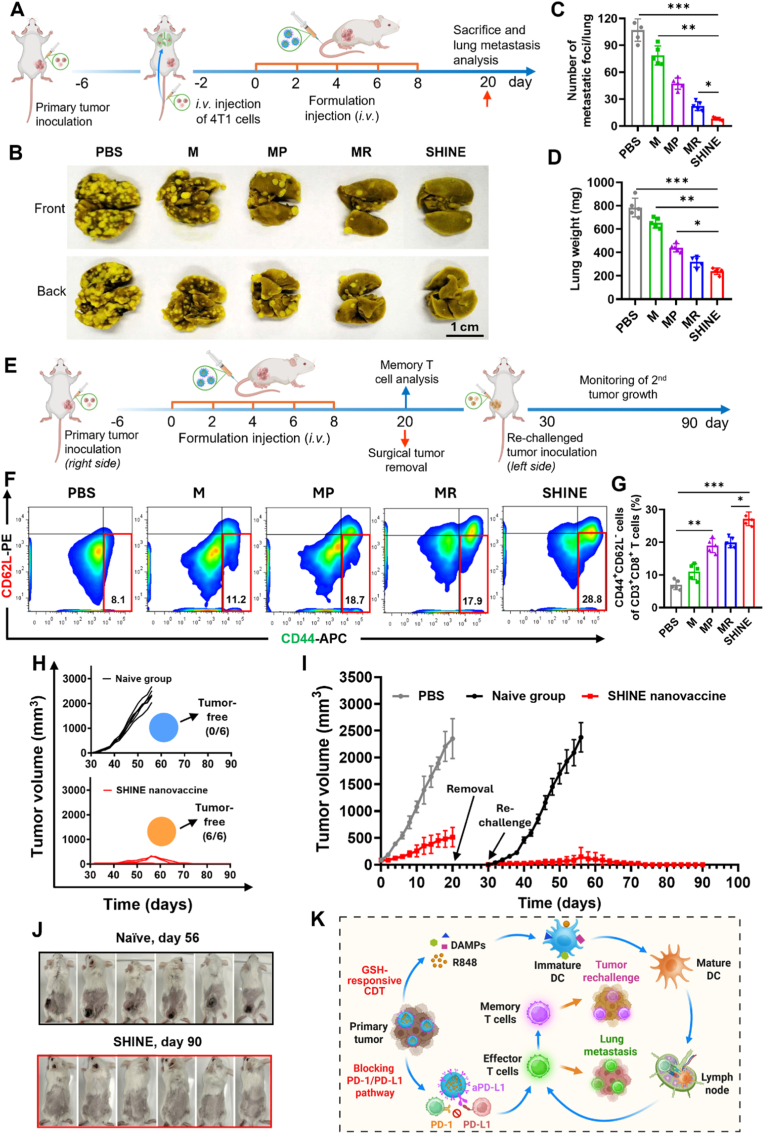
**Anti-metastatic and long-term immune memory effects of the SHINE nanovaccine.** (**A**) Schematic illustration of the experimental design for establishing the 4T1 lung metastasis model and the treatment schedule. (**B**) Representative photographs of Bouin's solution-stained lungs collected from different treatment groups. (**C, D**) Quantification of the number of metastatic lung nodules and lung weights, demonstrating that SHINE most effectively inhibited lung metastasis. (**E**) Schematic illustration of the experimental design used to evaluate immunological memory and long-term protection in the 4T1 tumor rechallenge model. (**F, G**) Representative flow cytometric plots and quantitative analysis of effector memory T cells (T_EM_, CD3^+^CD8^+^CD44^+^CD62L^−^) in the spleen, showing enhanced memory T cell formation after SHINE treatment. (**H**) Individual tumor growth curves in naïve and SHINE-treated mice after tumor rechallenge. (**I**) Tumor growth curves of the naïve, PBS control, and SHINE groups, showing that SHINE conferred protection against secondary tumor challenge. (**J**) Photographs of mice in the naïve group (day 56) and SHINE group (day 90), further illustrating the long-term protective effect of SHINE. (**K**) Schematic illustration of the proposed mechanism by which SHINE induces antitumor immune responses, suppresses lung metastasis, and establishes durable immune memory. M, MnO_2_@PEG; MR, MnO_2_@R848; MP, MnO_2_@aPD-L1; SHINE, MnO_2_@R848@aPD-L1. ∗*p*< 0.05, ∗∗*p*< 0.01, ∗∗∗*p* < 0.001. Panel K created with BioRender.com.

Inspired by the high efficacy of the SHINE nanovaccine in inhibiting primary tumors, we explored its ability to suppress invasive lung metastasis, which is a major challenge for current cancer treatments. A lung metastatic tumor model was established by intravenously injecting 4T1 cells into mice in which the primary tumor had been engrafted 4 days earlier (Fig. 7A). This model can simulate hematogenous metastasis as an artificial whole-body model of spreading tumors [90]. On day 20, the lungs were harvested to analyze metastatic levels. Macroscopic images showed an extensive spread of metastatic foci in the PBS group, fewer nodules after CDT alone (M), and further reduction with either the R848 adjuvancy (MR) or PD-L1 targeting (MP). More importantly, metastatic foci in the lungs were nearly eradicated in the SHINE-treated group, highlighting the potent anti-metastatic activity of the combined CDT and immunotherapy approach (Fig. 7B). The average number of pulmonary nodules decreased from 104.5 per lung in PBS to 72.8 in M (1.44-fold reduction, *p* < 0.05), 46.7 in MP (2.24-fold reduction, *p* < 0.01), and 27.4 in MR (3.81-fold reduction, *p* < 0.001). SHINE treatment dramatically reduced the cell count to 9.6 nodules, representing 10.88-, 7.58-, 4.86-, and 2.85-fold fewer nodules than with PBS, M, MP, and MR, respectively (Fig. 7C). In addition, the lung weights paralleled these trends, with the SHINE-treated group showing the lowest mass among all groups (Fig. 7D). In summary, these findings indicate that the combination formulation (SHINE) outperformed all single-component controls by a substantial margin in both nodule count and lung weight, confirming that SHINE is effective in inhibiting tumor metastasis to the lungs.

### 2.15 SHINE nanovaccine elicits durable tumor-specific immune memory in a 4T1 rechallenge model

Building on the high therapeutic efficacy of SHINE in inhibiting both primary tumors and lung metastases, we investigated whether the SHINE-induced immune response was tumor-specific and capable of generating long-term immune memory to protect against tumor rechallenge. To establish a rechallenge model, BALB/c mice bearing right flank 4T1 primary tumors were administered various formulations and subjected to complete surgical tumor resection on day 20. On day 30, the SHINE-treated mice were subcutaneously rechallenged with 4T1 cells in the contralateral (left) flank. Simultaneously, age- and sex-matched naïve mice received an equal number of 4T1 cells as controls for tumor growth (Fig. 7E).

Given that effector T cells can differentiate into effector memory T (T_EM_) cells, which play a pivotal role in long-term immune protection [24,68,91,92], we analyzed splenic T_EM_ (CD3^+^CD8^+^CD44^+^CD62L^−^) by flow cytometry before rechallenge (Fig. S26). As shown in Fig. 7F and G, the frequency of T_EM_ cells increased across treatment groups. The mice treated with MnO_2_ alone (M) exhibited a low T_EM_ proportion (11.9%), whereas the incorporation of R848 (MR) and aPD-L1 (MP) moderately elevated the T_EM_ frequencies to 19.2% and 17.3%, respectively. Notably, the SHINE-treated group achieved the highest T_EM_ proportion of 28.1%, representing a 3.47- and 1.46-fold increase relative to the PBS and MR groups, respectively. In parallel, transcriptomic analysis revealed elevated expression of memory T cell–related genes, including *Atm, Nfatc4, Sell, Il7r,* and *Cxcr3*, in tumors from SHINE-treated mice (Fig. 6E). These phenotypic and molecular findings suggest that the SHINE nanovaccine induces a potent immune memory that can effectively sustain antitumor protection.

To determine whether this enhanced immune memory conferred functional protection, we monitored tumor growth following rechallenge (Fig. 7H–J). In the naïve control group, tumors formed in all mice (6/6) and expanded rapidly to an average volume of ∼2347 mm^3^ by day 56 (*i.e*., day 26 post-rechallenge). In contrast, SHINE-treated mice exhibited significant tumor protection, with only two of six mice initially developing small tumors (average volume of ∼236 mm^3^ by day 56), which subsequently regressed and fully disappeared by day 67. Remarkably, the remaining four SHINE-vaccinated mice remained tumor-free even on day 90 (*i.e*., day 60 post-rechallenge). Notably, all SHINE-treated mice survived to day 90, whereas all naïve controls died within 63 days (Fig. S27). Thus, SHINE conferred durable antigen-specific protection against re-exposure.

## 3 Discussion

Despite remarkable advances in cancer immunotherapy, efficacy and clinical translation remain constrained by insufficient tumor immunogenicity and an immunosuppressive TME. CDT alone is often attenuated by endogenous antioxidants, particularly GSH, and insufficient ROS. In parallel, systemic delivery of immune checkpoint inhibitors such as aPD-L1 poses the risk of immune-related adverse events because of nonspecific exposure, and conventional nanocarriers lack precise spatiotemporal control over drug release. Similarly, immune adjuvants such as R848 (TLR7/8 agonist) may provoke systemic inflammation if not spatially gated to tumors [64,69,93]. To address these barriers, we engineered a GSH-responsive *in situ* cancer nanovaccine, SHINE (MnO_2_@R848@aPD-L1), that strategically couples a CDT-induced ICD “initiator” with dual immune “boosters” (R848 and aPD-L1) in a TME-triggered manner. This “one-particle, tri-trigger” design coordinates where CDT occurs, when adjuvancy is released, and how checkpoint inhibition is applied, enabling tumor-selective *in situ* vaccination without exogenous triggers such as light or high systemic doses of immunoadjuvants. Consistent with this integrated mechanism, synergy analysis confirmed that SHINE produced a synergistic antitumor effect rather than a purely additive inhibition of tumor growth. Notably, while conventional GSH-responsive MnO_2_-based nanoplatforms have primarily focused on acute tumor suppression *via* ROS-mediated CDT or short-term immune activation, they have rarely demonstrated the capacity to elicit durable antitumor immunity [52,54,57,58,60,61,[94], [95], [96], [97]] (Table S3). In contrast, SHINE not only induces potent primary tumor regression but also establishes long-lasting immune memory capable of preventing tumor recurrence upon rechallenge, thereby distinguishing it from earlier MnO_2_-based designs. SHINE represents an advance in precision nanomedicine, coupling tumor-selective activation with multimodal immune engagement to achieve potent, durable, and recurrence-free tumor eradication.

SHINE is rationally designed as a TME-responsive *in situ* cancer nanovaccine that couples ICD-driven vaccination with immune boosting. In SHINE, MnO_2_-mediated GSH depletion enhances CDT, thereby amplifying oxidative stress and disrupting intracellular redox homeostasis, ultimately initiating tumor cell death. Although CDT commonly induces apoptosis through ROS-mediated damage, apoptotic death can become immunogenic under conditions of severe oxidative and organelle stress. In this system, the combined effects of antioxidant depletion and Mn^2+^-associated oxidative amplification are expected not only to activate apoptotic pathways but also to provoke the endoplasmic reticulum stress, mitochondrial dysfunction, and danger signaling events required for ICD. ICD is characterized by the spatiotemporally coordinated release of DAMPs, including CRT exposure, ATP secretion, and HMGB1 release, which collectively alert and activate the host immune system [40,43,73]. Specifically, CRT translocation to the tumor cell surface serves as an “eat-me” signal, promoting phagocytosis by immature DCs; extracellular ATP acts as a “find-me” signal, recruiting APCs to stimulate DC maturation; and HMGB1, released from damaged nuclei, binds to TLR4 on DCs, enhancing antigen processing and cross-presentation [39,40]. Together, these signals converge to amplify DCs activation within TDLNs, leading to the expansion of CD69^+^/CD25^+^CD8^+^ T cells and enhancing their cytotoxic readiness through the production of GZB and perforin. By embedding this immunogenic cascade within a single tumor-activated nanoplatform, SHINE converts dying tumor cells into an endogenous source of both antigens and adjuvant cues, avoiding reliance on exogenous tumor antigens, *ex vivo* DC manipulation, or adoptive cell transfer. Moreover, the self-contained and self-triggered design of SHINE distinguishes it from conventional strategies, such as photo/sonodynamic therapy or radiotherapy, which require external stimuli [32,[98], [99], [100]]. SHINE is fully autoactivated in the TME, offering precision immune engagement with minimal systemic toxicity.

Beyond initiating ICD, effective *in situ* vaccination requires remodeling of the local immune landscape from an immunosuppressive state to an immunologically active one. Consistent with this requirement, SHINE increased intratumoral CD11c^+^CD80^+^CD86^+^ mature DCs, supporting enhanced antigen presentation. SHINE-treated tumors subsequently exhibited greater infiltration of effector CD8^+^ cytotoxic and CD4^+^ helper T cells, accompanied by elevated GZB and perforin and increased proinflammatory cytokines, including IFN-γ and TNF-α, with concomitant suppression of IL-10, consistent with a more cytotoxic, immune-active phenotype. Transcriptomic profiling further corroborated these findings, revealing enrichment of antigen processing and presentation, cytokine–cytokine receptor interaction, interferon-γ signaling, ICB response signatures, and lymphoid–non-lymphoid cell interactions pathways. Complement and coagulation cascades were also enriched, consistent with improved antigen handling and cross-presentation. Network analysis further highlighted *Spp1* and *Cxcl12* as hubs linking tissue remodeling with immune recruitment. Collectively, these data support a self-reinforcing loop in which ICD-driven antigen release, DC maturation, effector T cell function, and cytokine signaling cooperate to reprogram the TME from a suppressive, immune-excluded niche into an immune-active landscape.

Although primary tumor inhibition remains the immediate goal of most therapies, the ability to prevent metastasis and induce long-term immune memory defines true therapeutic success in oncology. In our metastatic 4T1 mouse model, SHINE treatment markedly reduced the pulmonary metastatic burden, as evidenced by Bouin's-stained lung imaging and lung weight quantification. This pronounced anti-metastatic efficacy can be attributed to the ability of SHINE to elicit a tumor-primed, systemically active immune response that is not confined to the primary tumor site. CD8^+^ cytotoxic T lymphocytes, once activated within the primary tumor-draining lymph nodes, enter the systemic circulation and maintain surveillance, enabling the recognition and eradication of disseminated tumor cells in distal tissues. Moreover, transcriptomic analysis of SHINE-treated tumors revealed enhanced expression of chemokines and adhesion molecules involved in T cell trafficking and vascular extravasation, facilitating effector infiltration into metastatic foci.

In addition to suppressing primary and metastatic tumors, SHINE elicited a memory-competent adaptive immune response capable of preventing tumor relapse, as evidenced in our rechallenge tumor model. During the convalescent phase following primary tumor clearance, we observed a marked expansion of splenic CD8^+^CD44^+^CD62L^−^ effector memory T cells (T_EM_). Unlike naïve or central memory subsets, T_EM_ cells represent a hallmark feature of adaptive immunity, immunological memory, by retaining immediate cytotoxic potential and rapidly secreting pro-inflammatory cytokines upon re-exposure to previously encountered tumor antigens, thereby enabling swift, antigen-specific responses against re-emerging tumor cells [24,68,92,[101], [102], [103]]. Their localization in peripheral tissues, including the spleen, facilitates systemic immune surveillance and prompts engagement with disseminated malignancies [104]. The elevated T_EM_ frequency in SHINE-treated mice suggests the formation of a robust antigen-specific memory T cell reservoir capable of sustaining long-term tumor control without the need for booster vaccinations or exogenous antigens. Consistently, tumor transcriptomic profiling further indicated upregulation of memory T cell-associated gene programs in SHINE-treated tumors. Functionally, this durable memory translated into substantial resistance to tumor re-establishment upon rechallenge following complete surgical resection of the primary tumor and subsequent contralateral reinoculation with 4T1 cells; six out of six SHINE-vaccinated mice completely rejected the secondary tumors. Collectively, these phenotypic and molecular signatures, including an increased T_EM_ proportion and elevated expression of memory T cell-associated genes, together with functional protection in the rechallenge model (tumor-free survival), demonstrate that the SHINE nanovaccine not only elicits potent tumor-specific immunity but also establishes long-lasting immune memory capable of preventing tumor recurrence, a hallmark of effective cancer vaccination. (Fig. 7K).

Coupled with a favorable biosafety profile, the integrated TME-triggered architecture that unifies an “initiator” module (CDT-induced ICD) with dual “booster” modules (R848 and aPD-L1) on a single GSH-gated chassis offers a practically tractable route for tumor-specific immunotherapy. This design not only suppresses local tumors but also eradicates distant metastases and establishes long-term immunological memory, thereby preventing tumor relapse.

## 4 Conclusions

In summary, we developed a multifunctional, GSH-responsive immunogenic nanovaccine (SHINE) that rationally leverages the TME for *in situ* cancer vaccination. By integrating a MnO_2_-based CDT “initiator” with dual immune-activating “boosters”, R848 and aPD-L1, SHINE induced robust ICD and elicited potent adaptive immunity. Importantly, SHINE elicited systemic antitumor immunity beyond the primary tumor site, markedly suppressing lung metastasis while establishing durable immunological memory, as evidenced by an increased effector memory T cell fraction, upregulated memory T cell-associated gene expression, and tumor rejection in the rechallenge model. The favorable biosafety profile and compact, nanoadjuvant-loaded architecture position SHINE as a versatile and readily translatable nanomedicine platform. Overall, this study establishes a blueprint for next-generation *in situ* cancer nanovaccines that integrate CDT-induced ICD with immunotherapy, offering a synergistic strategy for tumor eradication, metastasis control, and recurrence prevention.

## 5 Experimental section

### 5.1 RNA-sequencing and bioinformatic analysis

To investigate the molecular and immunological changes elicited by SHINE nanovaccine treatment, transcriptomic profiling was conducted on freshly isolated tumor tissues from control (C1–C3) and SHINE-treated (T1–T3) mice. Total RNA was extracted using TRIzol reagent (Invitrogen, Carlsbad, CA, USA), and RNA quality was evaluated using the TapeStation 4200 System (Agilent Technologies, Santa Clara, CA, USA) to ensure RNA integrity number (RIN) ≥ 7.0 prior to library preparation. Poly(A) + RNA was selectively enriched and reverse-transcribed using the CORALL RNA-Seq V2 Library Prep Kit (Lexogen GmbH, Vienna, Austria) to construct cDNA libraries. Sequencing was performed on an Illumina NextSeq 2000 platform (Illumina, San Diego, CA, USA) with 150-bp paired-end reads, yielding >6 Gb of raw data per sample. Raw FASTQ files were processed using fastp for adapter and quality trimming, and read quality was assessed with FastQC. Cleaned reads were aligned to the mouse reference genome (GRCm38/mm10) using STAR (v2.7.11b) under default splice-aware settings. Salmon (v1.10.2) was used in alignment-based mode to quantify gene-level expression from STAR-generated BAM files, producing both transcripts per million (TPM) and raw count matrices. Low-abundance genes were filtered out, and library sizes were normalized using the trimmed mean of M-values (TMM) method. Both counts per million (CPM) and TMM-normalized values were used for downstream analyses.

Differentially expressed genes (DEGs) were identified using ExDEGA software (v5.2.1, Ebiogen Inc., Seoul, Korea) with thresholds of |log_2_(fold change)| ≥ 1.2 and *p*-value <0.05. KEGG pathway enrichment was performed using the DAVID Bioinformatics Resources (v2021, NIH, Frederick, MD, USA), and results were reported as fold enrichment with −log_10_(p-value) significance. Gene Set Enrichment Analysis (GSEA) was conducted using GSEA v4.0.3 (Broad Institute), with significance defined as FDR q < 0.25, unless otherwise specified. Where applicable, protein–protein interaction (PPI) networks for leading-edge genes were constructed using STRING (high-confidence interactions) and visualized in Cytoscape to identify hub genes and interaction modules. ExDEGA and additional online tools (http://www.bioinformatics.com.cn) were used for integrated data analysis and graphical visualization.

### 5.2 Synthesis of biodegradable hollow MnO_2_ NPs and surface modification with PEG

Biodegradable hollow MnO_2_ nanoparticles (h-MnO_2_ NPs) were synthesized using SiO_2_ NPs as hard templates, following a previously reported method with slight modifications [60]. SiO_2_ NPs were prepared *via* a modified Stöber method by adding 7 mL deionized water (DI) and 14 mL aqueous ammonia to 350 mL ethanol, followed by dropwise addition of 7 mL tetraethyl orthosilicate (TEOS) under moderate stirring. After 6 h, a KMnO_4_ solution (3 mg mL^−1^, 140 mL) was added dropwise under vigorous stirring in the dark and stirred for another 6 h to allow MnO_2_ shell formation. The resulting SiO_2_@MnO_2_ core–shell particles were collected by centrifugation (12,000 rpm, 10 min) and redispersed in 280 mL of 2 м Na_2_CO_3_. The suspension was then heated at 60 °C in an oil bath with continuous stirring for 12 h to etch the silica core. The final h-MnO_2_ NPs were obtained by centrifugation (12,000 rpm, 10 min), washed several times with DI water until a neutral pH was achieved, and then redispersed in 64 mL of DI water. The h-MnO_2_ NPs were stored at room temperature for subsequent use.

Surface functionalization of h-MnO_2_ NPs was performed *via* a layer-by-layer assembly using the cationic polymer poly(allylamine hydrochloride) (PAH) and the anionic polymer poly(acrylic acid) (PAA). Briefly, 4 mL of h-MnO_2_ dispersion was mixed with 16 mL of PAH solution (6 mg mL^−1^) under sonication and stirred for 1.5 h. The particles were collected by centrifugation (14,000 rpm, 10 min), washed with DI water, and redispersed in 10 mL DI. The PAH-coated NPs were then added dropwise to 16 mL of PAA solution (104 μL PAA in DI) under sonication, stirred for 1.5 h, and purified similarly to obtain h-MnO_2_/PAH/PAA NPs. For PEGylation, 4 mL of EDC solution (5 mg mL^−1^) was added to the nanoparticle dispersion and stirred for 15 min to activate the carboxyl groups. This was followed by the addition of 4 mL NHS solution (7 mg mL^−1^) and continued stirring for another 15 min. Subsequently, 2 mL of H_2_N-PEG-COOH solution (20 mg mL^−1^) was introduced, and the mixture was stirred for 4 h at room temperature. The final PEGylated product (h-MnO_2_@PEG, denoted as M) was collected by centrifugation (14,000 rpm, 10 min), washed with DI, and redispersed in 8 mL DI water for further use.

### 5.3 Covalent conjugation of aPD-L1 to h-MnO_2_@PEG NPs

To covalently conjugate aPD-L1 antibody onto PEGylated hollow MnO_2_ NPs (h-MnO_2_@PEG), the terminal carboxyl groups on PEG were first activated by adding 2 mL of EDC solution (2.5 mg mL^−1^) and stirring for 15 min. Subsequently, 2 mL of NHS solution (4 mg mL^−1^) was introduced, and the reaction was stirred for an additional 15 min at room temperature. Then, 1 mL of aPD-L1 solution (100 μL antibody diluted in 900 μL PBS) was added, and the mixture was stirred for 4 h at 4 °C to facilitate amide bond formation. The resulting aPD-L1-functionalized NPs (h-MnO_2_@PEG@aPD-L1, denoted as MP) were collected by centrifugation (14,000 rpm, 10 min), washed thoroughly with DI water, and redispersed in 5 mL of DI water for further use. The amount of aPD-L1 antibody covalently conjugated to the NPs surface was quantified using the Pierce™ 660 nm Protein Assay Kit (Thermo Scientific, Rockford, Illinois, USA). Briefly, 10 μL of h-MnO_2_@PEG@aPD-L1 NPs (MP, 1 mg mL^−1^) was mixed with 150 μL of assay reagent in a 96-well plate and incubated for 15 min in the dark. Absorbance at 660 nm was then measured using a Varioskan LUX microplate reader (Thermo Fisher, Waltham, Massachusetts, USA). The conjugated antibody content was determined by referencing a standard curve generated with BSA under identical conditions.

### 5.4 GSH-responsive degradation of SHINE

The GSH-responsive degradation of SHINE was evaluated using transmission electron microscopy (TEM). SHINE was redispersed in PBS (pH 7.4) containing either 10 mм or 2 μм GSH to simulate the reductive intracellular tumor microenvironment and normal extracellular conditions, respectively. The suspensions were incubated at 37 °C with gentle shaking (100 rpm). At designated time points (0.5, 1, 2, and 4 h), samples were collected, centrifuged (14,000 rpm, 10 min), and the resulting pellets were gently washed with deionized water. The nanoparticles were then deposited onto carbon-coated copper grids and air-dried for TEM imaging (JEM-3010, JEOL Ltd., Tokyo, Japan).

### 5.5 Intracellular ROS generation

To evaluate intracellular reactive oxygen species (ROS) levels, 4T1 cells were seeded in 4-well chamber slides (SPL Life Sciences, Pocheon-si, Gyeonggi-do, South Korea) at a density of 1 × 10^5^ cells per well and allowed to adhere for 12 h at 37 °C in a humidified incubator with 5% CO_2_. Cells were then treated with the indicated nanoformulations (PBS, M, MR, MP, or SHINE) at a Mn-equivalent concentration of 100 μg mL^−1^ for 5 h. Following treatment, cells were rinsed twice with PBS and incubated with 10 μм DCFH-DA in serum-free medium for 30 min at 37 °C in the dark to enable intracellular deacetylation to DCFH. The excess probe was removed by triple PBS washes, and antifade mounting medium was applied. Green fluorescence of oxidized DCF, indicative of ROS accumulation was visualized using confocal laser scanning microscopy (CLSM, Carl Zeiss LSM700, Oberkochen, Germany).

For quantitative flow cytometry analysis, 4T1 cells were seeded in 6-well plates (SPL Life Sciences, Pocheon-si, Gyeonggi-do, South Korea) at 2 × 10^5^ cells per well, allowed to attach for 12 h, and treated under identical conditions. After DCFH-DA staining (10 μM, 30 min, 37 °C, dark), cells were washed with PBS, detached using trypsin-EDTA, neutralized, and resuspended in FACS buffer. The fluorescence intensity of oxidized DCF was quantified using a SONY SH800 flow cytometer (488 nm excitation; FITC channel; Sony Biotechnology, San Jose, CA, USA), with at least 10,000 viable singlets analyzed per sample using FSC/SSC gating.

### 5.6 Intracellular GSH detection

To assess intracellular GSH levels, 4T1 cells were seeded into 4-well chamber slides (SPL Life Sciences, Pocheon-si, Gyeonggi-do, South Korea) at a density of 1 × 10^5^ cells per well and allowed to adhere for 12 h at 37 °C in a humidified incubator with 5% CO_2_. Cells were then treated with the indicated nanoformulations (PBS, M, MR, MP, or SHINE) at a Mn-equivalent concentration of 100 μg mL^−1^ for 5 h. After treatment, cells were rinsed twice with PBS and incubated with ThiolTracker™ Violet (Thermo Scientific; Rockford, Illinois, USA); 20 μм in serum-free medium) for 30 min at 37 °C in the dark. Stained cells were washed three times with PBS, overlaid with antifade mounting medium, and imaged immediately using a confocal laser scanning microscope (Carl Zeiss LSM700; Carl Zeiss Inc., Oberkochen, Germany) with 405 nm excitation and 505–550 nm emission detection.

For quantitative GSH analysis, 4T1 cells were seeded in 6-well plates (2 × 10^5^ cells per well) and incubated for 12 h. Cells were then treated with the same nanoformulations (5 h, [Mn] = 100 μg mL^−1^), washed twice with PBS, harvested on ice, and processed using the Beyotime GSH/GSSG Assay Kit (Beyotime Biotechnology, Shanghai, China) following the manufacturer's protocol.

### 5.7 GSH depletion-enhanced CDT *in vitro*

To evaluate the CDT efficacy of free Mn^2+^ *vs.* the GSH-responsive SHINE nanovaccine, 4T1 cells were seeded in 96-well plates (6 × 10^3^ cells per well) and allowed to adhere for 12 h at 37 °C under 5% CO_2_. The culture medium was then replaced with fresh medium containing either MnCl_2_ or SHINE at equivalent Mn concentrations of 25, 50, 100, or 200 μg mL^−1^. PBS was used as a vehicle control. Cells were incubated for 24 h to permit intracellular GSH-triggered MnO_2_ reduction, Mn^2+^ release, and subsequent Fenton-like •OH generation. After treatment, cells were gently rinsed twice with PBS and incubated with CCK-8 reagent (10% v/v in DMEM) for 2 h at 37 °C. Absorbance was measured at 450 nm using a microplate reader (BioTek Synergy Neo2, Agilent Technologies, Santa Clara, CA, USA), and cell viability was expressed as a percentage relative to untreated controls.

### 5.8 Detection of immunogenic cell death (ICD) in cells

***Detection and quantitative analysis of ATP secretion.*** 4T1 cells were seeded in black, clear-bottom 96-well plates at a density of 8 × 10^3^ cells per well and allowed to adhere overnight at 37 °C in a humidified 5% CO_2_ incubator. Cells were then treated with the indicated formulations (M, MR, MP, or SHINE) for 24 h at a Mn-equivalent dose of 100 μg mL^−1^. For qualitative ATP imaging, the culture medium was replaced with 100 μL of phenol red–free DMEM, and an equal volume of freshly reconstituted reaction mix from the Luminescent ATP Detection Assay Kit (Abcam, Cambridge, MA, USA) was added according to the manufacturer's protocol. After 10 min of equilibration at room temperature in the dark, bioluminescence signals were recorded using an IVIS Lumina XR imaging system (PerkinElmer, Inc., Waltham, Massachusetts, USA) under auto-exposure with an open emission filter. For quantitative analysis, parallel cultures were treated as described above. Supernatants were harvested by centrifugation (1000×*g*, 5 min) and immediately mixed 1:1 with the ATP detection reagent in white 96-well plates according to the manufacturer's instructions (Abcam, Cambridge, MA, USA). After 5 min of incubation at room temperature, luminescence was measured using a plate luminometer (Varioskan LUX, Thermo Scientific, USA). ATP concentrations were determined based on a standard curve constructed from known ATP solution concentrations ranging from 10 pM to 1 μM prepared in culture medium.

***Detection and quantification of HMGB1 release.*** To quantify HMGB1 released during ICD, 4T1 cells were seeded in white 96-well plates at 8 × 10^3^ cells per well and allowed to adhere for 12 h at 37 °C in a humidified incubator with 5% CO_2_, using phenol red–free complete medium. Cells were then treated with the indicated formulations (M, MR, MP, or SHINE) at an equivalent Mn concentration of 100 μg mL^−1^ for 24 h. Following treatment, extracellular HMGB1 levels were measured using the Lumit® HMGB1 (Human/Mouse) Immunoassay Kit (Promega Corporation, Madison, WI, USA) in the direct, no-transfer format, following the manufacturer's protocol.

To visualize HMGB1 release, 4T1 cells were seeded in 4-well chamber slides (SPL Life Sciences, Pocheon-si, Gyeonggi-do, South Korea) at 1 × 10^5^ cells per well and allowed to adhere for 12 h at 37 °C in a humidified 5% CO_2_ incubator. Cells were then treated with the indicated formulations (M, MR, MP, or SHINE) at a Mn-equivalent dose of 100 μg mL^−1^ for 24 h. Following treatment, cells were washed three times with PBS and fixed with 4% paraformaldehyde (PFA) for 10 min at RT. Fixed cells were permeabilized using 0.1% Triton X-100 in PBS for 15 min, washed, and blocked with 5% BSA in PBS containing 0.05% Tween-20 (PBS-T) for 1 h. Cells were then incubated overnight at 4 °C with rabbit anti-HMGB1 antibody (Cambridge, MA, USA; 1:200 in PBS-T), followed by three 5-min PBS-T washes. Alexa Fluor 647–conjugated goat anti-rabbit IgG (Cambridge, MA, USA; 1:400 in PBS-T) was applied for 2 h at RT in the dark. After three additional PBS washes, nuclei were counterstained with DAPI-containing antifade mounting medium and sealed with coverslips. Confocal images were captured using a Carl Zeiss LSM700 laser scanning microscope (Carl Zeiss Inc., Oberkochen, Germany).

***Cell-surface CRT expression.*** 4T1 cells were seeded in 4-well chamber slides (SPL Life Sciences, Pocheon-si, Gyeonggi-do, South Korea) at a density of 1 × 10^5^ cells per well and allowed to adhere for 12 h at 37 °C in a humidified 5% CO_2_ atmosphere. Cells were then treated with the indicated formulations (M, MR, MP, or SHINE) at a Mn-equivalent dose of 100 μg mL^−1^ for 24 h. After treatment, cells were washed three times with PBS and fixed with 4% PFA for 10 min at RT. Following fixation, cells were blocked in 5% BSA prepared in PBS-T for 1 h at RT. Samples were then incubated overnight at 4 °C with rabbit anti-calreticulin antibody (Abcam, Cambridge, MA, USA; 1:200 in PBS-T). After three 5-min PBS-T washes, cells were stained with Alexa Fluor® 488–conjugated goat anti-rabbit IgG (Abcam, Cambridge, MA, USA; 1:400 in PBS-T) for 2 h at RT in the dark. Nuclei were counterstained with DAPI-containing antifade mounting medium, and coverslips were applied. Imaging was performed using a Carl Zeiss LSM700 confocal laser scanning microscope (Carl Zeiss Inc., Oberkochen, Germany).

### 5.9 *In vitro* comparison of direct CDT and CDT + T cell cytotoxicity

Effector CD8^+^ T cells were generated as described in the section “*In vitro CD8*^*+*^ *T cell activation by mature DCs”* (Supporting information). One day prior to the assay, 4T1-Luc cells were seeded in white opaque 96-well plates (1 × 10^4^ cells per well) in 100 μL of complete medium. To induce chemodynamic sensitization, adherent cells were treated with SHINE nanoparticles at Mn-equivalent concentrations of 50, 100, or 200 μg mL^−1^ for 5 h, followed by three PBS washes and replacement with fresh medium. These pretreated cells served as CDT-sensitized targets. Two groups were then established on the same plate:

- Group 1 (CDT only): CDT-pretreated 4T1-Luc cells received 100 μL of medium without T cells.
- Group 2 (CDT + T cells): CDT-pretreated 4T1-Luc cells were co-cultured with effector CD8^+^ T cells at an effector-to-target (E:T) ratio of 5:1 in 100 μL of medium (effector: E, CD8^+^ T cells; target: T, 4T1-Luc).

Co-cultures were incubated for 24 h at 37 °C under 5% CO_2_. Tumor cell viability was assessed by adding D-luciferin (Caliper Life Sciences, Hopkinton, Massachusetts, USA; 150 μg mL^−1^) and quantifying bioluminescence using an IVIS Lumina XR system (PerkinElmer, Inc., Waltham, Massachusetts, USA).

### 5.10 Tumor targeting and biodistribution of SHINE in 4T1-bearing mice

Female BALB/c mice (7 weeks old, 18–22 g) were subcutaneously inoculated with 4T1 cells in the right flank. Once tumors reached ∼200 mm^3^, mice were randomized into three groups (*n* = 5 per group) and administered a single intravenous (tail vein) injection of one of the following Cy5.5-labeled formulations at an equivalent Cy5.5 dose of 0.25 mg kg^−1^: free Cy5.5, MR@Cy5.5 NPs, or SHINE@Cy5.5 NPs. Whole-body fluorescence imaging was conducted at 2, 4, 8, 12, 18, 24, 48, and 72 h post-injection under isoflurane anesthesia (2–3% in oxygen) using the FOBI imaging system (CellGenetek Co., Ltd., Daejeon, South Korea). All images were captured using consistent exposure, binning, and aperture settings across groups in the Cy5.5 fluorescence channel. At 72 h, mice were euthanized, and major organs (liver, lungs, spleen, heart, and kidneys) along with tumor tissues were harvested, rinsed in PBS, and imaged *ex vivo* under identical conditions to assess biodistribution. Quantitative fluorescence intensity was analyzed using the system's region-of-interest tool.

### 5.11 Anti-tumor efficacy of SHINE in the 4T1 tumor model

A primary 4T1 tumor model was established by subcutaneously injecting 1 × 10^6^ 4T1 cells suspended in 100 μL of FBS-free DMEM and Matrigel (1:1, v/v) into the right flank of female BALB/c mice (6–8 weeks old, 18–22 g). When tumors reached ∼100 mm^3^ (designated as day 0), mice were randomly assigned to five groups (*n = 5* per group) and treated *via* intravenous tail vein injection with: (i) PBS (control), (ii) M (h-MnO_2_@PEG), (iii) MR (M loaded with R848), (iv) MP (M conjugated with aPD-L1), or (v) SHINE (M co-loaded with R848 and aPD-L1). Treatments were administered on days 0, 2, 4, 6, and 8 (200 μL per dose, totaling five doses). Each nanoparticle formulation was dosed to deliver 5 mg kg^−1^ Mn, 2.2 mg kg^−1^ R848, and 1.0 mg kg^−1^ aPD-L1. Tumor dimensions were measured every two days using digital calipers, and tumor volume (V) was calculated as V = (W^2^ × L)/2, where W represents the width and L represents the length. Body weight was monitored throughout the treatment period to evaluate systemic toxicity. On day 20, mice were euthanized, and tumors were excised, weighed, and photographed. Tumor tissues were processed to prepare single-cell suspensions for flow cytometry, fixed in 10% neutral-buffered formalin for histological analysis, or snap-frozen for RNA sequencing. Major organs (lungs, liver, spleen, heart, and kidneys) were also collected for pathological and biosafety assessments.

### 5.12 Intratumoral DC–T cells mapping *via* flow cytometry and t-SNE analysis

Tumor single-cell suspensions were first stained with Fixable Viability Dye eFluor™ 450 (Thermo Fisher Scientific, Waltham, MA, USA; 1:1000 dilution) for 30 min at 4 °C in the dark, followed by incubation with anti-CD16/32 (Fc block) for 15 min at 4 °C to minimize nonspecific antibody binding. Cells were then stained with the following antibody panel (all from BioLegend Inc., San Diego, CA, USA; 1:80 dilution): anti-CD3-APC (T cell lineage gate), anti-CD4-FITC (helper T cells), anti-CD8α-PE/Cy7 (cytotoxic T cells), anti-CD11c-PE (dendritic cell marker), and anti-CD86-PerCP (DC maturation marker; CD86^+^ for mDCs, CD86^−^ for iDCs). Staining was performed for 45 min at 4 °C in the dark. After washing, samples were analyzed on a FACSAria Fusion cytometer (BD Biosciences, Franklin Lakes, NJ, USA). Dimensionality reduction and visualization of immune cell populations were conducted using t-distributed Stochastic Neighbor Embedding (t-SNE) in FlowJo, v10 software.

### 5.13 Anti-metastatic efficacy of SHINE in the lung metastasis model

Female BALB/c mice (6–8 weeks old, 18–22 g) were first subcutaneously inoculated in the right flank with 1 × 10^6^ 4T1 cells suspended in 100 μL of FBS-free DMEM:Matrigel (1:1, v/v) on day −6 to form a primary tumor. To establish a metastatic cancer model, 1.5 × 10^5^ 4T1 cells in 100 μL PBS were administered *via* the tail vein on day −2. Mice were randomly assigned (*n = 5* per group) to receive one of the following treatments: (i) PBS, (ii) M (h-MnO_2_@PEG), (iii) MR (h-MnO_2_@PEG loaded with R848), (iv) MP (h-MnO_2_@PEG conjugated with aPD-L1), or (v) SHINE (h-MnO_2_@PEG co-loaded with R848 and aPD-L1). All formulations were administered intravenously on days 0, 2, 4, 6, and 8 (200 μL per dose in sterile PBS), delivering 5 mg kg^−1^ Mn, 2.2 mg kg^−1^ R848, and 1.0 mg kg^−1^ aPD-L1. Body weight and overall health status were monitored every other day. On day 20, mice were euthanized, and lungs were excised, rinsed with PBS, inflated, and fixed in Bouin's solution (Sigma-Aldrich, St. Louis, MO, USA) for 24 h. Whole lungs were imaged, and metastatic nodules were counted across all lobes. Lung wet weight was also recorded.

### 5.14 Anti-tumor and immune-memory efficacy of SHINE in the rechallenge model

Primary tumors were established by subcutaneously injecting 1 × 10^6^ 4T1 cells suspended in 100 μL of FBS-free DMEM:Matrigel (1:1, v/v) into the right flank of female BALB/c mice (6–8 weeks old) on day −6. Once tumors reached ∼100 mm^3^ (day 0), mice were randomized (*n = 12* per group) and intravenously treated with PBS, M (h-MnO_2_@PEG), MR (h-MnO_2_@R848), MP (h-MnO_2_@aPD-L1), or SHINE (h-MnO_2_@R848@aPD-L1). Treatments were administered on days 0, 2, 4, 6, and 8 (five total doses; 200 μL PBS per injection). The SHINE dose corresponded to 5 mg kg^−1^ Mn, 2.2 mg kg^−1^ R848, and 1.0 mg kg^−1^ aPD-L1. Tumor growth was monitored every other day using calipers. On day 20, half of the animals (*n = 6* per group) were sacrificed for splenocyte isolation and memory T cell analysis. The remaining mice underwent complete surgical resection of the primary tumors under isoflurane anesthesia. To evaluate long-term immune memory, SHINE-treated mice were rechallenged on day 30 by subcutaneous injection of 1 × 10^6^ 4T1 cells into the contralateral (left) flank. Age- and sex-matched naïve mice receiving the same cell inoculation served as controls. Tumor progression and tumor-free survival were monitored until day 90 or until humane endpoints were reached.

### 5.15 *In vivo* effector memory T cell analysis

On day 20 post-treatment, spleens were collected, and single-cell suspensions were prepared in staining buffer. Cells were stained with Fixable Viability Dye eFluor™ 450 (Thermo Fisher Scientific, Waltham, MA, USA; 1:1000 dilution) for 30 min at 4 °C in the dark. To block nonspecific Fc receptor binding, cells were incubated with anti-CD16/32 for 15 min at 4 °C. Subsequently, cells were stained for 45 min at 4 °C in the dark using a cocktail of fluorophore-conjugated antibodies: anti-CD3-PerCP/Cy5.5, anti-CD8-FITC, anti-CD44-APC, and anti-CD62L-PE (BioLegend, San Diego, CA, USA; 1:80 dilution each). After three washes with PBS, samples were analyzed on a BD FACSAria™ Fusion flow cytometer (BD Biosciences Ltd., Franklin Lakes, NJ, USA), and data were processed using FlowJo software. CD8^+^ T cells (CD3^+^CD8^+^) were gated, and within this population, effector memory T cells (T_EM_; CD3^+^CD8^+^CD44^+^CD62L^−^) were quantified.

### 5.16 Statistical analysis

All quantitative data are presented as the mean ± standard deviation (SD). Statistical analyses were conducted using GraphPad Prism 8.0 (GraphPad Software, San Diego, CA, USA). Comparisons between two groups were performed using unpaired two-tailed Student's t-test. For comparisons involving three or more groups, one-way ANOVA followed by Tukey's post hoc test was used. All experiments were conducted in at least triplicate. Statistical significance was defined as follows: ∗*p*< 0.05, ∗∗*p*< 0.01, ∗∗∗*p*< 0.001, and ∗∗∗∗*p* < 0.0001; differences with *p* ≥ 0.05 were considered not significant (ns).

## CRediT authorship contribution statement

**Khang-Yen Pham:** Writing – review & editing, Writing – original draft, Visualization, Validation, Project administration, Methodology, Investigation, Formal analysis, Data curation, Conceptualization. **Thu Huyen Le Thi:** Writing – review & editing, Visualization, Validation, Investigation. **Anil Giri:** Writing – review & editing, Visualization, Validation. **Jongjun Park:** Writing – review & editing, Visualization, Validation. **Jong-Sun Kang:** Writing – review & editing, Visualization, Validation. **Taeg Kyu Kwon:** Writing – review & editing, Visualization, Methodology. **Jee-Heon Jeong:** Writing – review & editing, Writing – original draft, Visualization, Validation, Funding acquisition, Conceptualization. **Simmyung Yook:** Writing – review & editing, Writing – original draft, Visualization, Validation, Supervision, Methodology, Investigation, Funding acquisition, Data curation, Conceptualization.

## Data availability statement

All data supporting the findings of this study are available within the Article and its Supplementary Information files. Additional data and source files are available from the corresponding author upon reasonable request.

## Ethics approval and consent to participate

This research complies with ethical regulations. All animal procedures were conducted in accordance with institutional ethical guidelines and approved by the Institutional Animal Care and Use Committee (IACUC) of Sungkyunkwan University (Protocol Nos. SKKUIACUC2023-12-12-1 and SKKUIACUC2025-03-04-1).

## Declaration of competing interest

The authors declare no conflict of interest.
