# Supplementary material for: GSH-responsive nanovaccine triggers immunogenic cell death and potent memory T cell immunity for durable, recurrence-free tumor eradication
Source: Bioact Mater. 2026 Apr 30;64:135–58. doi: 10.1016/j.bioactmat.2026.04.025 (PMC13145900; doi:10.1016/j.bioactmat.2026.04.025)
Supplement: Multimedia component 1 [file mmc1.zip › 6-Nanovaccine-Supplementary-R1-FINAL-Clear.docx]

*Supporting information for*

**GSH-responsive nanovaccine triggers immunogenic cell death and potent memory T cell immunity for durable, recurrence-free tumor eradication**

**1. Experimental section**

**Materials**

Tetraethyl orthosilicate (TEOS), potassium permanganate (KMnO_4_), sodium carbonate (Na_2_CO_3_), glutathione (GSH), methylene blue (MB), ammonium hydroxide solution (25% NH_3_ in H_2_O), and 2′,7′-dichlorodihydrofluorescein diacetate (DCFH-DA) were purchased from Sigma-Aldrich (St. Louis, MO, USA). Poly(allylamine hydrochloride) (PAH, MW: 15,000), poly(acrylic acid) (PAA, MW: 1,800), Pierce^TM^ 660 nm Protein Assay Kit, and ThiolTracker^TM^ Violet were obtained from Thermo Scientific (Rockford, Illinois, USA). GSH/GSSG Assay Kit was acquired from Beyotime (Beyotime Biotechnology, Shanghai, China). Amino-terminated polyethylene glycol acid (NH_2_–PEG–COOH, MW: 5,000) was supplied by nanoCS (Chapel Hill, NC, USA). 1-Ethyl-3-(3-dimethylaminopropyl)carbodiimide hydrochloride (EDC) and N-hydroxysuccinimide (NHS) were purchased from TCI Co., Ltd. (Tokyo, Chuo, Japan). The Toll-like receptor 7/8 agonist resiquimod hydrochloride (R848·HCl) was obtained from InvivoGen (San Diego, CA, USA). Anti-mouse PD-L1 monoclonal antibody (clone 10F.9G2, InVivoMAb) was acquired from Bio X Cell (Lebanon, NH, USA). Cy5.5-N-succinimidyl ester (Cy5.5-NHS) was purchased from Lumiprobe Corporation (Hunt Valley, MD, USA). Dulbecco's Modified Eagle Medium (DMEM), fetal bovine serum (FBS), antibiotic–antimycotic, and 0.25% trypsin–EDTA were obtained from Gibco-Invitrogen (Grand Island, NY, USA). Ethanol was purchased from Daejung Chemicals & Metals Co., Ltd. (Siheung, Gyeonggi-do, South Korea). The QuantiMax^TM^ WST-8 Cell Viability Assay Kit was sourced from Biomax (Guri-si, Gyeonggi-do, South Korea). Luminescent ATP Detection Assay Kit, rabbit anti-HMGB1 antibody, and rabbit anti-calreticulin (CRT) antibody were obtained from Abcam (Cambridge, MA, USA). The Lumit^®^ HMGB1 (Human/Mouse) Immunoassay Kit was purchased from Promega Corporation (Madison, WI, USA). Corning^®^ Matrigel^®^ Basement Membrane Matrix (LDEV‑free) was purchased from Corning Inc. (Tewksbury, MA, USA).

**Cells and animals**

Murine breast cancer (4T1), luciferase-expressing 4T1 (4T1-Luc), and murine fibroblast (3T3) cell lines were cultured in Dulbecco’s Modified Eagle Medium (DMEM) supplemented with 10% fetal bovine serum (FBS) and 1% antibiotic–antimycotic solution. All cells were maintained in a humidified incubator at 37 °C with 5% CO_2_.

Female BALB/c mice (6–8 weeks old, 18–22 g) were purchased from OrienBio Inc. (Jungwon-gu, Gyeonggi-do, South Korea) and housed under specific pathogen-free conditions at the Animal Center. All animal procedures were conducted in accordance with institutional ethical guidelines and approved by the Institutional Animal Care and Use Committee (IACUC).

**Characterizations**

The surface morphology, particle size, and internal structure of the nanoparticles were characterized using transmission electron microscopy (TEM; JEM-3010, JEOL Ltd., Tokyo, Japan) and scanning electron microscopy (SEM; JSM‑IT800, JEOL Ltd., Tokyo, Japan). Elemental distribution was assessed via energy-dispersive X-ray spectroscopy (EDS) using a high-resolution transmission electron microscope (HRTEM; JEM-2100F, JEOL Ltd., Tokyo, Japan). Dynamic light scattering (DLS) and zeta potential measurements were conducted using a Zetasizer Nano ZS instrument (Malvern Instruments, UK). The manganese (Mn) content in the nanoparticles was quantified using inductively coupled plasma–optical emission spectrometry (ICP-OES; Agilent 5100, PerkinElmer, Waltham, Massachusetts, USA). XPS spectra were acquired using an ESCALAB 250Xi spectrometer (Thermo Fisher Scientific, MA, USA). Cellular uptake and localization studies were performed using confocal laser scanning microscopy (CLSM; LSM700, Carl Zeiss Inc., Oberkochen, Baden-Württemberg, Germany). Flow cytometric analysis was conducted on a FACSAria Fusion system (BD Biosciences Ltd., Franklin Lakes, NJ, USA). *In vivo* fluorescence biodistribution was monitored using the FOBI imaging system (CellGenetek Co., Ltd., Daejeon, South Korea), while bioluminescence imaging was performed using the IVIS Lumina XR system (PerkinElmer, Inc., Waltham, Massachusetts, USA). Ultraviolet-visible (UV-vis) absorption spectra were recorded using a UV-3600i Plus spectrophotometer (Shimadzu Scientific Corp., Kyoto, Japan). Fluorescence emission spectra were recorded using a FluoroMax-4 spectrofluorometer (Horiba Scientific, Piscataway, New Jersey, USA). Quantification of R848 was carried out using high-performance liquid chromatography (HPLC; Vanquish, Thermo Scientific, Rockford, IL, USA).

**R848 loading into h-MnO_2_@PEG@aPD-L1 NPs**

To load R848 into h-MnO_2_@PEG@aPD-L1 NPs (MP), 200 μL of R848·HCl solution (4 mg/mL in DI water) was added dropwise to 5 mL of MP colloidal suspension under moderate stirring. The mixture was stirred at 4 °C for 12 h. The resulting R848-loaded NPs (h-MnO_2_@PEG@R848@aPD-L1, denoted as SHINE) were collected by centrifugation (14,000 rpm, 10 min), washed twice with DI water, and redispersed in 2 mL of PBS for further use. To determine the R848 loading efficiency, freeze-dried SHINE NPs were weighed and redispersed in 1 mL of 10 mM GSH solution. After incubation at 37°C with gentle shaking (100 rpm) for 2 h, the suspension was centrifuged (14,000 rpm, 10 min), and the supernatant containing released R848 was collected and quantified by high-performance liquid chromatography (HPLC; Vanquish, Thermo Scientific, Rockford, IL, USA) using a standard calibration curve prepared under identical conditions. The encapsulation efficiency (EE%) and loading capacity (LC%) of R848 were calculated as follows:

EE (%) = (mass of R848 loaded / initial mass of R848 added) × 100%,

LC (%) = (mass of R848 loaded / total mass of SHINE) × 100%.

**SDS-PAGE analysis**

SHINE (MnO₂@R848@aPD-L1) were suspended in SDS sample buffer with or without dithiothreitol (DTT) and heated at 95 °C for 10 min to denature surface-conjugated proteins. Equal protein amounts (20 μg per sample) were loaded onto a 10% SDS-polyacrylamide gel and electrophoresed at 80 V for 30 min, followed by 120 V for 1 h. Free aPD-L1 served as a molecular reference. Following separation, the gel was stained with Pierce^TM^ Coomassie Brilliant Blue (Thermo Scientific, Rockford, Illinois, USA) for 30 min, rinsed three times with deionized water, and decolorized overnight prior to imaging.

**Immunoreactivity assay of aPD-L1-conjugated SHINE**

The immunoreactivity of surface-conjugated aPD-L1 was evaluated using PD-L1-expressing 4T1 cells. Briefly, 4T1 cells were seeded in 6-well plates (2 × 10^5^ cells per well) and incubated overnight. The cells were then collected and resuspended in FACS buffer. Free aPD-L1 or SHINE was incubated with the cells at low (aPD-L1-equivalent, 10 µg mL^-1^) or high (aPD-L1-equivalent, 20 µg mL^-1^) concentrations for 60 min at 4 °C. PBS and non-antibody-conjugated MnO_2_ (M) were used as negative controls. After washing with cold FACS buffer to remove unbound materials, the cells were stained with Alexa Fluor 488 anti-rat IgG secondary antibody (Thermo Fisher Scientific, Rockford, IL, USA) for 30 min at 4 °C in the dark. The cells were then washed, resuspended in FACS buffer, and analyzed using a SONY SH800 flow cytometer (Sony Biotechnology, San Jose, CA, USA).

**GSH depletion and Mn^2+^ release**

To evaluate the GSH-responsiveness of SHINE NPs, both GSH consumption and Mn^2+^ ion release were assessed under reductive conditions simulating the tumor microenvironment. Freeze-dried SHINE NPs were redispersed in 1.5 mL of 10 mM GSH in PBS (pH 7.4) and incubated at 37 °C with gentle shaking (100 rpm). At designated time points (2 min, 30 min, 1 h, 2 h, 4 h, and 8 h), samples were centrifuged (14,000 rpm, 10 min), and the supernatants were collected for analysis. To maintain reductive conditions, the extracted volume was replenished with fresh GSH solution after each sampling. GSH levels were determined using Ellman’s reagent [5,5′-dithiobis-(2-nitrobenzoic acid), DTNB]. Briefly, 100 μL of each supernatant was mixed with 100 μL of DTNB solution (4 mM) and incubated at 25 °C for 5 min. Absorbance at 412 nm was measured using a microplate reader (Varioskan LUX, Thermo Fisher Scientific, USA), and GSH concentrations were calculated from a standard curve prepared under identical conditions. Mn^2+^ release was quantified using inductively coupled plasma–optical emission spectroscopy (ICP-OES; Agilent 5100, PerkinElmer, Waltham, Massachusetts, USA). The percentage of Mn^2+^ released was calculated relative to the total Mn content in the SHINE.

**GSH-responsive R848 release *in vitro***

For *in vitro* release studies, freeze-dried SHINE nanoparticles were redispersed in 1.5 mL of 10 mM GSH solution and incubated at 37 °C with gentle shaking (100 rpm). At defined time points (2 min, 30 min, 1 h, 2 h, 4 h, 6 h, and 12 h), the suspension was centrifuged (14,000 rpm, 10 min), and the supernatant was collected for R848 quantification by HPLC (Vanquish, Thermo Scientific, Rockford, IL, USA). An equal volume of fresh GSH solution was replenished after each sampling. Control experiments were conducted similarly using 2 μM GSH to mimic normal physiological conditions. The cumulative release of R848 was calculated using the formula:

Cumulative release (%) = (mass of released R848 / total mass R848 in SHINE) × 100%.

**Extracellular** •**OH** **generation assay**

To assess the Fenton-like activity of Mn^2+^, methylene blue (MB, 20 µg mL^-1^) was added to a 25 mM NaHCO_3_/CO_2_ buffer containing MnCl_2_ ([Mn] = 100 µg mL^-1^) and H_2_O_2_ (8 mM). The mixture was incubated at 37 °C for 30 min under gentle shaking, followed by centrifugation (14,000 rpm, 5 min) to remove particulates. UV–vis absorption spectra (400–900 nm) were recorded (UV-3600i Plus spectrophotometer; Shimadzu Scientific Corp., Kyoto, Japan), and the absorbance at 665 nm was monitored to quantify •OH-induced MB degradation.

To evaluate •OH generation mediated by SHINE nanoparticles, SHINE ([Mn] = 100 µg mL^-1^) was first incubated in 25 mM NaHCO_3_/CO_2_ buffer containing GSH (0, 5, or 10 mM) at 37 °C for 30 min to induce GSH-triggered MnO_2_ reduction. After centrifugation (14,000 rpm, 5 min), the resulting supernatants were collected and mixed with MB (20 µg mL^-1^) and H_2_O_2_ (8 mM). The reaction was allowed to proceed for an additional 30 min at 37 °C. MB degradation was then quantified by measuring the decrease in absorbance at 665 nm *via* UV–vis spectroscopy.

**Cellular uptake of SHINE**

To visualize nanoparticle internalization, C6-loaded formulations (M@C6, MR@C6, MP@C6, SHINE@C6) were prepared as previously described and used as fluorescent tracers. 4T1 cells were seeded into 4-well glass chamber slides (SPL Life Sciences, Pocheon-si, Gyeonggi-do, South Korea) at a density of 1 × 10^5^ cells per well and allowed to adhere for 12 h at 37 °C under 5% CO_2_. Cells were then incubated with the indicated formulations for 5 h at a C6-equivalent concentration of 1.5 µg mL⁻¹. After four PBS washes, cells were fixed with 4% paraformaldehyde for 10 min at room temperature, rinsed, and mounted using antifade mounting medium containing DAPI. Confocal images were acquired using a confocal laser scanning microscope (CLSM) (Carl Zeiss LSM700; Carl Zeiss Inc., Oberkochen, Germany).

For quantitative analysis, 4T1 cells were seeded into 6-well plates at 2 × 10^5^ cells per well and allowed to adhere for 12 h before treatment with C6-labeled formulations (1.5 µg mL^-1^, 5 h). Following four PBS washes, cells were harvested by trypsinization, neutralized, centrifuged, and resuspended in FACS buffer. Mean fluorescence intensity (MFI) of C6 was measured using a SONY SH800 flow cytometer (Sony Biotechnology, San Jose, CA, USA), collecting 10,000 gated single, live cells per sample (FSC/SSC gating).

To assess PD-L1-mediated uptake, parallel cultures were pretreated with free anti-PD-L1 antibody (1 mg mL^-1^, 2 h, 37 °C) to block surface PD-L1, followed by incubation with SHINE@C6 under identical conditions. Uptake was then analyzed *via* CLSM and flow cytometry as described above.

***In vitro* cytotoxicity of SHINE**

The cytotoxicity of SHINE nanoparticles was evaluated in both normal (3T3) and cancerous (4T1) cell lines using a Cell Counting Kit-8 (CCK-8) assay. Cells were seeded into 96-well plates at a density of 6 × 10^3^ cells per well in complete growth medium and allowed to adhere for 12 h at 37 °C under 5% CO_2_. Subsequently, the medium was replaced with fresh medium containing SHINE at varying Mn concentrations (25, 50, 100, or 200 µg mL^-1^), while PBS-treated wells served as the control. After 24 h of incubation, cells were gently rinsed twice with PBS and incubated with 10% (v/v) CCK-8 reagent diluted in fresh medium for 2 h at 37 °C. Absorbance was measured at 450 nm using a microplate reader (BioTek Synergy Neo2, Agilent Technologies, Santa Clara, CA, USA) to determine cell viability.

**Live/dead viability assay**

4T1 cells were seeded in 96-well plates at a density of 6 × 10^3^ cells per well and allowed to adhere for 12 h at 37 °C in a humidified 5% CO_2_ incubator. Cells were then treated with the indicated formulations (PBS, M, MR, MP, or SHINE) at a Mn-equivalent concentration of 100 µg mL^-1^ for 24 h. Following treatment, cells were rinsed twice with PBS and stained with a Calcein-AM/propidium iodide (PI) working solution according to the manufacturer’s protocol. After a 30 min incubation at room temperature in the dark, cells were gently washed with PBS and immediately imaged using a confocal laser scanning microscope (Carl Zeiss LSM700; Carl Zeiss Inc., Oberkochen, Germany).

**Apoptosis analysis by flow cytometry**

4T1 cells were seeded in 6-well plates at a density of 2 × 10^5^ cells per well and allowed to adhere for 12 h at 37 °C in a humidified 5% CO_2_ incubator. Cells were then treated with the indicated formulations (PBS, M, MR, MP, or SHINE) at a Mn-equivalent concentration of 100 µg mL^-1^ for 24 h. Following treatment, both floating and adherent cells were collected and pelleted by centrifugation (300 × g, 3 min, 4 °C). Pellets were washed twice with ice-cold PBS and resuspended in 1× Annexin V binding buffer at a concentration of approximately 1 × 10^6^ cells mL^-1^. For staining, 5 µL of Annexin V–FITC and 5 µL of propidium iodide (PI) were added to 100 µL of the cell suspension and incubated for 15 min at room temperature in the dark. Immediately before flow cytometric acquisition, 400 µL of binding buffer was added. Samples were analyzed using a SONY SH800 flow cytometer (Sony Biotechnology, San Jose, CA, USA). Unstained, FITC-only, and PI-only controls were included for compensation and gating. At least 10,000 events were collected per sample after doublet exclusion (FSC-H vs. FSC-A) and debris removal. Data were processed using FlowJo software (BD Biosciences, USA) to quantify the proportions of viable (Annexin V^-^/PI^-^), early apoptotic (Annexin V^+^/PI^-^), late apoptotic (Annexin V^+^/PI^+^), and necrotic (Annexin V^-^/PI^+^) cells. The total apoptosis rate was calculated as the sum of early and late apoptotic populations.

**Wound-healing assay for anti-metastatic activity**

The migratory capacity of 4T1 cells was assessed using a wound-healing assay with SPLScar^TM^ Blocks (SPL Life Sciences, Korea). Briefly, 4T1 cells (5 × 10^4^ per well) were seeded into the blocks and allowed to adhere for 12 h at 37 °C, 5% CO_2_. The blocks were then carefully removed to create parallel, cell-free gaps (500 µm) on the culture surface. Monolayers were rinsed with PBS to remove detached cells and treated with DMEM supplemented with 2% FBS and the indicated formulations (M, MR, MP, or SHINE) at a Mn-equivalent concentration of 100 µg mL⁻¹. Phase-contrast images were captured at 0 h and 24 h using an optical microscope. Wound areas were quantified using ImageJ software (National Institutes of Health, Bethesda, MD, USA).

***In vitro* dendritic cell maturation**

Immature dendritic cells (iDCs) were isolated from the bone marrow of naïve BALB/c mice (6–7 weeks old) as previously described [1]. Briefly, bone marrow was collected from femurs and tibias, treated with red blood cell lysis buffer, and cultured in complete medium supplemented with granulocyte-macrophage colony-stimulating factor (GM-CSF, 20 ng mL⁻^1^) at 37 °C. On day 6, non-adherent iDCs were harvested for use. To simulate antigen/danger signal transfer from tumor cells to DCs, a transwell co-culture system was employed. 4T1 cells (9 × 10^5^ cells/well) were seeded in the upper compartment of 0.4-μm pore transwell inserts, and iDCs (9 × 10^5^ cells/well) were seeded in the lower wells. The upper chambers were treated with the indicated nanoformulations (M, MR, MP, or SHINE; [Mn] = 100 μg mL⁻^1^) for 24 h. Following incubation, DCs in the lower chamber were collected on ice, stained with Fixable Viability Dye eFluor™ 450 (Thermo Fisher Scientific, Waltham, MA, USA; 1:1000 dilution) for 30 min at 4 °C in the dark, and washed with PBS. The cells were then incubated with anti-mouse CD16/32 to block nonspecific Fc receptor binding, followed by staining with anti-CD11c-APC, anti-CD80-PE, and anti-CD86-FITC antibodies (BioLegend, San Diego, CA, USA; 1:80 dilution) for 30 min at 4 °C in the dark. After washing, samples were analyzed using a FACSAria Fusion flow cytometer (BD Biosciences, Franklin Lakes, NJ, USA), and the data were processed using FlowJo software. DC maturation was defined as the CD11c⁺CD80^+^CD86^+^ population after sequential gating on debris exclusion, singlets, and live cells.

**Western blot analysis of STING pathway activation in BMDCs**

Bone marrow-derived dendritic cells (BMDCs; 1 × 10^6^ cells per well) were seeded in 6-well plates and allowed to adhere overnight. Cells were then treated with PBS, MnCl_2_ (positive control), or SHINE at a Mn-equivalent concentration of 100 μg mL^-1^ for 24 h. After treatment, cells were washed with cold PBS and lysed in RIPA buffer supplemented with a protease/phosphatase inhibitor cocktail on ice (30 min). Lysates were obtained by centrifugation (13,500 rpm for 15 min at 4 °C), and protein concentrations were determined using a BCA assay kit. Equal amounts of protein (20 μg per lane) were mixed with SDS loading buffer and denatured at 95 °C for 5 min, then separated on a 10% SDS-PAGE gel (60 V for 30 min, followed by 100 V for 90 min). Proteins were transferred onto PVDF membranes at 100 V for 120 min. Membranes were blocked in 5% BSA in TBS-T (0.1% Tween-20) for 1 h at room temperature and incubated overnight at 4 °C with primary antibodies against p-IRF3, IRF3, p-STING, STING (each 1:1000), and GAPDH (1:6000) (Cell Signaling Technology, Danvers, MA, USA), all diluted in 5% BSA. After washing with TBS-T (0.1%) three times, membranes were incubated with HRP-conjugated secondary antibodies (1:10,000, diluted in 3% BSA) for 90 min at room temperature. Following three additional washes with TBS-T (0.1%), protein bands were detected using enhanced chemiluminescence reagents and imaged with a luminescence image analyzer (LAS-3000 Lite; FUJIFILM, Tokyo, Japan).

***In vitro* CD8^+^ T cell activation by mature DCs**

CD8^+^ T cells were isolated from mouse spleens and lymph nodes of naïve BALB/c mice using a MojoSort™ negative-selection kit according to the manufacturer’s protocol (BioLegend, San Diego, USA). Purified T cells were co-cultured with matured DCs (mDCs) obtained from the above transwell assay at a DC:T cell ratio of 1:5 in complete RPMI medium. After 48 h of co-culture, cells were collected, washed, stained with Fixable Viability Dye eFluor™ 450 (Thermo Fisher Scientific, Waltham, MA, USA; 1:1000 dilution) for 30 min at 4 °C in the dark, and blocked with anti-CD16/32 (1:160 dilution, 10 min, 4 °C) to prevent nonspecific Fc binding. Cells were then stained with anti-CD8a-PE/Cy7, anti-CD25-APC, and anti-CD69-FITC antibodies (BioLegend, San Diego, USA; 1:80 dilution each) for 30 min at 4 °C in the dark. After washing, samples were analyzed on a FACSAria Fusion (BD Biosciences, USA). T cell activation was quantified as the percentage of CD69^+^CD8^+^ and CD25^+^CD8^+^ populations.

***In vitro* cytokine production by activated T cells**

To assess intracellular cytokine expression, CD8^+^ T-cell/mDC co-cultures were restimulated for 4 h with Phorbol 12-myristate 13-acetate (PMA, 50 ng mL⁻^1^), Ionomycin (1 μg mL⁻^1^), and Golgi stop (10 μg mL⁻^1^) at 37 °C. Then, cells were stained with Fixable Viability Dye eFluor™ 450 (Thermo Fisher Scientific, Waltham, MA, USA; 1:1000 dilution) for 30 min at 4 °C in the dark. Next, cells were Fc-blocked with anti-CD16/32 (1:160 dilution, 10 min, 4 °C), followed by surface staining with anti-CD8a-PE/Cy7 (BioLegend, San Diego, USA; 1:160 dilution) for 30 min at 4 °C. Cells were then fixed and permeabilized using BD Cytofix/Cytoperm™ buffer (BD Biosciences, USA), and intracellularly stained with anti-Granzyme B-PerCP/Cy5.5 and anti-Perforin-PE antibodies (BioLegend, San Diego, USA; 1:80 dilution each) for 30 min at 4 °C. Following washes with Cytoperm/Cytowash™, samples were analyzed on a FACSAria Fusion cytometer (BD Biosciences, USA). Data were reported as the percentage of Granzyme B^+^ or Perforin^+^ cells within gated CD8^+^ T cells.

**Pharmacokinetic analysis of SHINE**

To evaluate the pharmacokinetic profile of SHINE, 4T1 tumor-bearing mice (tumor volume, ~100 mm^3^) were intravenously injected with SHINE at an Mn-equivalent dose of 5.0 mg kg^-1^. Blood samples (75 μL) were collected at 5 and 30 min and 1, 2, 4, 8, 12, and 24 h after injection, digested with concentrated HNO_3_ and H_2_O_2_, and diluted to 2 mL with 2% HNO_3_. The digested samples were passed through a 0.2 μm filter, and Mn concentrations were quantified by ICP-OES. Pharmacokinetic parameters, including the distribution and elimination half-lives, were obtained by fitting the blood concentration-time profiles to a two-compartment model.

**Hemolysis assay**

Whole blood was collected from healthy female BALB/c mice into EDTA-coated tubes and immediately diluted 1:1 with sterile PBS (pH 7.4). Red blood cells (RBCs) were isolated by centrifugation (2,000 rpm, 10 min, 3×), with the plasma and buffy coat discarded after each spin. The final RBC pellet was resuspended 1:1 in PBS to prepare the RBC stock suspension. For hemolysis testing, 500 μL of RBC suspension was mixed with 500 μL of SHINE dispersion in PBS at Mn-equivalent concentrations of 100, 200, 400, or 800 μg mL⁻¹. PBS and deionized water were used as negative and positive controls, respectively. Samples were incubated at 37 °C for 5 h, followed by centrifugation (14,000 rpm, 10 min). The supernatants were collected and transferred to a clear 96-well plate. Hemoglobin release was quantified by measuring absorbance at 540 nm using a microplate reader (Varioskan LUX, Thermo Scientific, USA). Hemolysis percentage was calculated using the following equation:

$$\mathrm{Hemolysis}\left( \% \right)=\frac{A_{\mathrm{sample}}-A_{\mathrm{PBS}}}{A_{H_{2}O}-A_{\mathrm{PBS}}} \times100,$$

where A(H_2_O) and A(PBS) are the absorbances of positive and negative controls, respectively.

**Blood biochemical analysis**
On day 20 after intravenous injection, blood from PBS- and SHINE-treated mice was collected *via* retro-orbital bleeding. Whole blood was allowed to clot at room temperature and then centrifuged at 3000 × g for 5 min to obtain serum. Serum levels of liver function markers (AST, ALT, ALP, and T-Bil) and kidney function markers (UA, CREA, and BUN) were quantified using an automated clinical chemistry analyzer (AU480, Beckman Coulter, CA, USA) with commercially available reagent kits (Sekisui Medical Co., Ltd., Tokyo, Japan), following the manufacturer’s instructions.

**Single-cell suspension and tissue section preparation**

Freshly excised tumors, tumor-draining lymph nodes (TDLNs), or spleens were rinsed in ice-cold PBS and minced into ~1–2 mm fragments using sterile scissors. The tissue fragments were transferred into RPMI-1640 medium supplemented with collagenase type IV (1 μg mL⁻¹) and DNase I (150 μg mL⁻¹), then digested at 37 °C for 45 min on an orbital shaker with gentle agitation. The digested tissue was triturated and filtered through a 40 μm cell strainer to obtain a single-cell suspension. Red blood cells (RBCs) were lysed using 1× RBC lysis buffer for 3 min at room temperature. The suspension was immediately neutralized with excess PBS, centrifuged, and washed twice with PBS. The final cell pellet was resuspended in RPMI-1640 containing 2% FBS and 2 mM EDTA, and maintained on ice for downstream applications, including flow cytometry and intracellular cytokine staining.

For histological and immunofluorescence analyses, tissues were fixed in 10% neutral-buffered formalin for 24 h, dehydrated through graded ethanol, cleared in xylene, and embedded in paraffin. Paraffin blocks were stored at −20 °C until sectioning. Sections (5 μm thick) were prepared using a microtome (Leica Biosystems, Seoul, South Korea) and mounted onto charged glass slides. Prior to staining, sections were deparaffinized in xylene and rehydrated through a graded ethanol series into distilled water. Hematoxylin and eosin (H&E) staining was performed using standard protocols. For immunofluorescence, antigen retrieval was conducted in citrate buffer (pH 6.0), followed by permeabilization with 0.1% Triton X-100 and blocking with 5% BSA. Ki-67 immunofluorescence staining was performed on tumor sections using Alexa Fluor® 594-conjugated anti-Ki-67 antibodies (BioLegend, San Diego, CA, USA), followed by nuclear counterstaining with DAPI in antifade mounting medium. Apoptotic cells were detected by terminal deoxynucleotidyl transferase dUTP nick-end labeling (TUNEL) using a commercial kit (Abcam, Cambridge, UK), according to the manufacturer’s instructions. Fluorescence images were acquired using CLSM.

**Dendritic cell maturation in tumor-draining lymph nodes**

Tumor-draining lymph nodes (TDLNs) were harvested, stained with Fixable Viability Dye eFluor™ 450 (Thermo Fisher Scientific, Waltham, MA, USA; 1:1000 dilution) for 30 min at 4 °C in the dark, and preincubated with anti-CD16/32 (Fc block) for 10 min on ice to minimize nonspecific antibody binding. Cells were subsequently stained for 45 min at 4 °C in the dark using a dendritic cell (DC) panel comprising anti-CD11c-PE, anti-CD80-PE, and anti-CD86-FITC antibodies (BioLegend, San Diego, CA, USA; 1:80 dilution for each). Following staining, samples were analyzed on a BD FACSAria™ Fusion cytometer (BD Biosciences, USA) and processed using FlowJo software. A standard gating strategy was employed, after which CD11c^+^ events were selected for DC analysis. Mature DCs (mDCs) were defined as the CD11c^+^CD80^+^CD86^+^ population.

***In vivo* CD4^+^ and CD8^+^ T cell infiltration**

Tumor-infiltrating leukocytes (TILs) were stained with Fixable Viability Dye eFluor™ 450 (Thermo Fisher Scientific, Waltham, MA, USA; 1:1000 dilution) for 30 min at 4 °C in the dark, resuspended in staining buffer, and incubated with anti-CD16/32 (Fc-block) for 15 min at 4 °C to prevent nonspecific binding. Cells were then stained for 45 min at 4 °C (protected from light) with an antibody cocktail consisting of anti-CD3-APC, anti-CD4-FITC, and anti-CD8-PE/Cy7 (BioLegend, San Diego, CA, USA; 1:80 dilution each). Following three PBS washes, samples were analyzed using a BD FACSAria™ Fusion flow cytometer (BD Biosciences, USA) and data were processed with FlowJo software. CD3^+^ T cells were selected after sequential gating on debris exclusion, singlets, and live cells, and within this population, CD4^+^ and CD8^+^ T-cell subsets were quantified.

***In vivo* cytokine production**

***Intratumoral cytokine profiling (intracellular staining)***. Tumor-infiltrating leukocytes (TILs) were stimulated for 4 h at 37 °C with PMA (50 ng mL⁻^1^), ionomycin (1 μg mL⁻^1^), and GolgiStop (10 μg mL⁻^1^) to facilitate intracellular cytokine accumulation. Following stimulation, cells were stained with Fixable Viability Dye eFluor™ 450 (Thermo Fisher Scientific, Waltham, MA, USA; 1:1000 dilution) for 30 min at 4 °C in the dark. Next, Fc receptors were blocked using anti-CD16/32 (15 min, 4 °C), and cells were surface-stained with anti-CD3-APC, anti-CD4-FITC, and anti-CD8α-PE/Cy7 antibodies (BioLegend, San Diego, CA, USA; 1:80 dilution each) for 45 min at 4 °C, protected from light. Cells were then fixed and permeabilized using BD Cytofix/Cytoperm according to the manufacturer’s instructions and stained intracellularly with anti-Granzyme B-PerCP/Cy5.5, anti-Perforin-PE, anti-IL-10-PerCP/Cy5.5, and anti-IL-17A-APC/Cy7 (BioLegend; 1:80 dilution each). After two washes with Cytoperm/Cytowash buffer, samples were acquired using a BD FACSAria™ Fusion flow cytometer (BD Biosciences, USA) with standard compensation controls. Data were gated sequentially on lymphocytes (FSC/SSC) → singlets → live cells → CD3^+^ T cells, and cytokine-producing subsets were quantified within CD4^+^ and CD8^+^ T-cell populations.

***Serum cytokine quantification (ELISA).*** Whole blood was collected and allowed to clot at room temperature for 20 min, followed by centrifugation at 3000 rpm for 15 min to obtain serum. Serum levels of IL-6, IFN-γ, TNF-α, and IL-10 were measured using commercial ELISA kits (Mouse IL-6, IFN-γ, TNF-α, and IL-10 ELISA sets; BioLegend, San Diego, CA, USA), following the manufacturers’ protocols.

***In vitro* tumor cell killing assay**

To assess the tumor-selective cytotoxicity of SHINE-induced T cells, CD8^+^ T cells were isolated from the tumor of 4T1 tumor-bearing mice treated with SHINE using a MojoSort™ Mouse CD8 T Cell Isolation Kit based on negative selection, according to the manufacturer’s protocol (BioLegend, San Diego, CA, USA). 4T1 tumor cells or 3T3 normal cells were seeded into 96-well plates at a density of 6 × 10^3^ cells per well and allowed to attach overnight. The isolated CD8^+^ T cells were then added at the indicated effector-to-target (E:T) ratios and co-incubated with target cells for 48 h. After co-incubation, target cell viability was determined using a Cell Counting Kit-8 (CCK-8) assay.

**2. Additional data**


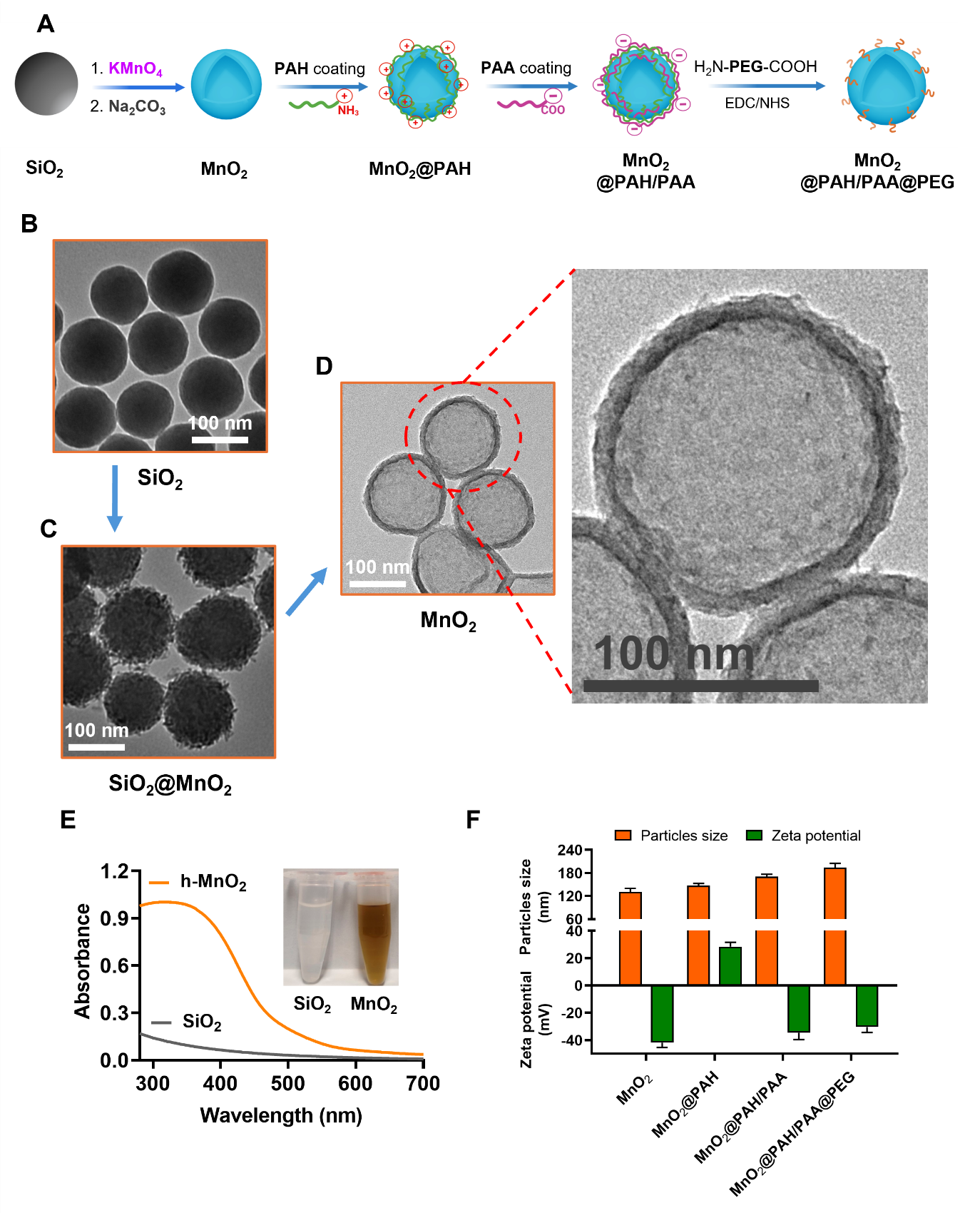


**Figure S1**. **Synthesis and surface modification of h-MnO_2_ nanoparticles (NPs**). (**A**) Synthesis scheme of MnO_2_@PAH/PAA@PEG NPs. SiO_2_ NPs were used as hard templates to form a MnO_2_ shell *via* KMnO_4_ reduction. The SiO_2_ core was then removed with Na_2_CO_3_. The resulting hollow MnO_2_ NPs were sequentially modified with PAH and PAA polymers using a layer-by-layer method. Finally, H_2_N-PEG-COOH was covalently conjugated to the surface through EDC/NHS chemistry. (**B**, **C**, **D**) TEM images of SiO_2_, SiO_2_@MnO_2_, and MnO_2_ NPs, respectively. (**E**) UV-vis spectra of SiO_2_ NPs and reaction products indicate successful formation of MnO_2_. (**F**) Changes in zeta potential and hydrodynamic particle sizes during the surface modification of MnO_2_ NPs confirm the success of the modification process.


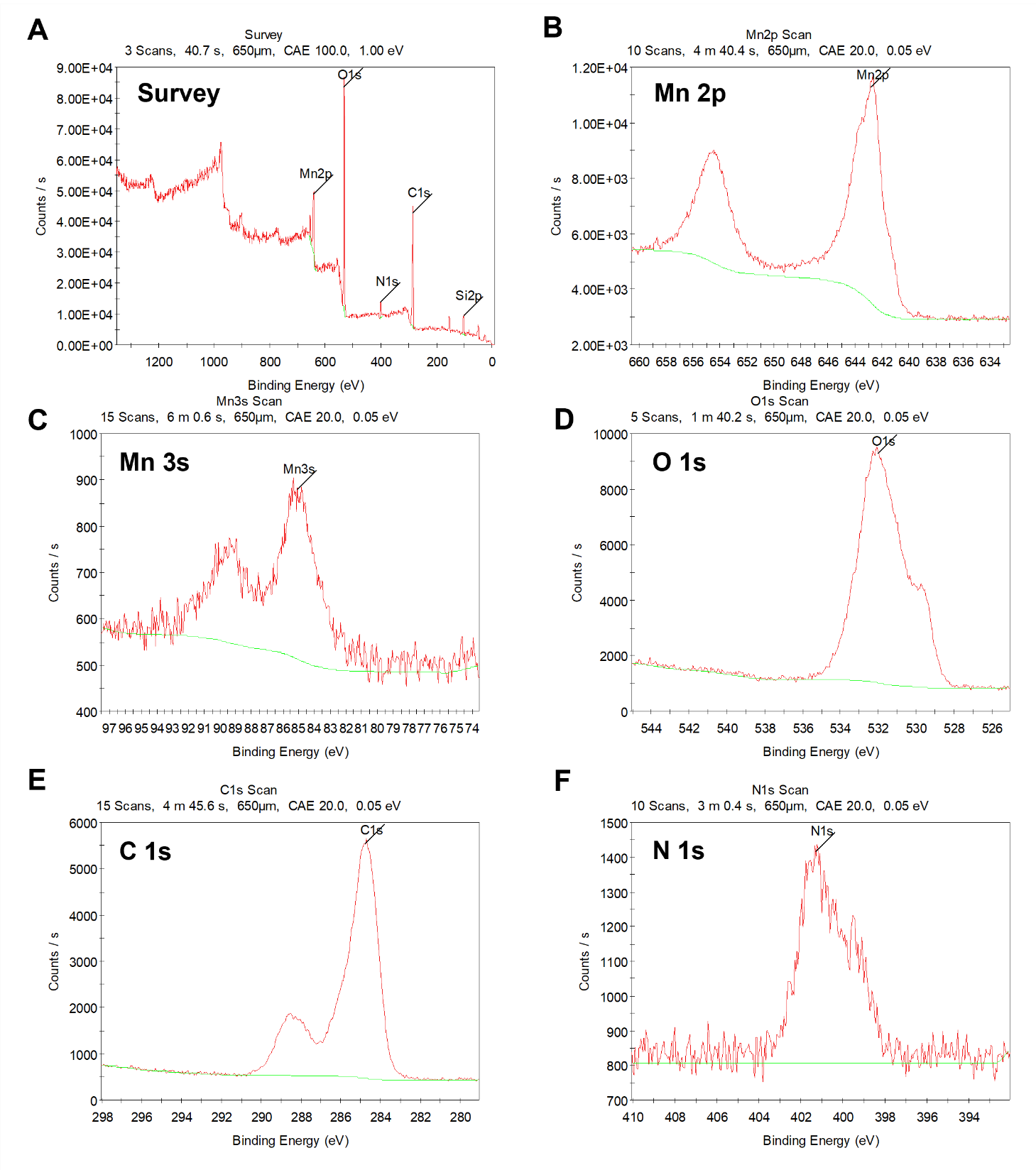


**Figure S2**. **XPS characterization of SHINE**. (**A**) XPS survey spectrum and high-resolution spectra of (**B**) Mn 2p, (**C**) Mn 3s, (**D**) O 1s, (**E**) C 1s, and (**F**) N 1s.


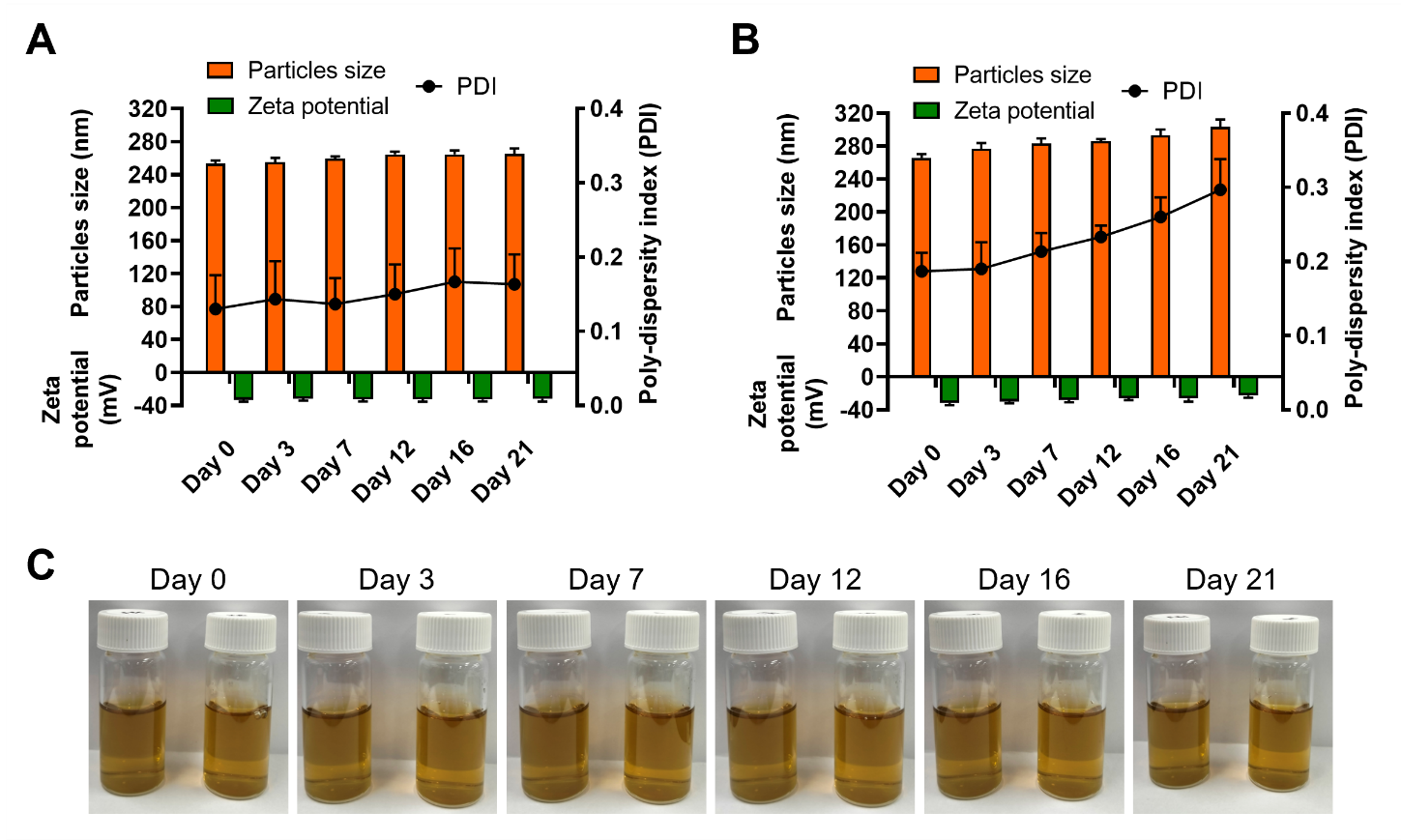


**Figure S3**. Long-term colloidal stability of SHINE (1 mg mL^-1^) dispersed in (**A**) H_2_O and (**B**) 10% FBS over 21 days, as assessed by hydrodynamic size, polydispersity index (PDI), and zeta potential. (**C**) Digital photographs of SHINE dispersed in H_2_O (left) or 10% FBS (right) at the indicated time points.


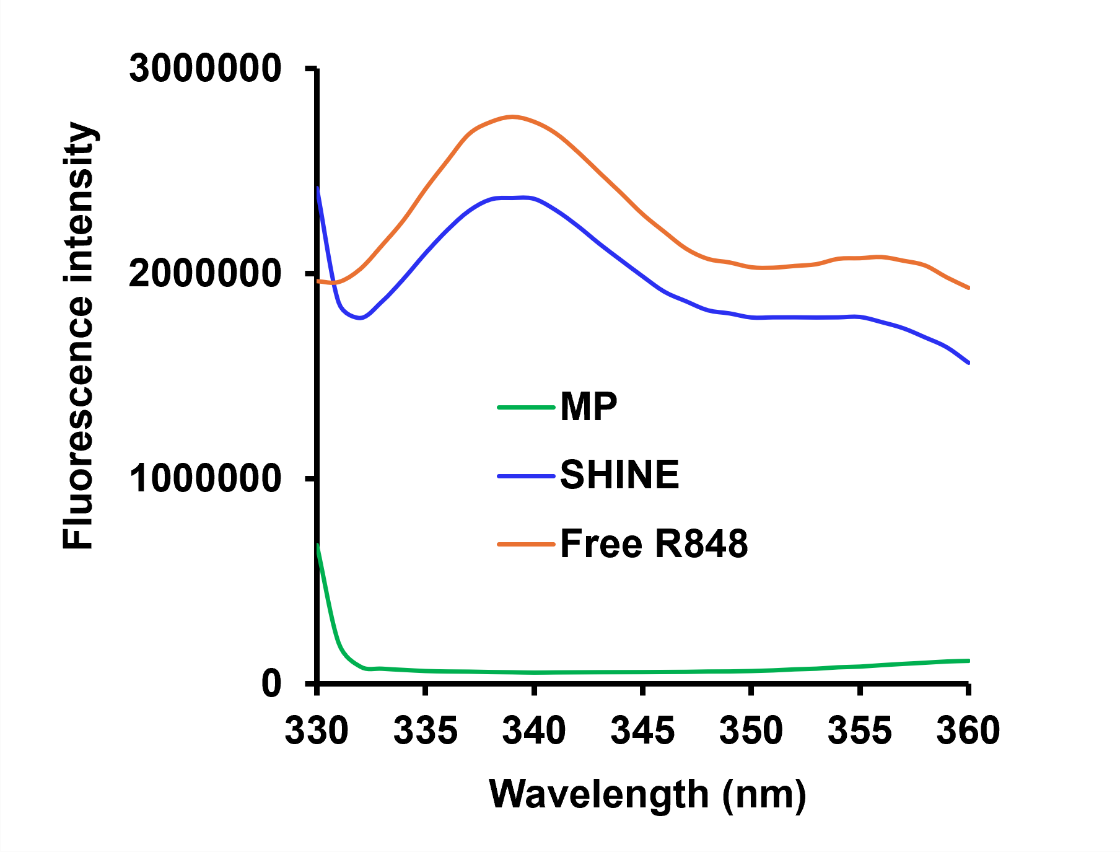


**Figure S4**. Fluorescence emission spectra of MP (MnO_2_@aPD-L1 NPs), SHINE (MnO_2_@R848@aPD-L1 NPs), and free R848. Measurement conditions: solvent, H_2_O; excitation wavelength, λ_ex_: 323 nm; emission range: 330–360 nm; slit width: 5 nm.

MP exhibited no detectable emission within the scanned range, whereas SHINE showed a pronounced emission peak at 339 nm that matched free R848, confirming successful R848 loading into the MnO_2_ NPs.


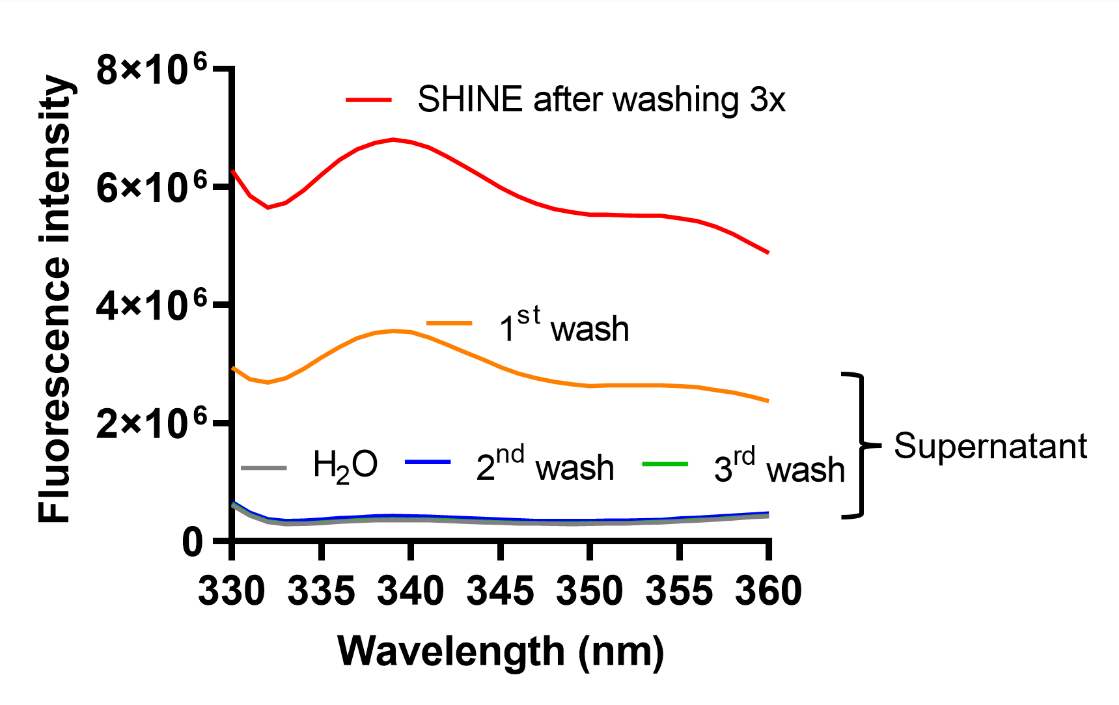


**Figure S5**. Fluorescence emission spectra of SHINE and the corresponding wash supernatants collected after three washing steps. Measurement conditions: solvent, H_2_O; excitation wavelength, λ_ex_: 323 nm; emission range: 330–360 nm; slit width: 5 nm.


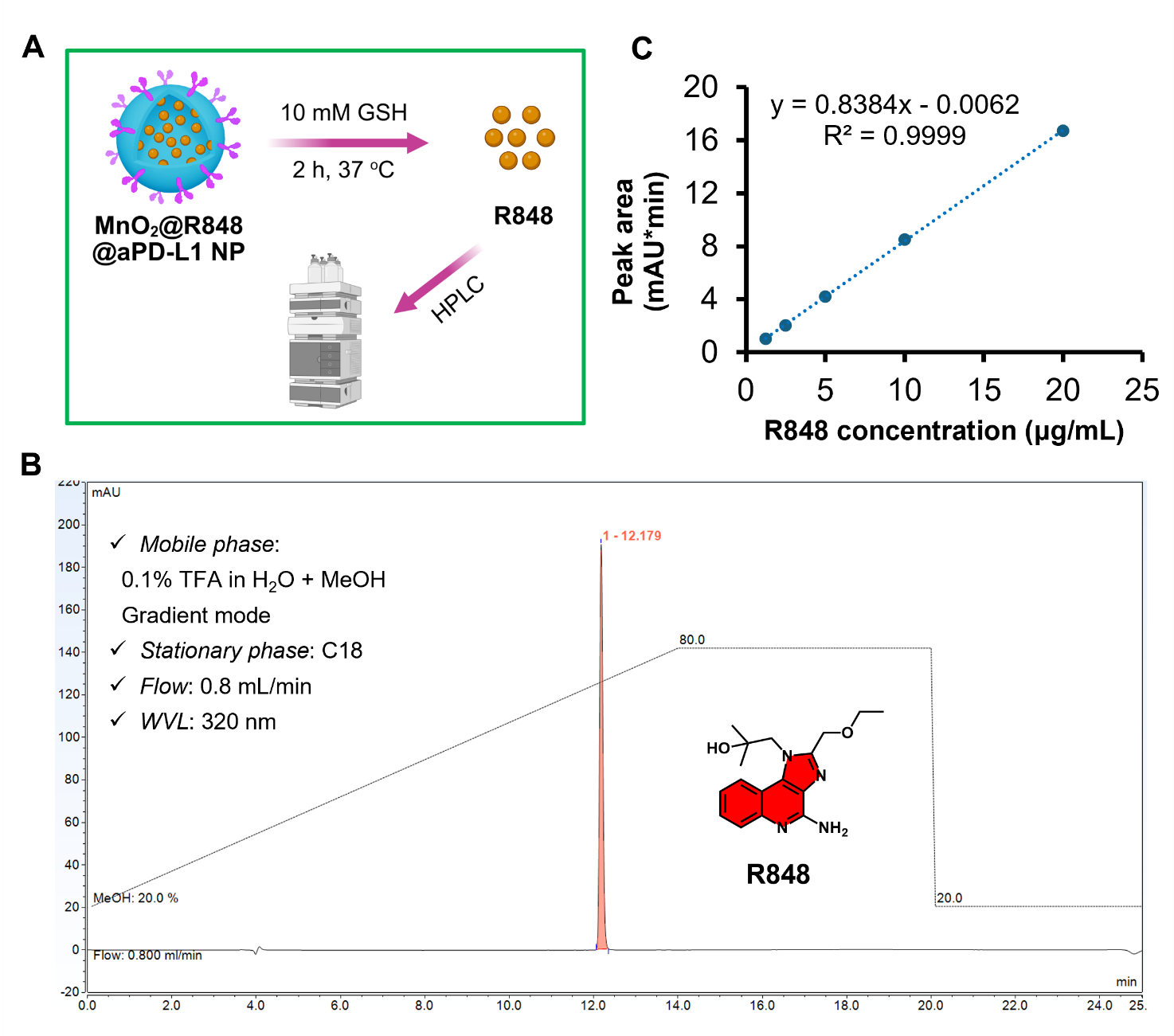


**Figure S6**. **Quantification of R848 by HPLC**. (**A**) Schematic of the experimental procedure for R848 release and detection. (**B**) HPLC parameters for R848 analysis. (**C**) Standard calibration curve for R848 quantification.


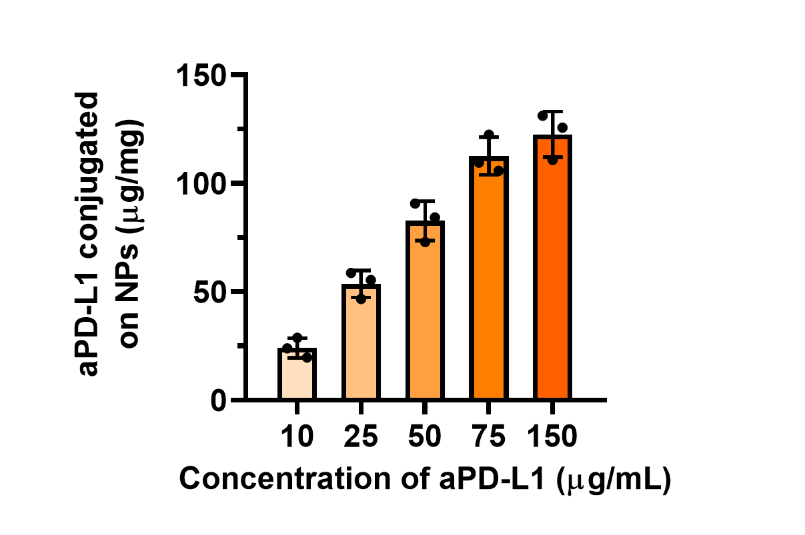


**Figure S7**. Amount of surface-conjugated aPD-L1 on SHINE nanoparticles as a function of the initial aPD-L1 concentration. The amount of conjugated aPD-L1 was quantified by protein assay and expressed as μg aPD-L1 per mg SHINE nanoparticles. Data are presented as mean ± SD (*n = 3*).


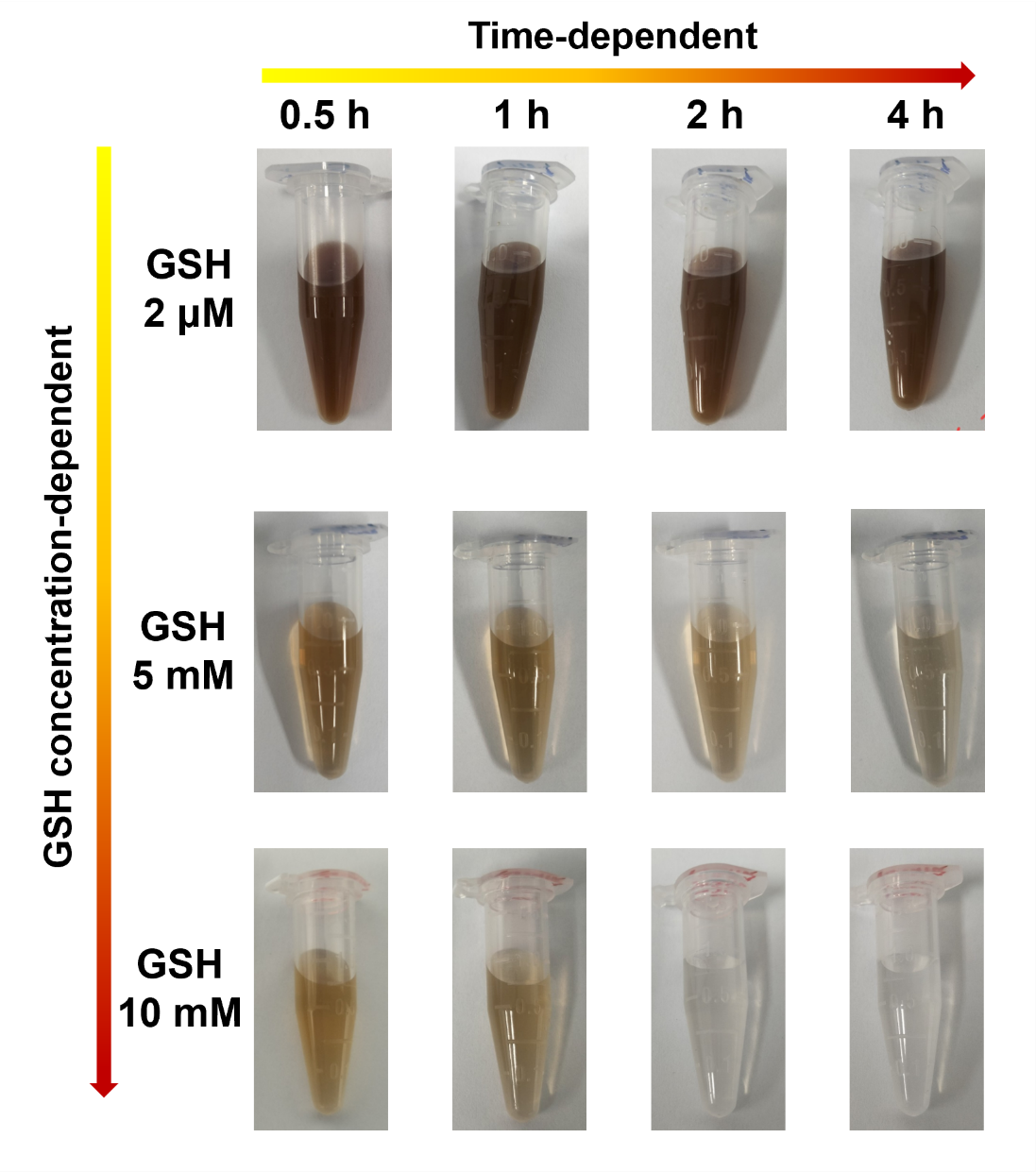


**Figure S8**. **Visual assessment of GSH-triggered biodegradation of SHINE under varying reductive conditions**. SHINE was incubated in PBS (pH 7.4) containing 2 μM, 5 mM, or 10 mM GSH at 37 °C. Samples were photographed at different time points (0.5, 1, 2, and 4 h). A concentration- and time-dependent discoloration of the SHINE suspension was observed, with rapid fading of color in higher GSH concentrations, indicating accelerated MnO_2_ shell degradation and dissolution.


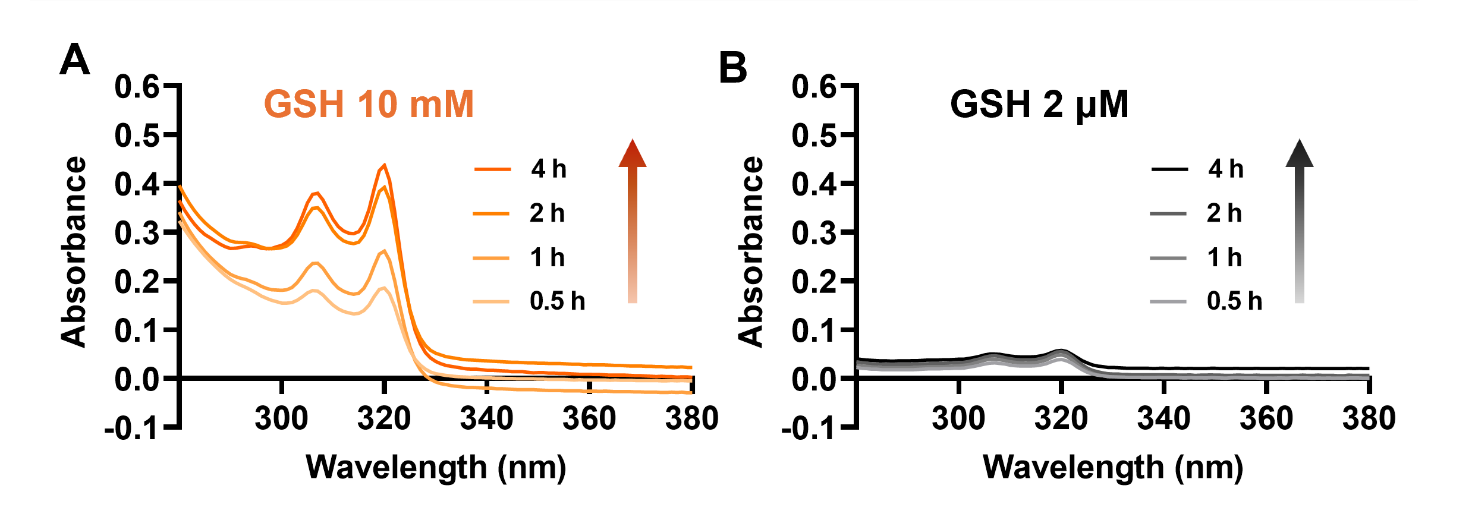


**Figure S9**. Controlled release of R848 from SHINE in PBS (pH 7.4) containing (**A**) 10 mM GSH or (**B**) 2 μM GSH at 37 °C over time (0.5, 1, 2, and 4 h), as measured by absorbance. At 10 mM GSH, SHINE underwent structural disassembly, leading to R848 release.


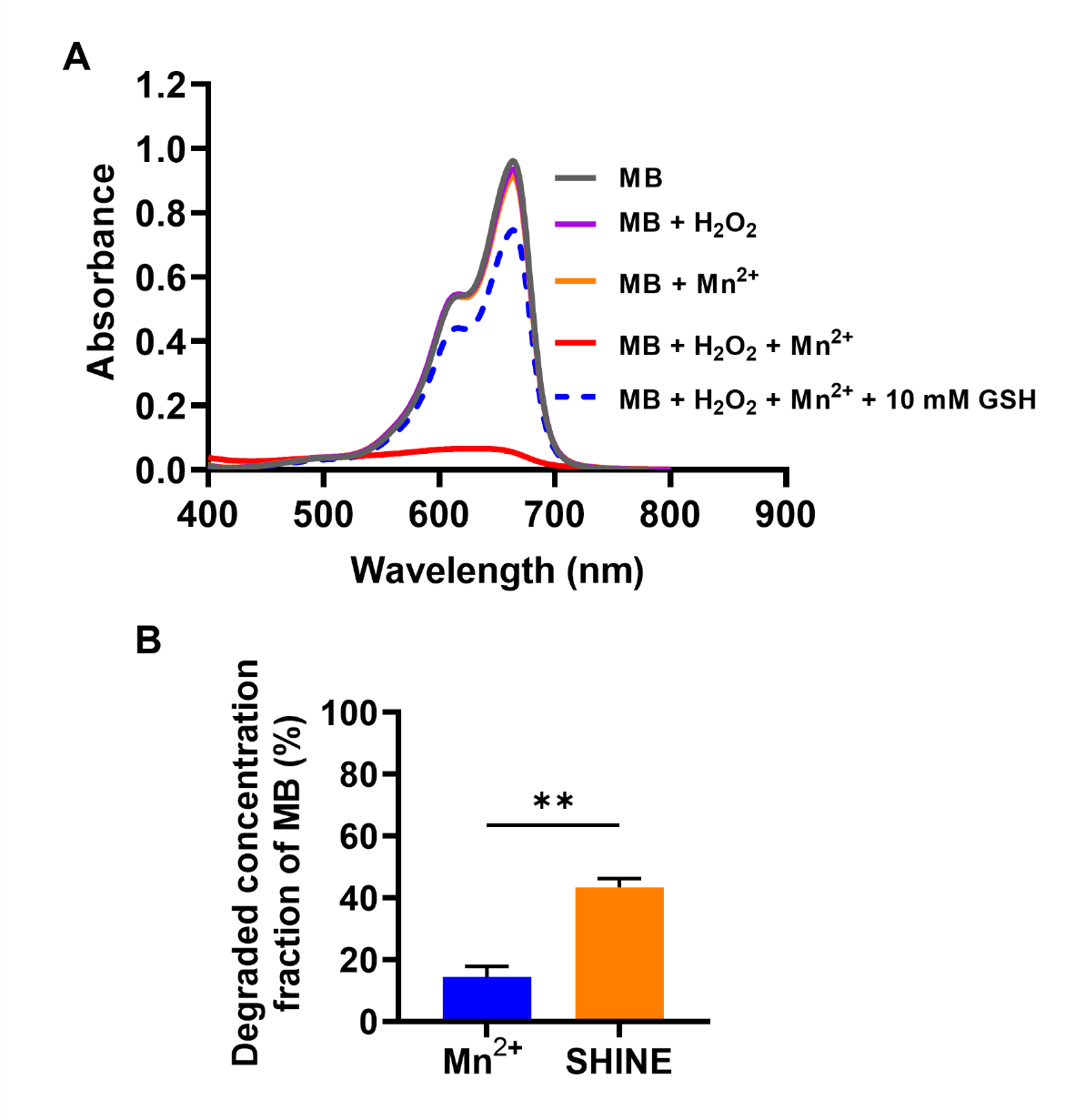


**Figure S10**. (**A**) UV/vis absorption spectra of MB after treatment with H_2_O_2_, MnCl_2_ alone, or (H_2_O_2_ + MnCl_2_) combination with and without GSH. (**B**) Degradation percent of MB by Mn^2+^ or SHINE in the presence of 10 mM GSH. Reaction condition: 25 mM NaHCO_3_/5% CO_2_, [Mn] = 100 μg mL^-1^, [H_2_O_2_] = 8 mM. Data are presented as mean ± SD (*n = 3*), ***p* < 0.01.


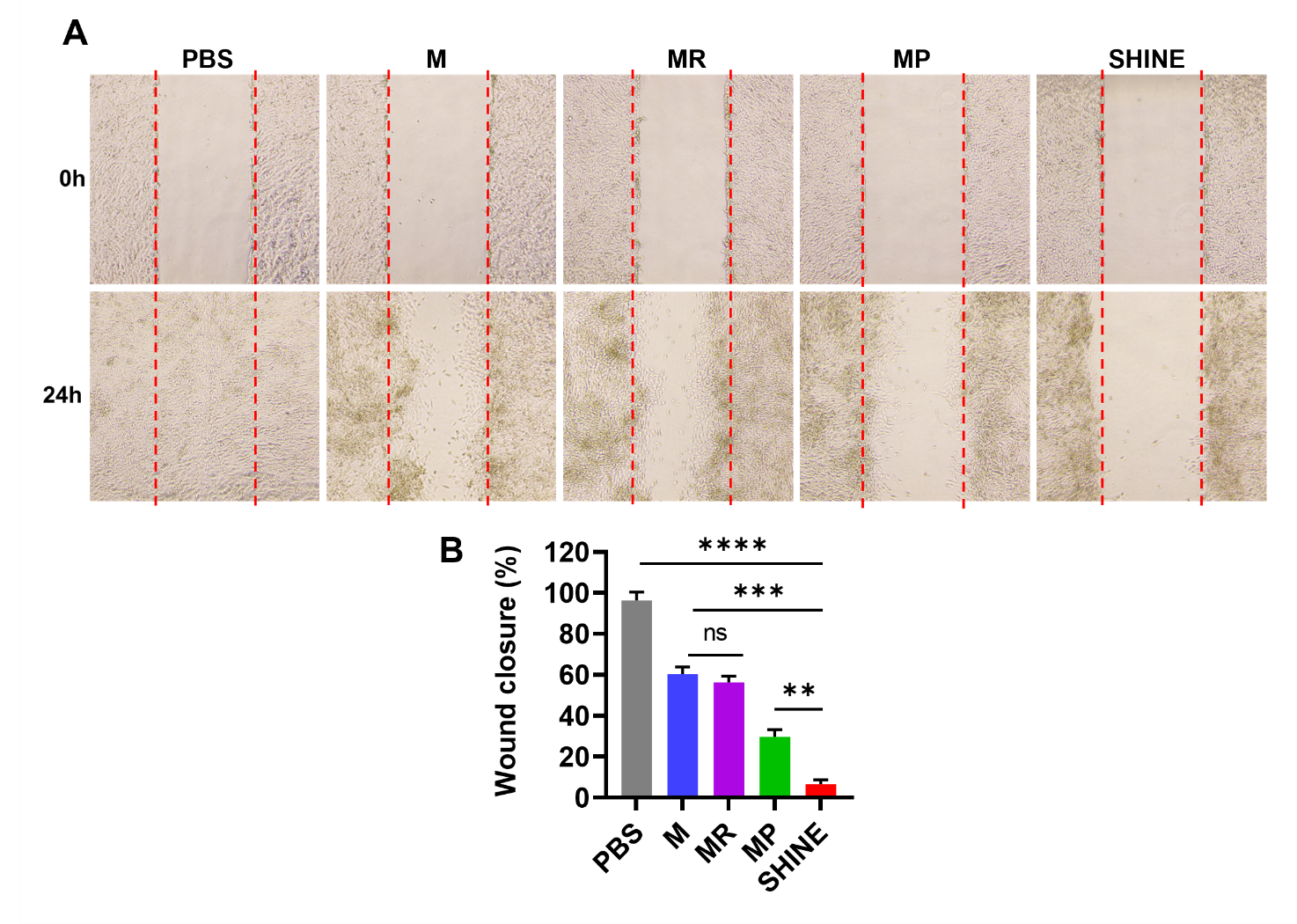


**Figure S11**. **Wound healing assay evaluating the anti-metastatic effect of SHINE on 4T1 cells**. (**A**) Representative photos and (**B**) quantitative analysis of relative wound closure. Data are presented as mean ± SD (*n = 3*). ***p* < 0.01, ****p* < 0.001, *****p* < 0.0001; n.s., not significant (*p* > 0.05).


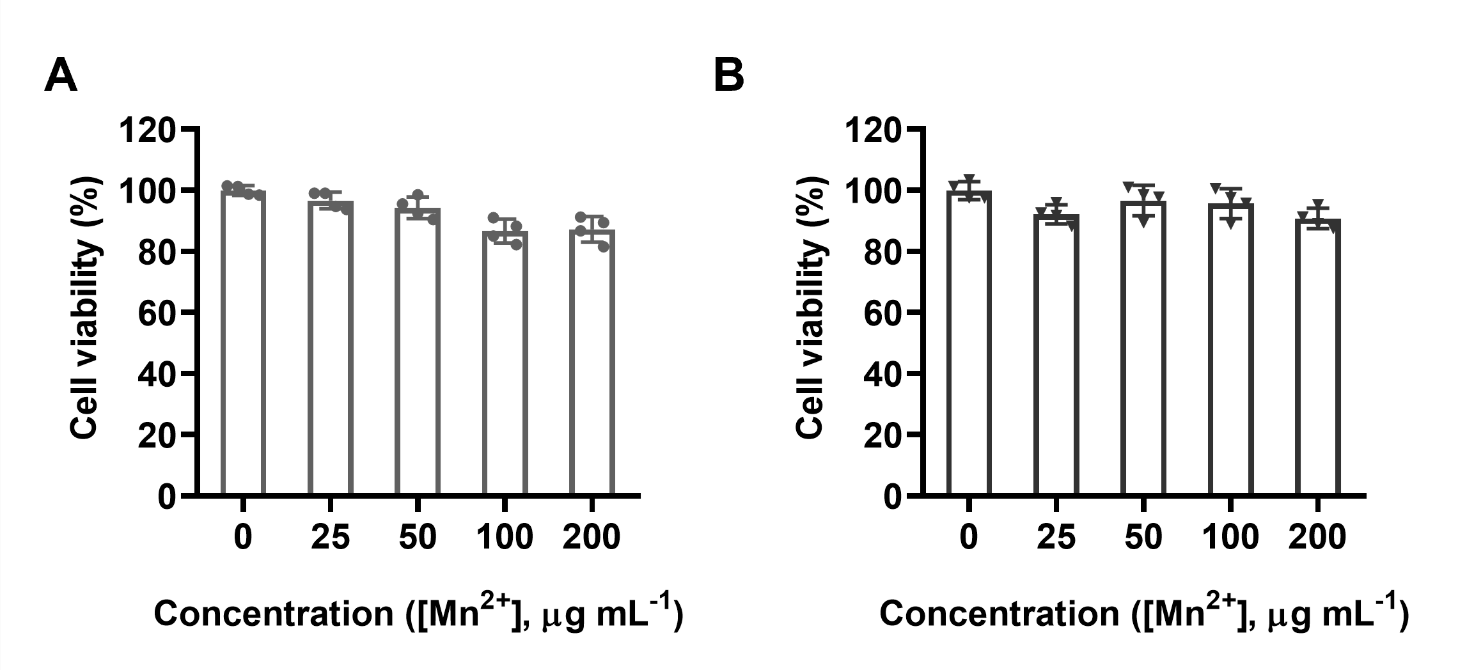


**Figure S12.** Viability of (**A**) BMDCs and (**B**) CD8^+^ T cells after 24 h treatment with SHINE (Mn-equivalent concentration: 0–200 μg mL^-1^). Data are presented as mean ± SD (*n = 4*).


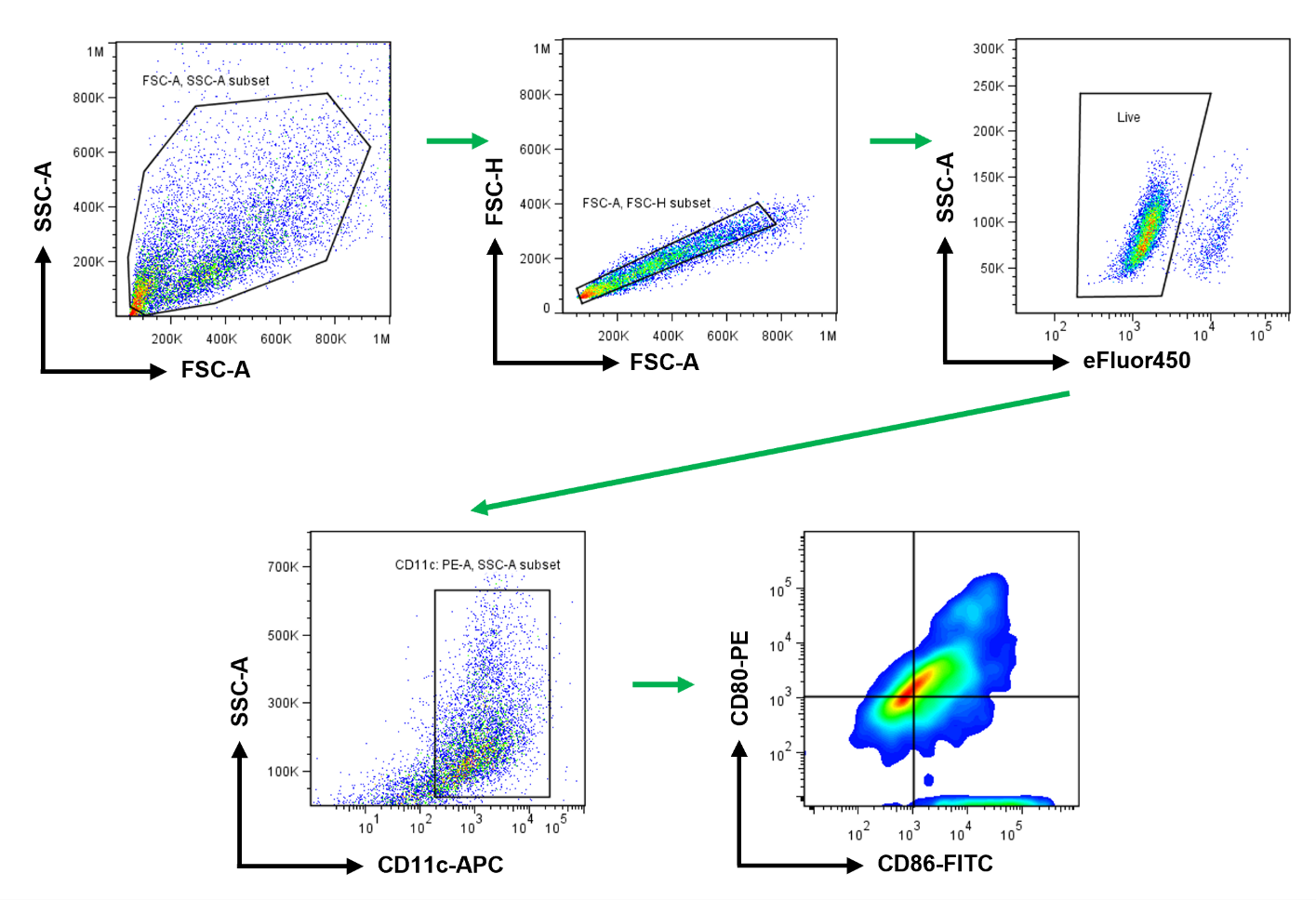


**Figure S13.** Gating strategy for *in vitro* identification of mature dendritic cells (CD11c^+^D80^+^D86^+^).


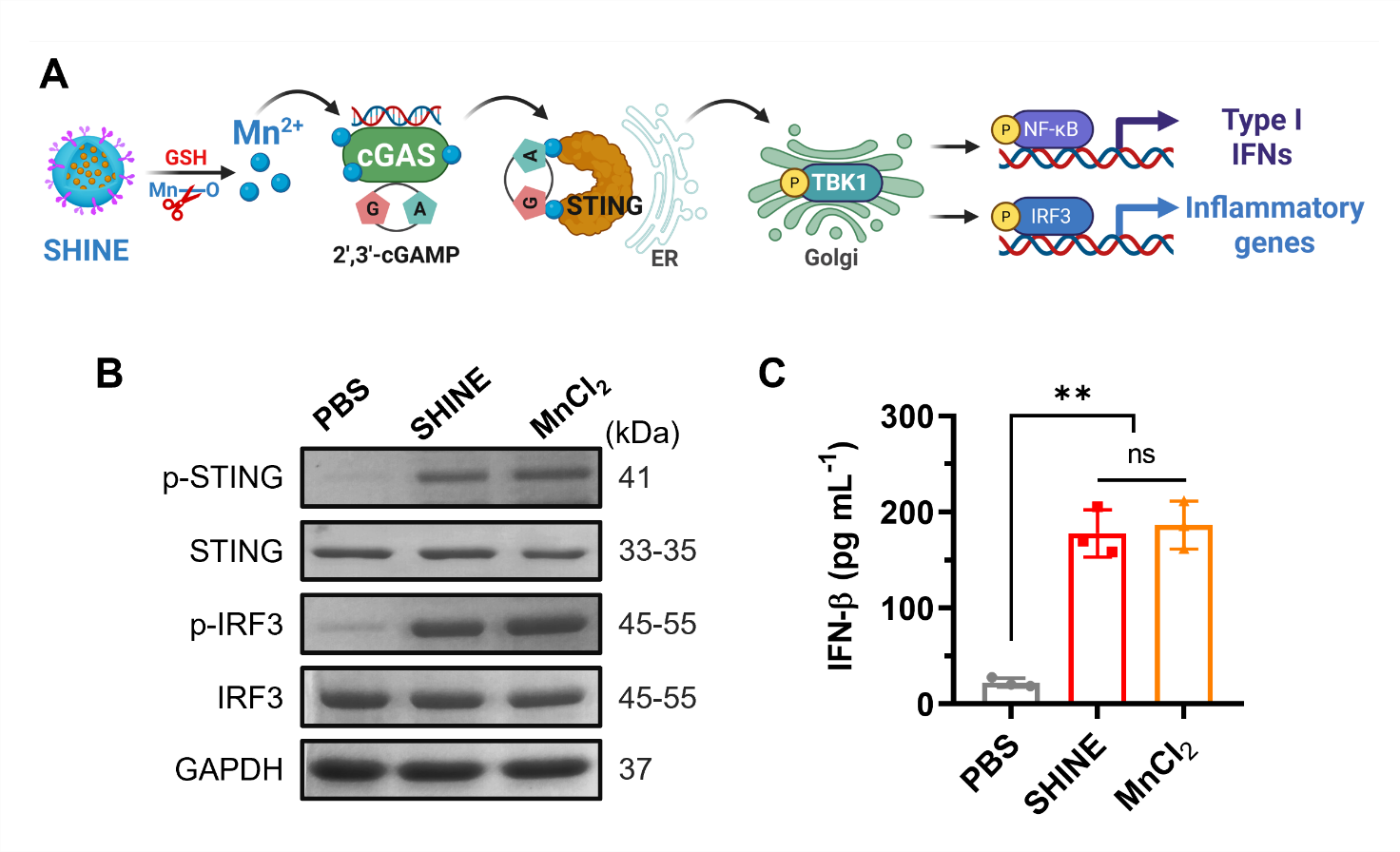


**Figure S14**. SHINE-derived Mn^2+^ activates cGAS-STING signaling *in vitro*. (**A**) Schematic illustration of Mn^2+^-triggered cGAS-STING pathway activation. (**B**) Western blot analysis of STING, p-STING, IRF3, and p-IRF3 in mouse BMDCs following the indicated treatments. (**C**) ELISA quantification of IFN-β secretion by BMDCs. Data are presented as mean ± SD (*n = 3*). ***p* < 0.01.


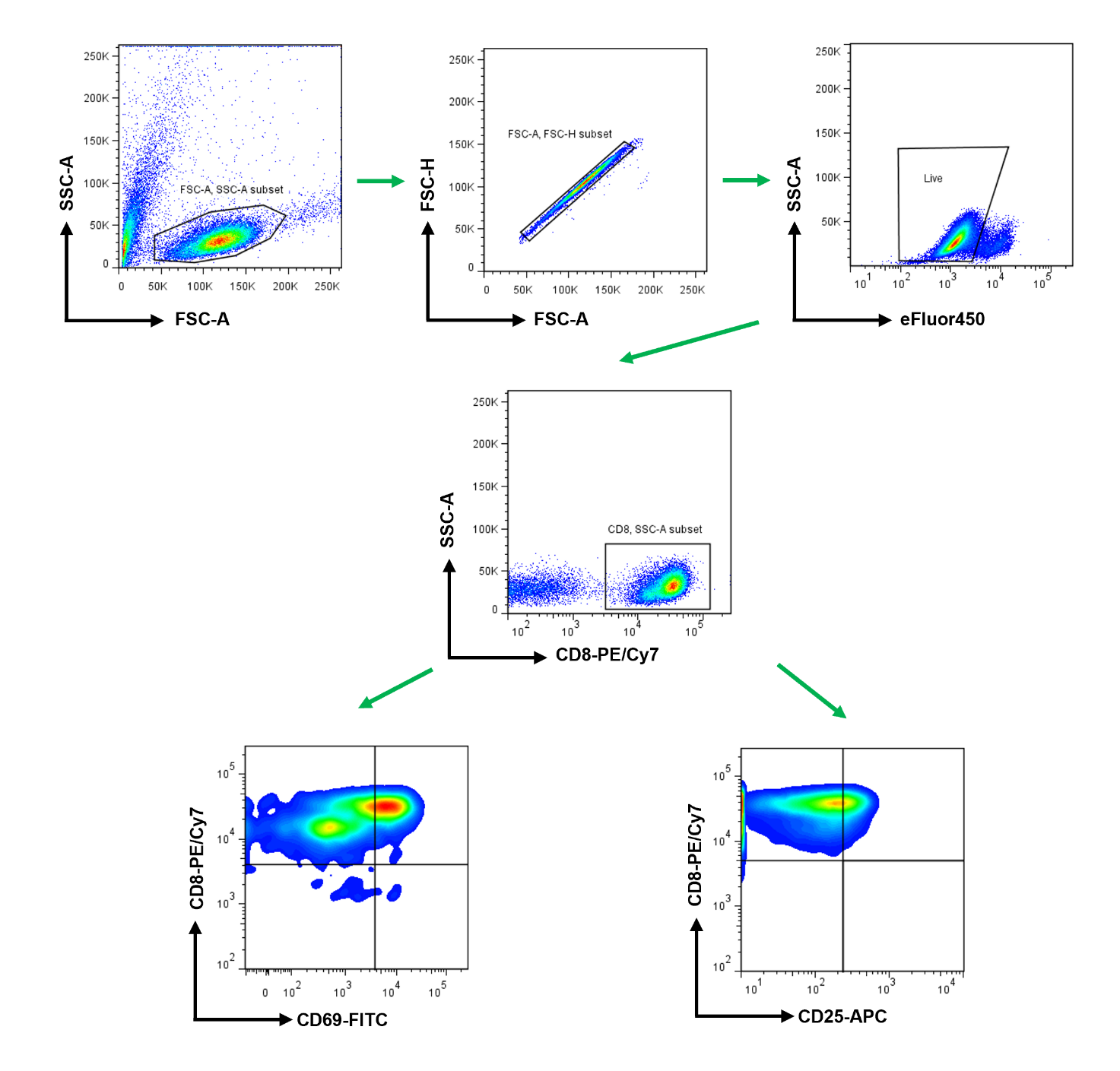


**Figure S15**. Gating strategy for *in vitro* identification of CD69^+^ and CD25^+^ T cells.


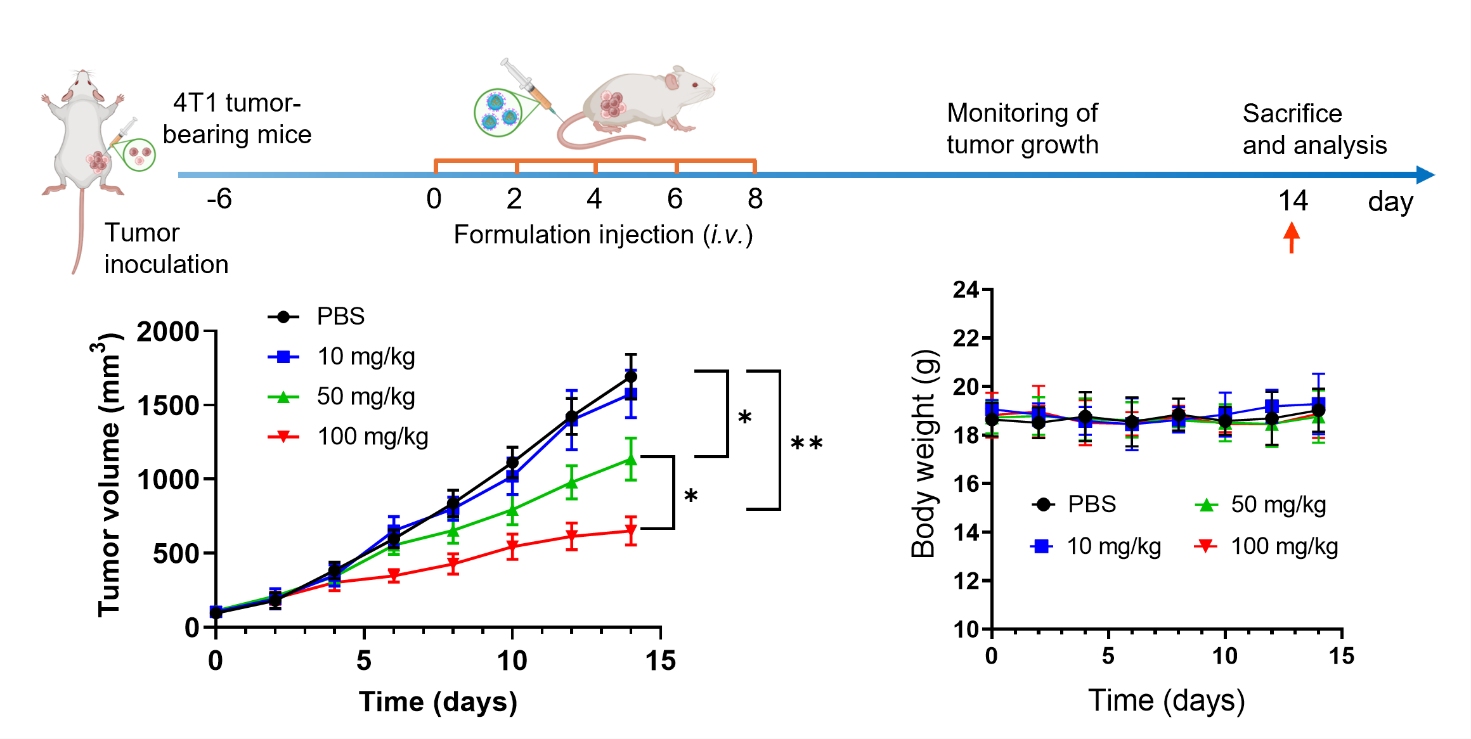


**Figure S16**. **Preliminary evaluation of the anti-tumor efficacy of SHINE nanovaccine.**

A 4T1 tumor model was established by subcutaneous injection of 1 × 10^6^ 4T1 cells suspended in 100 μL of FBS-free DMEM and Matrigel (1:1, v/v) into the right flank of female BALB/c mice (6–8 weeks old, 18–20 g). When tumors reached ~100 mm^3^ (designated as day 0), mice were randomized into four groups (*n = 5* per group) and administered intravenous injections of SHINE *via* the tail vein on days 0, 2, 4, 6, and 8 (200 μL per dose, five doses total). SHINE was tested at doses of 10, 50, or 100 mg kg⁻^1^. Tumor volumes were measured every two days using digital calipers, and body weights were monitored throughout to assess systemic toxicity. Based on these preliminary results, the 100 mg kg⁻^1^ dose was selected for subsequent studies. Data are presented as mean ± SD (*n = 5*). **p* < 0.05, ***p* < 0.01.


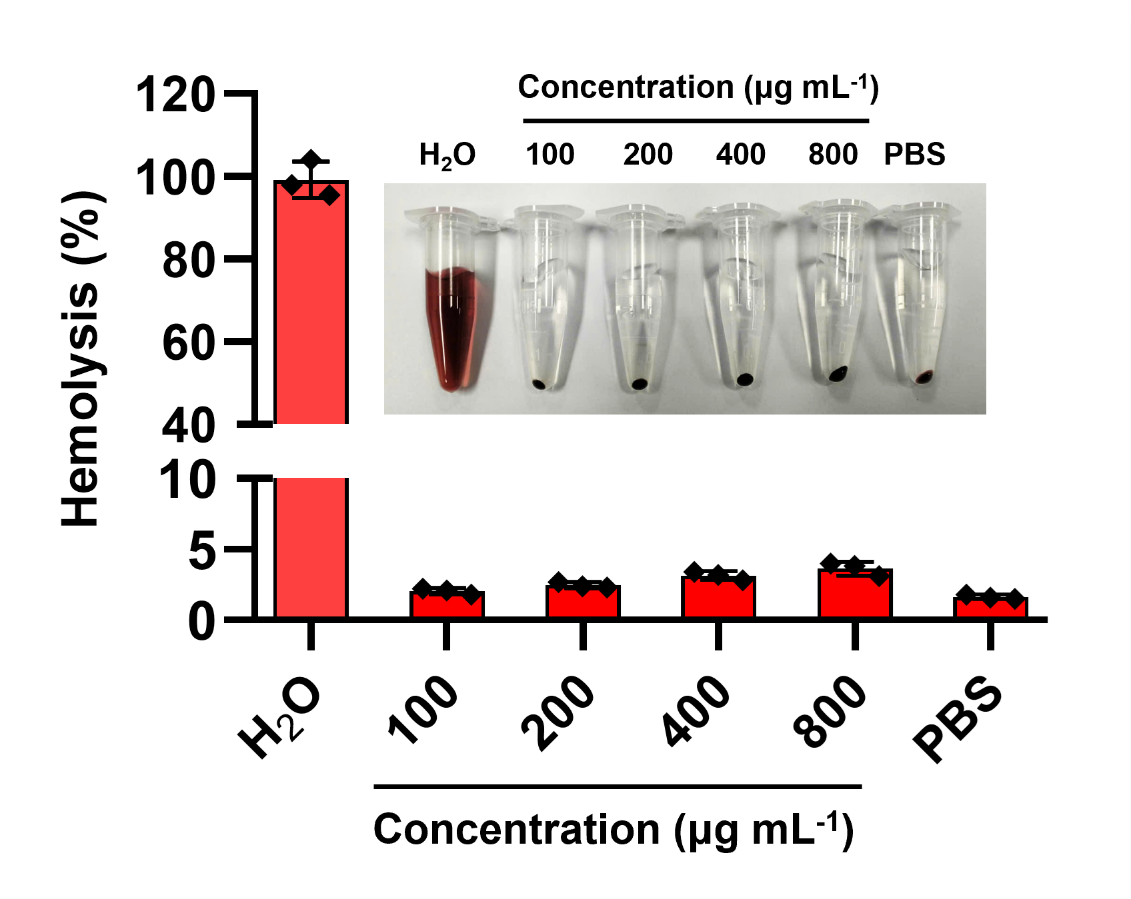


**Figure S17**. **Hemolysis assessment of SHINE nanovaccine.** Mouse red blood cells (RBCs) treated with SHINE at various Mn-equivalent concentrations (100, 200, 400, and 800 μg mL^-1^). RBCs incubated with deionized water and PBS served as positive and negative controls, respectively. Inset: images of the supernatants after 5 h incubation at 37 °C followed by centrifugation (14,000 rpm, 10 min).


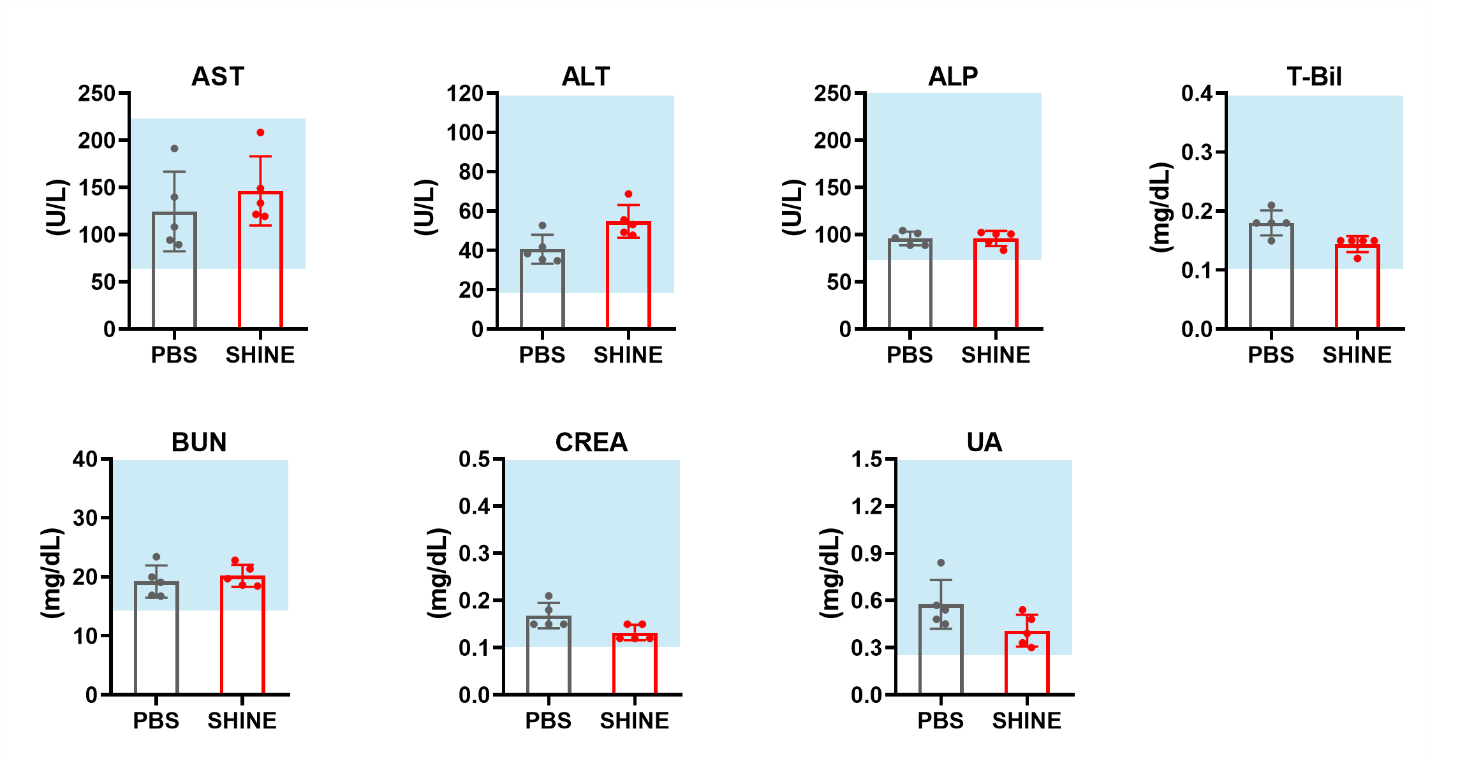


**Figure S18**. Serum biochemistry analysis of liver function markers (AST, ALT, ALP, and T-Bil) and kidney function markers (UA, CREA, and BUN) on day 20 after intravenous injection. AST, aspartate transaminase; ALT, alanine transaminase; ALP, alkaline phosphatase; T-Bil, total bilirubin; UA, uric acid; CREA, creatinine; BUN, blood urea nitrogen. Data are presented as mean ± SD (*n = 5*).


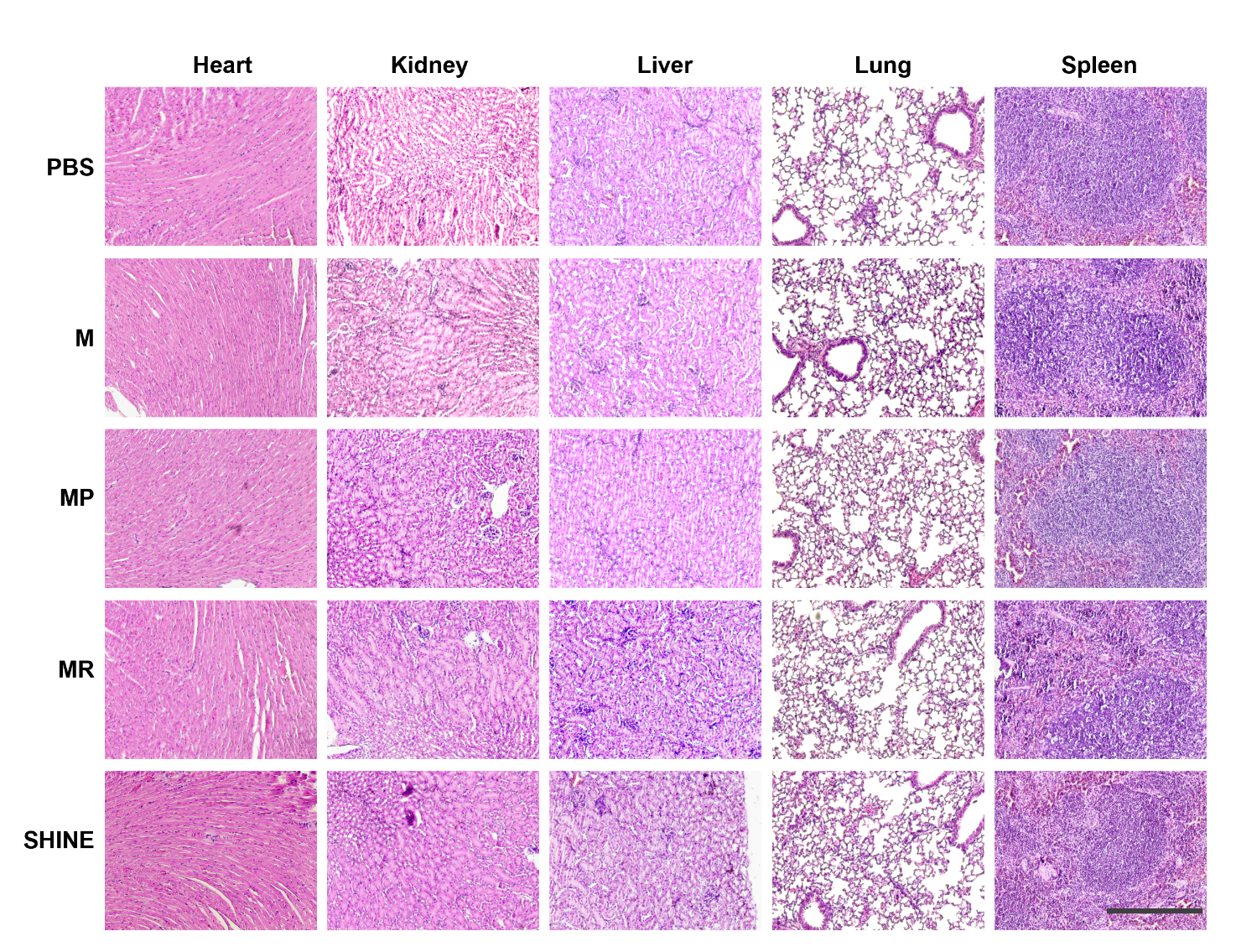


**Figure S19**. Representative hematoxylin and eosin (H&E)-stained sections of major organs (heart, kidney, liver, lung, and spleen) on day 20 after the indicated treatments. Scale bar, 200 μm.


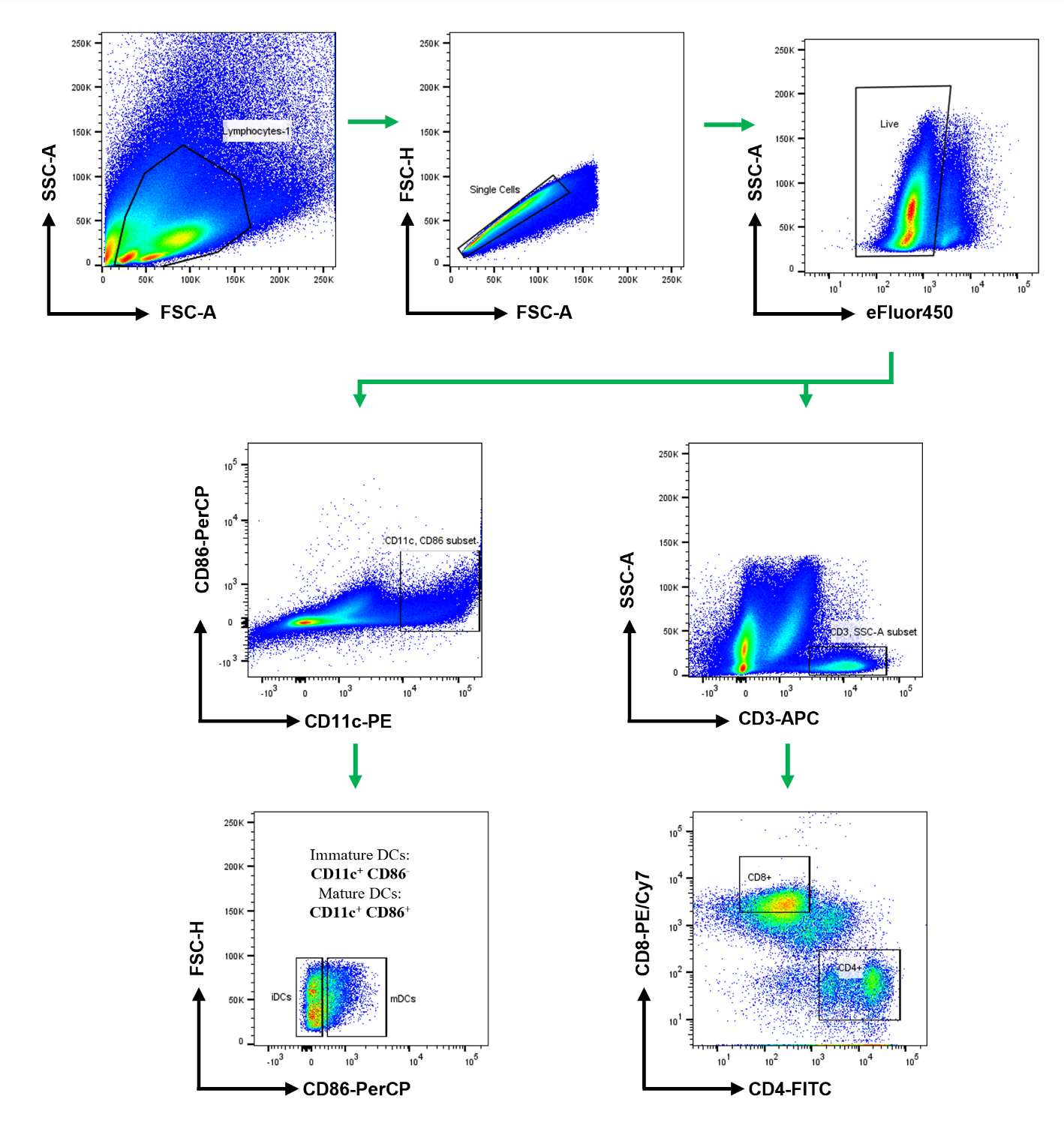


**Figure S20**. Gating strategy for isolating immature DCs, mature DCs, helper CD4^+^ T cells, and cytotoxic CD8^+^ T cells in tumors.


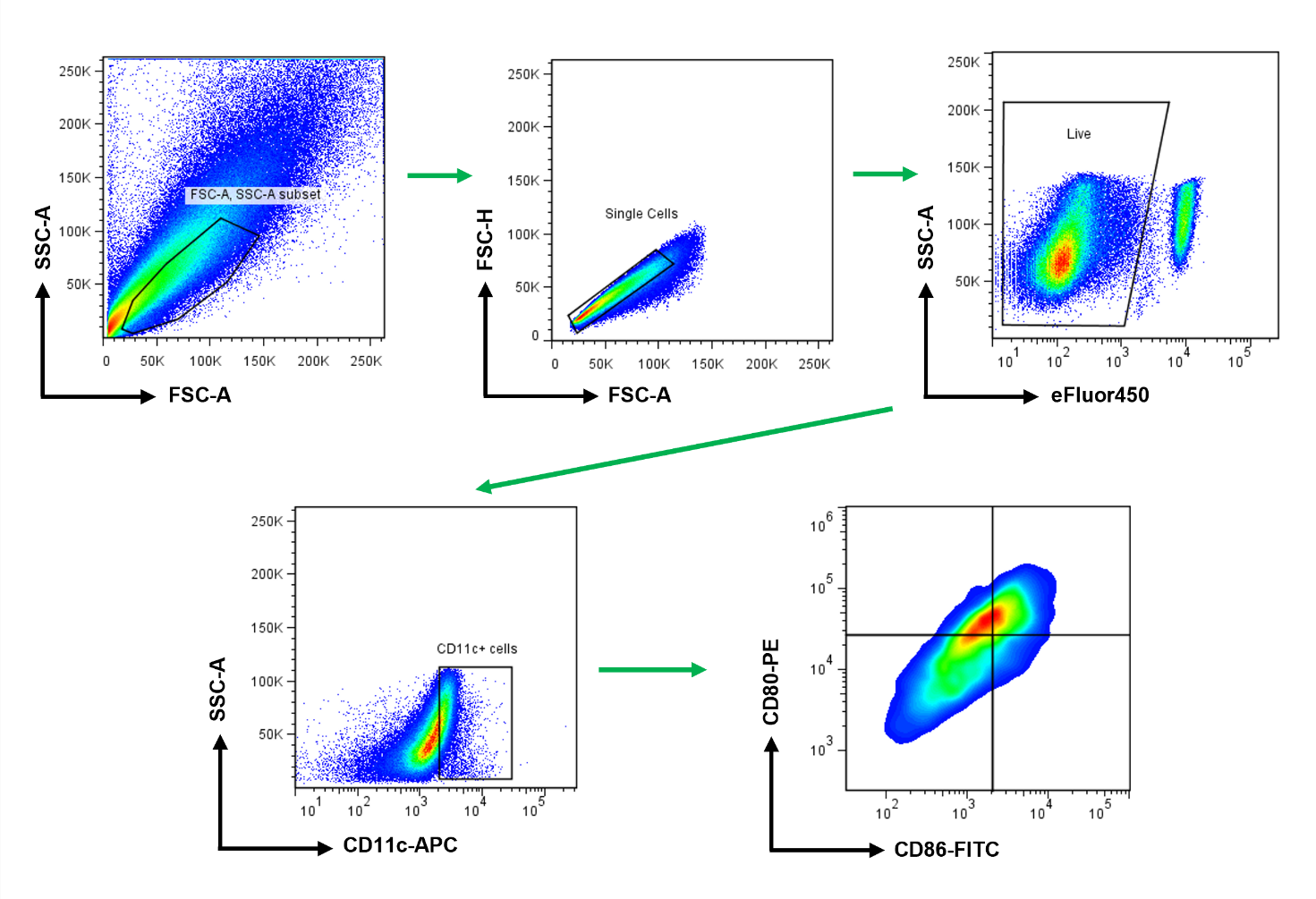


**Figure S21**. Gating strategy for mature DCs (CD11c^+^CD80^+^CD86^+^) cells in mouse lymph nodes.


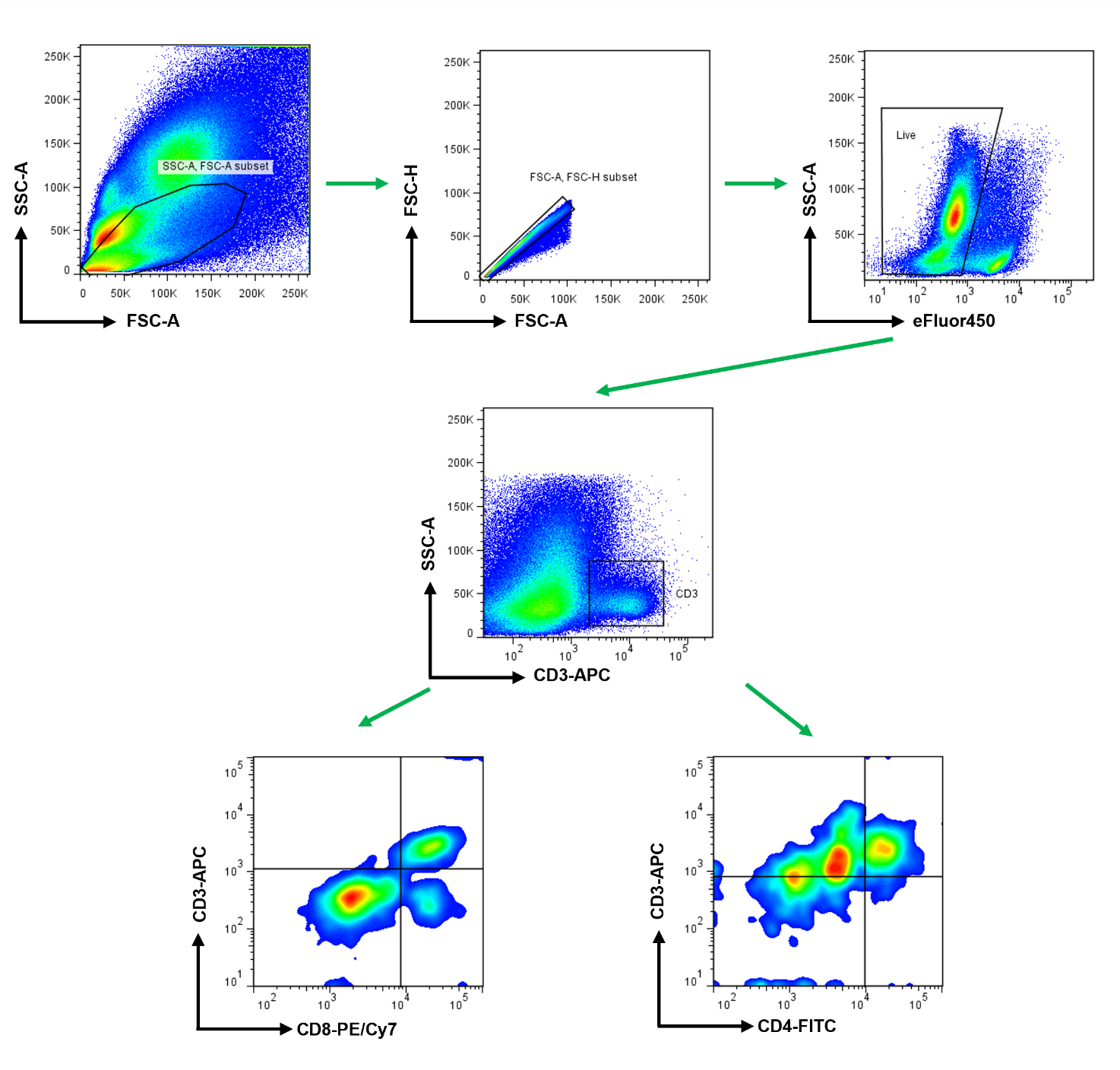


**Figure S22**. Gating strategy for cytotoxic CD8^+^ T cells and helper CD4^+^ T cells in tumors.


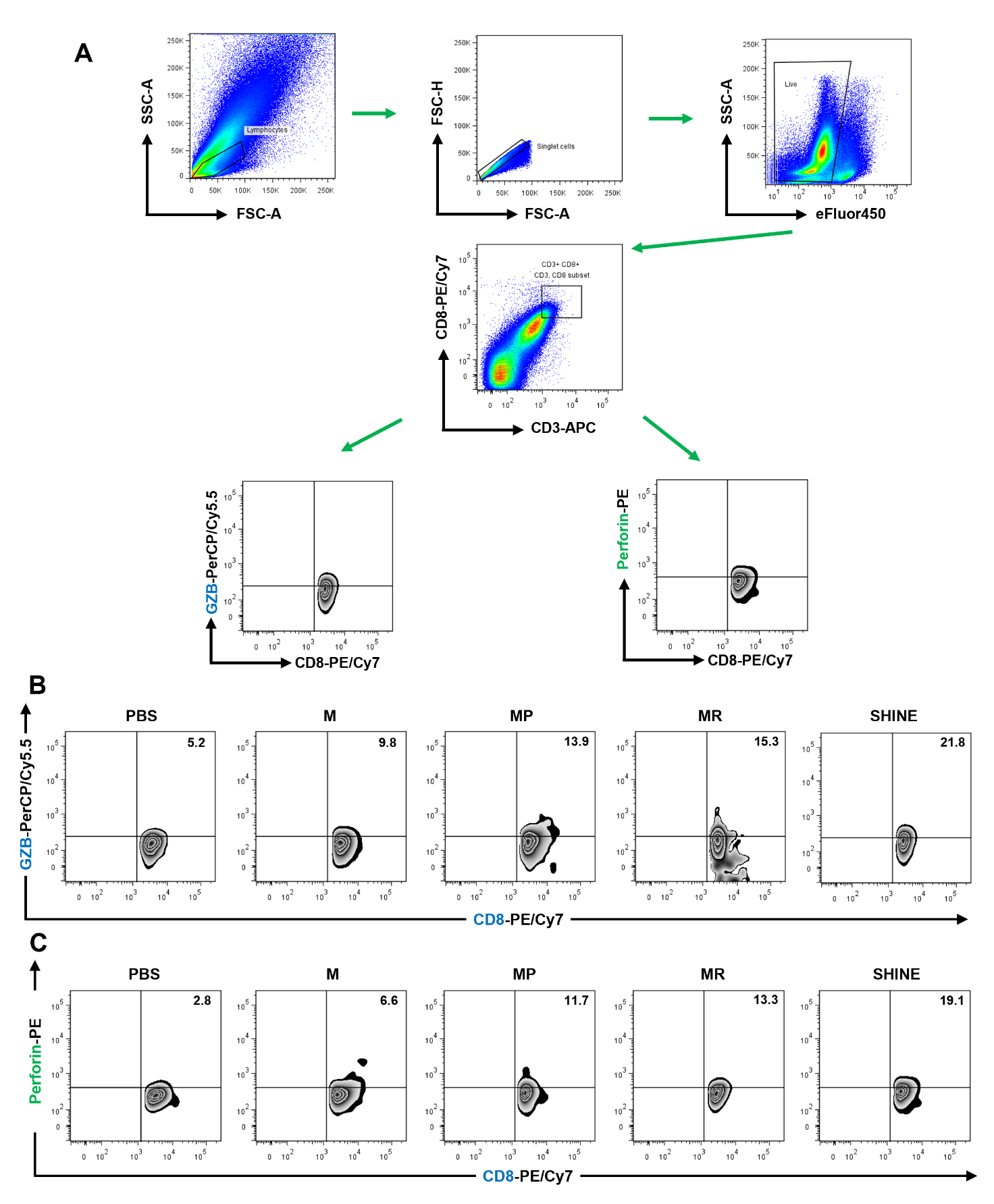


**Figure S23**. (**A**) Gating strategy for flow cytometric analysis of granzyme B (GZB) and perforin. Representative plots showing expression of (**B**) GZB and (**C**) perforin in tumor-infiltrating cells.


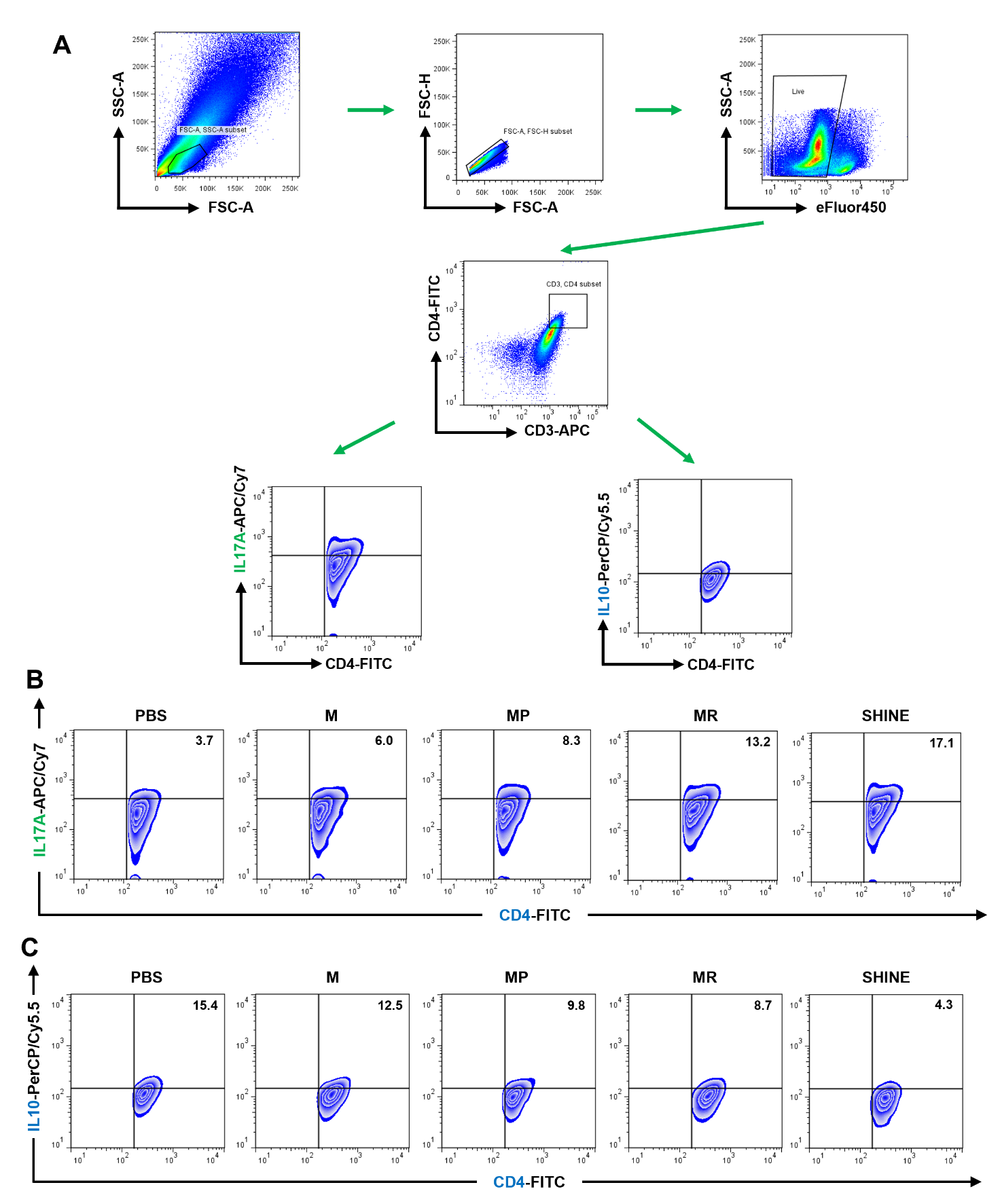


**Figure S24**. (**A**) Gating strategy for flow cytometric analysis of IL-17A and IL-10. Representative plots showing expression of (**B**) IL-17A and (**C**) IL-10 in tumor-infiltrating cells.


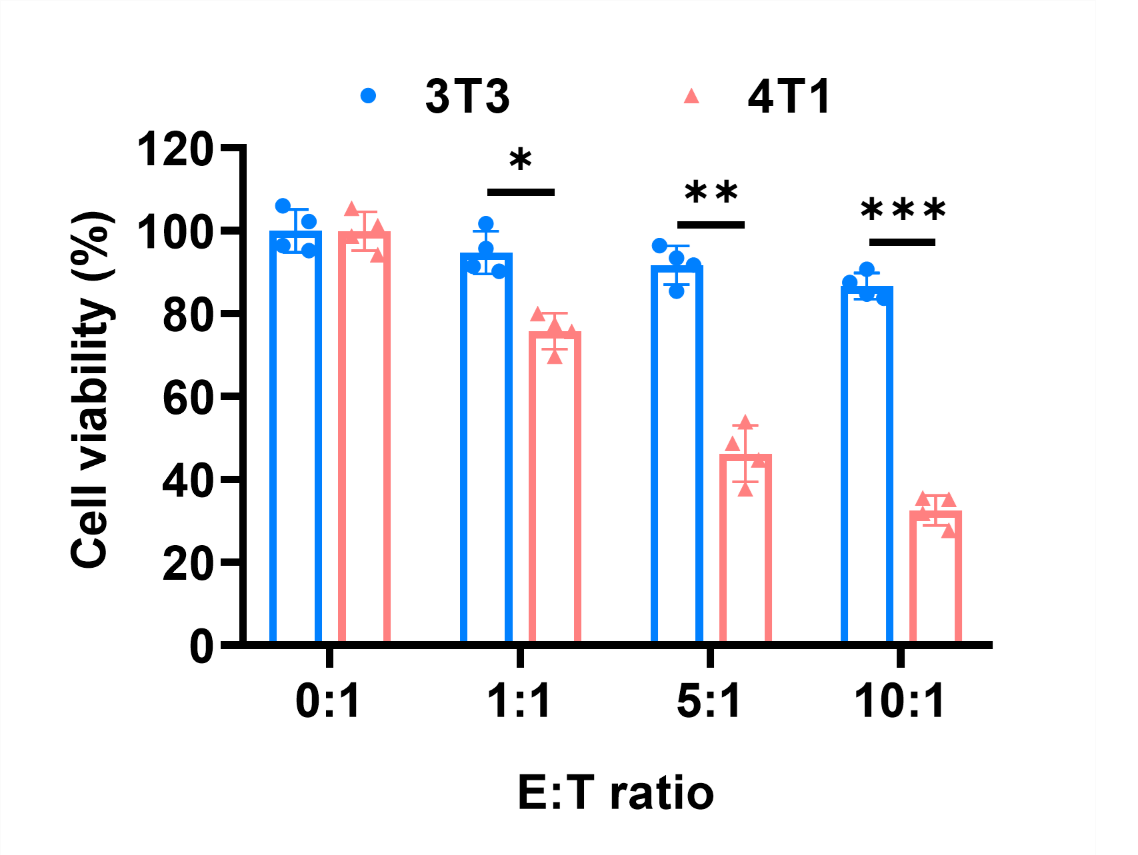


**Figure S25**. *In vitro* tumor cell killing assay using effector T cells isolated from tumors of SHINE-treated mice. Cell viability of 3T3 normal cells and 4T1 tumor cells after co-incubation with effector T cells at the indicated effector-to-target (E:T) ratios. Data show preferential and E:T ratio-dependent killing of 4T1 cells over 3T3 cells. Data are presented as the mean ± SD (*n = 4*). **p* < 0.05, ***p* < 0.01, ****p* < 0.001.


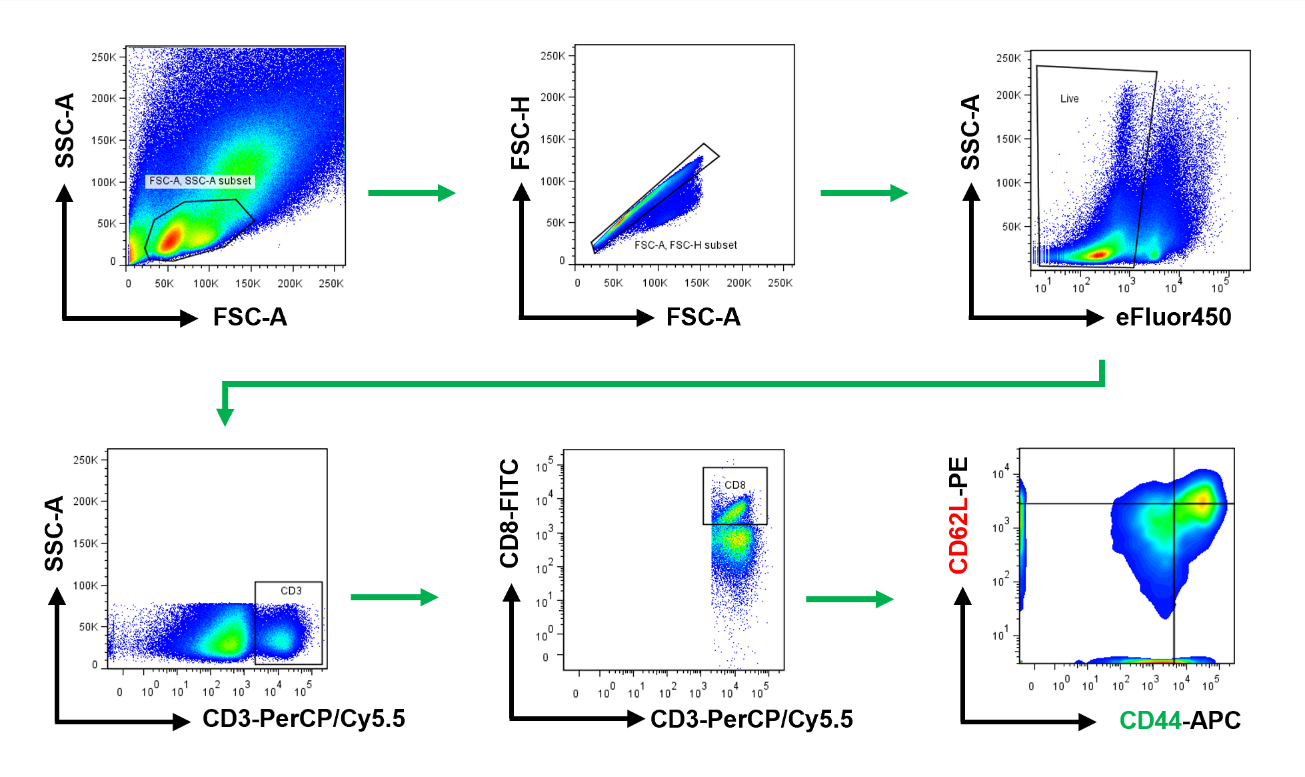


**Figure S26**. Gating strategy for effector memory T cells (T_EM_, CD3^+^CD8^+^CD44^+^CD62L^−^) in spleens.


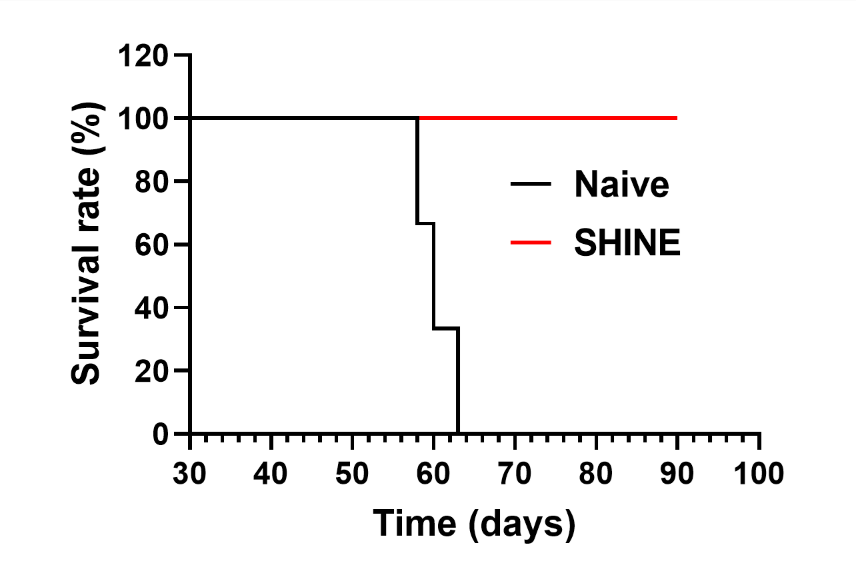


**Figure S27**. Survival curves of mice in the 4T1 tumor rechallenge model (*n = 6* per group).

**Table S1**. Pharmacokinetic parameters of SHINE in mice

| **Parameter** | **Units** | **Mean** |
| --- | --- | --- |
| t_1/2_(α) | h | 0.81 |
| t_1/2_(β) | h | 15.08 |
| AUC (0 – t) | mg·L^-1^·h | 161.72 |
| k_10_ | h^-1^ | 0.340 |
| k_12_ | h^-1^ | 0.442 |
| k_21_ | h^-1^ | 0.120 |

Following i.v. administration, SHINE displayed biexponential blood decay consistent with a two-compartment model. The rapid distribution phase (t_1/2_(α) = 0.81 h) indicates fast transfer from the bloodstream into peripheral tissues, whereas the prolonged terminal phase (t_1/2_(β) = 15.08 h) suggests sustained persistence of Mn-associated SHINE signal *in vivo*. The higher intercompartmental transfer rate from central to peripheral tissues (k_12_ = 0.442 h^-1^) relative to the reverse transfer (k_21_ = 0.120 h^-1^) further supports efficient tissue distribution and retention. Together with the substantial systemic exposure (AUC = 161.72 mg·L^-1^·h), these data indicate that SHINE remains available in circulation long enough to support tumor delivery while also undergoing substantial tissue partitioning after i.v. injection.

**Table S2**. Fractional tumor volume method for *in vivo* synergy*^a^*

|  | **PBS**  (control) | **MP**  (CDT + aPD-L1) | **MR**  (CDT + R848) | **SHINE**  (CDT + aPD-L1 + R848) |
| --- | --- | --- | --- | --- |
| V (mm^3^) | 3300.8 | 1885.9 | 1503.1 | 654.4 |
| FTV*^b^* | - | 0.571 | 0.455 | 0.198 |
| FTV_exp_*^c^* | 0.26 | | | |
| R*^d^* | 1.31 | | | |

*^a^In vivo* combination effects were evaluated using the fractional tumor volume (FTV) method at the study endpoint [2]. *^b^*FTV = V_t,20_/V_c,20_, where V_t,20_ and V_c,20_ denote the mean tumor volumes of the treated and control groups at day 20, respectively. *^c^*Expected additive effect (FTV_exp_), FTV_exp_ = FTV_MP_ × FTV_MR_. *^d^*Synergy ratio (R), R = FTV_exp_/FTV_SHINE_, where R > 1 indicates synergy and R = 1 indicates additivity.

**Table S3.** Comparison of SHINE with representative Mn-based CDT-Immunotherapy nanoplatforms

| **Formulation** | **Tumor-responsive feature** | **Immune activation strategy** | **Anti-metastatic efficacy** | **Rechallenge / recurrence prevention** | **Durable immune memory (evidence)** | **Ref.** | **Year** |
| --- | --- | --- | --- | --- | --- | --- | --- |
| SHINE (MnO_2_@R848@aPD-L1) | GSH-responsive MnO_2_ decomposition with GSH depletion and Mn^2+^ generation | CDT-induced ICD + R848-mediated innate immune activation + PD-L1 blockade | Yes | Yes | Yes (FACS; RNA-seq) | This work |  |
| MnP@LNP + aPD-L1 | pH-responsive in situ vaccine | ICD induction + cGAS/STING activation + PD-L1 blockade | Yes | Not reported | Yes (FACS) | [3] | 2026 |
| CD@H-MnO_2_ | GSH-responsive hollow MnO_2_ platform | ROS-mediated ICD induction + cGAS/STING activation | Not reported | Yes | Yes (FACS) | [4] | 2026 |
| HMn-NC@M | pH-responsive biomimetic nanomaterial | ICD induction + cGAS/STING activation | Yes | Not reported | Not reported | [5] | 2025 |
| gCM@MnAu | Cascade nanozyme platform | CDT-induced ICD + STING activation + checkpoint immunotherapy | Yes | Not reported | Not reported | [6] | 2024 |
| ISAMn-MOF | pH-responsive MOF | Immunostimulation + cGAS/STING activation | Yes | Not reported | Not reported | [7] | 2023 |
| aPDL1@MnO_2_ | Radiotherapy-amplified / RT-responsive system | RT-induced ICD + PD-L1 blockade + STING activation | Yes | Not reported | Not reported | [8] | 2023 |
| MnO@mSiO_2_-iRGD NPs | pH-responsive targeted platform | CDT + immune checkpoint blockade + STING activation | Yes | Not reported | Not reported | [9] | 2022 |
| M-M NPs | GSH-responsive platform | ROS generation + APC maturation | Yes | Not reported | Not reported | [10] | 2022 |
| MnO_x_ nanospikes (NSs) | GSH-responsive platform | CDT + ICD | Yes | Not reported | Not reported | [11] | 2020 |
| MS@MnO_2_ NPs | GSH depletion-enhanced CDT | Primarily CDT | Not reported | Not reported | Not reported | [12] | 2018 |

**Supplementary Note**

**Note S1**: **Estimation of the number of aPD-L1 antibodies per MnO_2_@R848@aPD-L1 (SHINE) nanoparticle (NP)**

Based on transmission electron microscopy (TEM) analysis, SHINE exhibited an average outer diameter of 110 nm and a shell thickness of 12 nm, corresponding to an outer radius (R) of 55 nm and an inner radius (r) of 43 nm. The shell volume of a single hollow nanoparticle was therefore calculated as:

V_shell_ = 4/3π(R^3^ – r^3^) = 4/3×3.14×(55^3^ – 43^3^) = 3.64×10^5^ nm^3^ = 3.64×10^-16^ cm^3^

Because the hollow MnO_2_ shell exhibited a nanosheet-assembled morphology characteristic of layered δ-MnO_2_, a literature density for birnessite-like δ-MnO_2_ was used as an approximation of ρ(δ-MnO_2_) = 3.0 g/cm^3^ to estimate particle number [13,14]. The mass of a single SHINE nanoparticle was thus calculated as:

m_NP_ = ρ×V_shell_ = 3.0×3.64×10^-16^ = 1.09×10^-15^ g

Accordingly, the number of SHINE NPs in 1 mg of NPs was estimated to be:

N_NP_ = 1×10^-3^/(1.09×10^-15^) = 9.15×10^11^ NP

From the aPD-L1 conjugation optimization experiment, the antibody loading was determined to be 112.6 ± 8.7 μg aPD-L1 per mg NPs. Molecular weight of 150,000 g/mol for aPD-L1, thus, the total number of antibody molecules in 1 mg NPs was calculated as:

N_Ab_ = 112.6×10^-6^×6.022×10^23^/150000 = 4.52×10^14^ molecules

Therefore, the number of aPD-L1 antibodies per SHINE NP was estimated to be:

Ab per NP = 4.52×10^14^/(9.15×10^11^) = 4.95×10^2^.

Thus, the average number of surface-conjugated aPD-L1 molecules was estimated to be approximately 495 per SHINE NP.

**References**

[1] N. Obermajer, J. Urban, E. Wieckowski, R. Muthuswamy, R. Ravindranathan, D.L. Bartlett, P. Kalinski, Promoting the accumulation of tumor-specific T cells in tumor tissues by dendritic cell vaccines and chemokine-modulating agents, Nat. Protoc. 13 (2018) 335–357. https://doi.org/10.1038/nprot.2017.130.

[2] M. Malvicini, M. Rizzo, L. Alaniz, F. Piñero, M. García, C. Atorrasagasti, J.B. Aquino, V. Rozados, O.G. Scharovsky, P. Matar, G. Mazzolini, A Novel Synergistic Combination of Cyclophosphamide and Gene Transfer of Interleukin-12 Eradicates Colorectal Carcinoma in Mice, Clin. Cancer Res. 15 (2009) 7256–7265. https://doi.org/10.1158/1078-0432.CCR-09-1861.

[3] Q. Yang, X. Shi, K. Yang, Q. Gao, Y. Cao, Y. Huang, L. Chen, S. Bao, L. Xu, R.L. Reis, S.C. Kundu, H. Xu, B. Xiao, A manganese-based nanoplatform leveraging chemodynamic and adjuvant effects for in situ vaccination against colorectal cancer, Biomaterials 331 (2026) 124085. https://doi.org/10.1016/j.biomaterials.2026.124085.

[4] J. Zhao, J. Cai, J. Hu, Z. Zhang, Y.-Y. Liu, D. Pan, L. Shen, B. Geng, Biodegradable hollow MnO2 decorated by carbon dots with cholesterol depletion capability for cascaded amplification of sono-immunotherapy, Biomaterials 325 (2026) 123559. https://doi.org/10.1016/j.biomaterials.2025.123559.

[5] C. Ren, H. Zhang, M. Yang, Y. Huang, J. Guo, K. Chen, Y. Fu, H. Chen, Y. Cao, R. Hao, H. Hou, Membrane-Coated Hollow Manganese Nitrogen Carbon Nanocomposites Synergize Phototherapy and STING Activation for Immunogenic Tumor Microenvironment Remodeling, ACS Appl. Mater. Interfaces 17 (2025) 37053–37067. https://doi.org/10.1021/acsami.5c05076.

[6] J. Zhang, Y. Pan, L. Liu, Y. Xu, C. Zhao, W. Liu, L. Rao, Genetically Edited Cascade Nanozymes for Cancer Immunotherapy, ACS Nano 18 (2024) 12295–12310. https://doi.org/10.1021/acsnano.4c01229.

[7] S.-J. Zheng, M. Yang, J.-Q. Luo, R. Liu, J. Song, Y. Chen, J.-Z. Du, Manganese-Based Immunostimulatory Metal–Organic Framework Activates the cGAS-STING Pathway for Cancer Metalloimmunotherapy, ACS Nano 17 (2023) 15905–15917. https://doi.org/10.1021/acsnano.3c03962.

[8] Z. Deng, M. Xi, C. Zhang, X. Wu, Q. Li, C. Wang, H. Fang, G. Sun, Y. Zhang, G. Yang, Z. Liu, Biomineralized MnO2 Nanoplatforms Mediated Delivery of Immune Checkpoint Inhibitors with STING Pathway Activation to Potentiate Cancer Radio-Immunotherapy, ACS Nano 17 (2023) 4495–4506. https://doi.org/10.1021/acsnano.2c10352.

[9] Z. Sun, Z. Wang, T. Wang, J. Wang, H. Zhang, Z. Li, S. Wang, F. Sheng, J. Yu, Y. Hou, Biodegradable MnO-Based Nanoparticles with Engineering Surface for Tumor Therapy: Simultaneous Fenton-Like Ion Delivery and Immune Activation, ACS Nano 16 (2022) 11862–11875. https://doi.org/10.1021/acsnano.2c00969.

[10] S. Tang, L. Zhou, H. He, L. Cui, Z. Ren, Y. Tai, Z. Xie, Y. Cao, D. Meng, Q. Liu, Y. Wu, J. Jiang, X. Zhou, MnO2-melittin nanoparticles serve as an effective anti-tumor immunotherapy by enhancing systemic immune response, Biomaterials 288 (2022) 121706. https://doi.org/10.1016/j.biomaterials.2022.121706.

[11] B. Ding, P. Zheng, F. Jiang, Y. Zhao, M. Wang, M. Chang, P. Ma, J. Lin, MnOx Nanospikes as Nanoadjuvants and Immunogenic Cell Death Drugs with Enhanced Antitumor Immunity and Antimetastatic Effect, Angew. Chemie Int. Ed. 59 (2020) 16381–16384. https://doi.org/10.1002/anie.202005111.

[12] L.-S. Lin, J. Song, L. Song, K. Ke, Y. Liu, Z. Zhou, Z. Shen, J. Li, Z. Yang, W. Tang, G. Niu, H.-H. Yang, X. Chen, Simultaneous Fenton-like Ion Delivery and Glutathione Depletion by MnO2-Based Nanoagent to Enhance Chemodynamic Therapy, Angew. Chemie Int. Ed. 57 (2018) 4902–4906. https://doi.org/10.1002/anie.201712027.

[13] A. Greene, J. Hashemi, Y. Kang, Development of MnO2 hollow nanoparticles for potential drug delivery applications, Nanotechnology 32 (2021) 25713. https://doi.org/10.1088/1361-6528/abb626.

[14] J.E. Post, D.R. Veblen, Crystal structure determinations of synthetic sodium, magnesium, and potassium birnessite using TEM and the Rietveld method, Am. Mineral. 75 (1990) 477–489.
