# Supplementary material for: GSH-responsive nanovaccine triggers immunogenic cell death and potent memory T cell immunity for durable, recurrence-free tumor eradication
Source: Bioact Mater. 2026 Apr 30;64:135–58. doi: 10.1016/j.bioactmat.2026.04.025 (PMC13145900; doi:10.1016/j.bioactmat.2026.04.025)
Supplement: Multimedia component 1 [file mmc1.zip › Table S1-S3-Note S1.docx]

**Table S1**. Pharmacokinetic parameters of SHINE in mice

| **Parameter** | **Units** | **Mean** |
| --- | --- | --- |
| t_1/2_(α) | h | 0.81 |
| t_1/2_(β) | h | 15.08 |
| AUC (0 – t) | mg·L^-1^·h | 161.72 |
| k_10_ | h^-1^ | 0.340 |
| k_12_ | h^-1^ | 0.442 |
| k_21_ | h^-1^ | 0.120 |

Following i.v. administration, SHINE displayed biexponential blood decay consistent with a two-compartment model. The rapid distribution phase (t_1/2_(α) = 0.81 h) indicates fast transfer from the bloodstream into peripheral tissues, whereas the prolonged terminal phase (t_1/2_(β) = 15.08 h) suggests sustained persistence of Mn-associated SHINE signal *in vivo*. The higher intercompartmental transfer rate from central to peripheral tissues (k_12_ = 0.442 h^-1^) relative to the reverse transfer (k_21_ = 0.120 h^-1^) further supports efficient tissue distribution and retention. Together with the substantial systemic exposure (AUC = 161.72 mg·L^-1^·h), these data indicate that SHINE remains available in circulation long enough to support tumor delivery while also undergoing substantial tissue partitioning after i.v. injection.

**Table S2**. Fractional tumor volume method for *in vivo* synergy*^a^*

|  | **PBS**  (control) | **MP**  (CDT + aPD-L1) | **MR**  (CDT + R848) | **SHINE**  (CDT + aPD-L1 + R848) |
| --- | --- | --- | --- | --- |
| V (mm^3^) | 3300.8 | 1885.9 | 1503.1 | 654.4 |
| FTV*^b^* | - | 0.571 | 0.455 | 0.198 |
| FTV_exp_*^c^* | 0.26 | | | |
| R*^d^* | 1.31 | | | |

*^a^In vivo* combination effects were evaluated using the fractional tumor volume (FTV) method at the study endpoint [2]. *^b^*FTV = V_t,20_/V_c,20_, where V_t,20_ and V_c,20_ denote the mean tumor volumes of the treated and control groups at day 20, respectively. *^c^*Expected additive effect (FTV_exp_), FTV_exp_ = FTV_MP_ × FTV_MR_. *^d^*Synergy ratio (R), R = FTV_exp_/FTV_SHINE_, where R > 1 indicates synergy and R = 1 indicates additivity.

**Table S3.** Comparison of SHINE with representative Mn-based CDT-Immunotherapy nanoplatforms

| **Formulation** | **Tumor-responsive feature** | **Immune activation strategy** | **Anti-metastatic efficacy** | **Rechallenge / recurrence prevention** | **Durable immune memory (evidence)** | **Ref.** | **Year** |
| --- | --- | --- | --- | --- | --- | --- | --- |
| SHINE (MnO_2_@R848@aPD-L1) | GSH-responsive MnO_2_ decomposition with GSH depletion and Mn^2+^ generation | CDT-induced ICD + R848-mediated innate immune activation + PD-L1 blockade | Yes | Yes | Yes (FACS; RNA-seq) | This work |  |
| MnP@LNP + aPD-L1 | pH-responsive in situ vaccine | ICD induction + cGAS/STING activation + PD-L1 blockade | Yes | Not reported | Yes (FACS) | [3] | 2026 |
| CD@H-MnO_2_ | GSH-responsive hollow MnO_2_ platform | ROS-mediated ICD induction + cGAS/STING activation | Not reported | Yes | Yes (FACS) | [4] | 2026 |
| HMn-NC@M | pH-responsive biomimetic nanomaterial | ICD induction + cGAS/STING activation | Yes | Not reported | Not reported | [5] | 2025 |
| gCM@MnAu | Cascade nanozyme platform | CDT-induced ICD + STING activation + checkpoint immunotherapy | Yes | Not reported | Not reported | [6] | 2024 |
| ISAMn-MOF | pH-responsive MOF | Immunostimulation + cGAS/STING activation | Yes | Not reported | Not reported | [7] | 2023 |
| aPDL1@MnO_2_ | Radiotherapy-amplified / RT-responsive system | RT-induced ICD + PD-L1 blockade + STING activation | Yes | Not reported | Not reported | [8] | 2023 |
| MnO@mSiO_2_-iRGD NPs | pH-responsive targeted platform | CDT + immune checkpoint blockade + STING activation | Yes | Not reported | Not reported | [9] | 2022 |
| M-M NPs | GSH-responsive platform | ROS generation + APC maturation | Yes | Not reported | Not reported | [10] | 2022 |
| MnO_x_ nanospikes (NSs) | GSH-responsive platform | CDT + ICD | Yes | Not reported | Not reported | [11] | 2020 |
| MS@MnO_2_ NPs | GSH depletion-enhanced CDT | Primarily CDT | Not reported | Not reported | Not reported | [12] | 2018 |

**Supplementary Note**

**Note S1**: **Estimation of the number of aPD-L1 antibodies per MnO_2_@R848@aPD-L1 (SHINE) nanoparticle (NP)**

Based on transmission electron microscopy (TEM) analysis, SHINE exhibited an average outer diameter of 110 nm and a shell thickness of 12 nm, corresponding to an outer radius (R) of 55 nm and an inner radius (r) of 43 nm. The shell volume of a single hollow nanoparticle was therefore calculated as:

V_shell_ = 4/3π(R^3^ – r^3^) = 4/3×3.14×(55^3^ – 43^3^) = 3.64×10^5^ nm^3^ = 3.64×10^-16^ cm^3^

Because the hollow MnO_2_ shell exhibited a nanosheet-assembled morphology characteristic of layered δ-MnO_2_, a literature density for birnessite-like δ-MnO_2_ was used as an approximation of ρ(δ-MnO_2_) = 3.0 g/cm^3^ to estimate particle number [13,14]. The mass of a single SHINE nanoparticle was thus calculated as:

m_NP_ = ρ×V_shell_ = 3.0×3.64×10^-16^ = 1.09×10^-15^ g

Accordingly, the number of SHINE NPs in 1 mg of NPs was estimated to be:

N_NP_ = 1×10^-3^/(1.09×10^-15^) = 9.15×10^11^ NP

From the aPD-L1 conjugation optimization experiment, the antibody loading was determined to be 112.6 ± 8.7 μg aPD-L1 per mg NPs. Molecular weight of 150,000 g/mol for aPD-L1, thus, the total number of antibody molecules in 1 mg NPs was calculated as:

N_Ab_ = 112.6×10^-6^×6.022×10^23^/150000 = 4.52×10^14^ molecules

Therefore, the number of aPD-L1 antibodies per SHINE NP was estimated to be:

Ab per NP = 4.52×10^14^/(9.15×10^11^) = 4.95×10^2^.

Thus, the average number of surface-conjugated aPD-L1 molecules was estimated to be approximately 495 per SHINE NP.
